# Safety and long-term immunogenicity of the two-dose heterologous Ad26.ZEBOV and MVA-BN-Filo Ebola vaccine regimen in adults in Sierra Leone: a combined open-label, non-randomised stage 1, and a randomised, double-blind, controlled stage 2 trial

**DOI:** 10.1016/S1473-3099(21)00125-0

**Published:** 2021-09-13

**Authors:** David Ishola, Daniela Manno, Muhammed O Afolabi, Babajide Keshinro, Viki Bockstal, Baimba Rogers, Kwabena Owusu-Kyei, Alimamy Serry-Bangura, Ibrahim Swaray, Brett Lowe, Dickens Kowuor, Frank Baiden, Thomas Mooney, Elizabeth Smout, Brian Köhn, Godfrey T Otieno, Morrison Jusu, Julie Foster, Mohamed Samai, Gibrilla Fadlu Deen, Heidi Larson, Shelley Lees, Neil Goldstein, Katherine E Gallagher, Auguste Gaddah, Dirk Heerwegh, Benoit Callendret, Kerstin Luhn, Cynthia Robinson, Maarten Leyssen, Brian Greenwood, Macaya Douoguih, Bailah Leigh, Deborah Watson-Jones

**Affiliations:** London School of Hygiene & Tropical Medicine, London, UK; EBOVAC Project, Kambia, Kambia district, Sierra Leone; London School of Hygiene & Tropical Medicine, London, UK; London School of Hygiene & Tropical Medicine, London, UK; EBOVAC Project, Kambia, Kambia district, Sierra Leone; Janssen Vaccines and Prevention BV, Leiden, Netherlands; EBOVAC Project, Kambia, Kambia district, Sierra Leone; College of Medicine and Allied Health Sciences, University of Sierra Leone, Freetown, Sierra Leone; London School of Hygiene & Tropical Medicine, London, UK; EBOVAC Project, Kambia, Kambia district, Sierra Leone; EBOVAC Project, Kambia, Kambia district, Sierra Leone; College of Medicine and Allied Health Sciences, University of Sierra Leone, Freetown, Sierra Leone; London School of Hygiene & Tropical Medicine, London, UK; KEMRI-Wellcome Trust Research Programme, Kilifi, Kenya; Centre for Tropical Medicine and Global Health, University of Oxford, Oxford, UK; London School of Hygiene & Tropical Medicine, London, UK; EBOVAC Project, Kambia, Kambia district, Sierra Leone; London School of Hygiene & Tropical Medicine, London, UK; EBOVAC Project, Kambia, Kambia district, Sierra Leone; College of Medicine and Allied Health Sciences, University of Sierra Leone, Freetown, Sierra Leone; London School of Hygiene & Tropical Medicine, London, UK; College of Medicine and Allied Health Sciences, University of Sierra Leone, Freetown, Sierra Leone; College of Medicine and Allied Health Sciences, University of Sierra Leone, Freetown; London School of Hygiene & Tropical Medicine, London, UK; Department of Health Metrics Sciences, University of Washington, Seattle, WA, USA; London School of Hygiene & Tropical Medicine, London, UK; Janssen Vaccines and Prevention BV, Leiden, Netherlands; London School of Hygiene & Tropical Medicine, London, UK; Janssen Research & Development, Beerse, Belgium; Janssen Vaccines and Prevention BV, Leiden, Netherlands; London School of Hygiene & Tropical Medicine, London, UK; Janssen Vaccines and Prevention BV, Leiden, Netherlands; College of Medicine and Allied Health Sciences, University of Sierra Leone, Freetown, Sierra Leone; London School of Hygiene & Tropical Medicine, London, UK; Mwanza Intervention Trials Unit, National Institute for Medical Research, Mwanza, Tanzania

## Abstract

**Background:**

The Ebola epidemics in west Africa and the Democratic Republic of the Congo highlight an urgent need for safe and effective vaccines to prevent Ebola virus disease. We aimed to assess the safety and long-term immunogenicity of a two-dose heterologous vaccine regimen, comprising the adenovirus type 26 vector-based vaccine encoding the Ebola virus glycoprotein (Ad26.ZEBOV) and the modified vaccinia Ankara vector-based vaccine, encoding glycoproteins from Ebola virus, Sudan virus, and Marburg virus, and the nucleoprotein from the Tai Forest virus (MVA-BN-Filo), in Sierra Leone, a country previously affected by Ebola.

**Methods:**

The trial comprised two stages: an open-label, non-randomised stage 1, and a randomised, double-blind, controlled stage 2. The study was done at three clinics in Kambia district, Sierra Leone. In stage 1, healthy adults (aged ≥18 years) residing in or near Kambia district, received an intramuscular injection of Ad26.ZEBOV (5×10^10^ viral particles) on day 1 (first dose) followed by an intramuscular injection of MVA-BN-Filo (1×10^8^ infectious units) on day 57 (second dose). An Ad26.ZEBOV booster vaccination was offered at 2 years after the first dose to stage 1 participants. The eligibility criteria for adult participants in stage 2 were consistent with stage 1 eligibility criteria. Stage 2 participants were randomly assigned (3:1), by computer-generated block randomisation (block size of eight) via an interactive web-response system, to receive either the Ebola vaccine regimen (Ad26.ZEBOV followed by MVA-BN-Filo) or an intramuscular injection of a single dose of meningococcal quadrivalent (serogroups A, C, W135, and Y) conjugate vaccine (MenACWY; first dose) followed by placebo on day 57 (second dose; control group). Study team personnel, except those with primary responsibility for study vaccine preparation, and participants were masked to study vaccine allocation. The primary outcome was the safety of the Ad26.ZEBOV and MVA-BN-Filo vaccine regimen, which was assessed in all participants who had received at least one dose of study vaccine. Safety was assessed as solicited local and systemic adverse events occurring in the first 7 days after each vaccination, unsolicited adverse events occurring in the first 28 days after each vaccination, and serious adverse events or immediate reportable events occurring up to each participant’s last study visit. Secondary outcomes were to assess Ebola virus glycoprotein-specific binding antibody responses at 21 days after the second vaccine in a per-protocol set of participants (ie, those who had received both vaccinations within the protocol-defined time window, had at least one evaluable post-vaccination sample, and had no major protocol deviations that could have influenced the immune response) and to assess the safety and tolerability of the Ad26.ZEBOV booster vaccination in stage 1 participants who had received the booster dose. This study is registered at ClinicalTrials.gov, NCT02509494.

**Findings:**

Between Sept 30, 2015, and Oct 19, 2016, 443 participants (43 in stage 1 and 400 in stage 2) were enrolled; 341 participants assigned to receive the Ad26.ZEBOV and MVA-BN-Filo regimen and 102 participants assigned to receive the MenACWY and placebo regimen received at least one dose of study vaccine. Both regimens were well tolerated with no safety concerns. In stage 1, solicited local adverse events (mostly mild or moderate injection-site pain) were reported in 12 (28%) of 43 participants after Ad26.ZEBOV vaccination and in six (14%) participants after MVA-BN-Filo vaccination. In stage 2, solicited local adverse events were reported in 51 (17%) of 298 participants after Ad26.ZEBOV vaccination, in 58 (24%) of 246 after MVA-BN-Filo vaccination, in 17 (17%) of 102 after MenACWY vaccination, and in eight (9%) of 86 after placebo injection. In stage 1, solicited systemic adverse events were reported in 18 (42%) of 43 participants after Ad26.ZEBOV vaccination and in 17 (40%) after MVA-BN-Filo vaccination. In stage 2, solicited systemic adverse events were reported in 161 (54%) of 298 participants after Ad26.ZEBOV vaccination, in 107 (43%) of 246 after MVA-BN-Filo vaccination, in 51 (50%) of 102 after MenACWY vaccination, and in 39 (45%) of 86 after placebo injection. Solicited systemic adverse events in both stage 1 and 2 participants included mostly mild or moderate headache, myalgia, fatigue, and arthralgia. The most frequent unsolicited adverse event after the first dose was headache in stage 1 and malaria in stage 2. Malaria was the most frequent unsolicited adverse event after the second dose in both stage 1 and 2. No serious adverse event was considered related to the study vaccine, and no immediate reportable events were observed. In stage 1, the safety profile after the booster vaccination was not notably different to that observed after the first dose. Vaccine-induced humoral immune responses were observed in 41 (98%) of 42 stage 1 participants (geometric mean binding antibody concentration 4784 ELISA units [EU]/mL [95% CI 3736–6125]) and in 176 (98%) of 179 stage 2 participants (3810 EU/mL [3312–4383]) at 21 days after the second vaccination.

**Interpretation:**

The Ad26.ZEBOV and MVA-BN-Filo vaccine regimen was well tolerated and immunogenic, with persistent humoral immune responses. These data support the use of this vaccine regimen for Ebola virus disease prophylaxis in adults.

**Funding:**

Innovative Medicines Initiative 2 Joint Undertaking and Janssen Vaccines & Prevention BV.

## Introduction

The magnitude of the Ebola virus outbreak in 2014–16 in west Africa was unprecedented, with more than 28 600 cases reported and 11 300 deaths.^1^ The second largest outbreak began in 2018 in the Democratic Republic of the Congo and lasted for nearly 2 years, with more than 3400 cases and 2200 deaths reported.^2^ Other small Ebola virus disease outbreaks have occurred since then in the DR Congo and Guinea, and new outbreaks are likely to occur in the future.^3^ Therefore, finding safe and effective vaccines against Ebola virus disease that can be used in combination with other outbreak control measures remains a priority. The recombinant vesicular stomatitis virus-vectored vaccine expressing the Ebola virus glycoprotein (rVSV-ZEBOV-GP) of the Kikwit strain, which showed effectiveness in a ring-vaccination trial done in Guinea during the 2014–16 outbreak,^4^ was recommended by WHO for use in emergency situations, and was deployed widely as part of the outbreak control response in DR Congo.^5,6^ This vaccine received conditional marketing authorisation in the EU, and was approved for use in adults in the USA and in several African countries.^7–9^ However, as part of the preparedness measures for future outbreaks, the Strategic Advisory Group of Experts on Immunization recommended to WHO that urgent consideration should be given to the development of additional vaccines against Ebola, with a focus on safety and induction of appropriate immune responses.^6^

A heterologous, two-dose regimen, comprising the monovalent, recombinant, replication-incompetent, adenovirus type 26 (Ad26) vector-based vaccine, encoding the Ebola virus glycoprotein of the Mayinga variant (Ad26.ZEBOV) as the first vaccine, and the recombinant, non-replicating, modified vaccinia Ankara (MVA) vector-based vaccine, encoding glycoproteins from the Ebola virus Mayinga variant, Sudan virus Gulu variant, and Marburg virus Musoke variant, and the nucleoprotein from the Tai Forest virus (MVA-BN-Filo) administered 56 days after the first vaccine, has received marketing authorisation for prophylactic use, under exceptional circumstances, in adults and children aged 1 year and older in the EU.^10^ This vaccine regimen provided protection against Ebola virus challenge in macaques and had a good safety profile, with strong and durable immune responses observed for at least 1 year in European and healthy African adults living in areas unaffected by Ebola.^11–15^ In this study, we aimed to evaluate the safety, long-term immunogenicity, and humoral immune memory induced by the Ad26.ZEBOV and MVA-BN-Filo vaccine regimen, administered with a 56-day interval between the two doses, in healthy adults in Kambia district, an area of Sierra Leone affected by the 2014–16 Ebola virus disease epidemic and, therefore, at potential risk for future outbreaks.^16^

## Methods

### Study design

The trial comprised two stages: an open-label, non-randomised stage 1, and a randomised, double-blind, controlled stage 2. The trial was done at three clinics in Kambia district. The rationale for an open-label stage 1 trial was to obtain initial safety data, as it was the first time that the experimental Ad26.ZEBOV and MVA-BN-Filo vaccine regimen was used in Sierra Leone, and the national health authority requested the inclusion of this initial stage in the study design. Enrolment of stage 1 participants was followed by initiation of stage 2 after review of stage 1 safety data by an independent data monitoring committee. The study was approved by the Sierra Leone Ethics and Scientific Review Committee, the Pharmacy Board of Sierra Leone, and the London School of Hygiene & Tropical Medicine ethics committee. The study protocol is available in the appendix (pp 25–154).

### Participants

Eligible stage 1 participants were healthy adults aged 18 years or older residing in or near Kambia district, with no intention of leaving the area within the next 5 months, and who were considered healthy on the basis of physical examination and the absence of laboratory abnormalities at screening. Women of childbearing age were required to use adequate birth control measures (ie, contraceptive injection, oral contraception, or barrier methods) from at least 14 days before receiving the first vaccine, and to have a negative urine β-human chorionic gonadotropin pregnancy test at screening and immediately before each vaccination. Male participants who were sexually active were asked to use condoms, starting before enrolment. Exclusion criteria included breast feeding or pregnancy; previous Ebola virus disease or vaccination with a candidate Ebola vaccine; previous vaccination with a live-attenuated vaccine within 30 days before each dose, or with an inactivated vaccine within 15 days before each dose; or a previous severe adverse reaction to a vaccine. Extensive social science research was done before the start of the trial to ensure effective community engagement and the use of appropriate recruitment strategies.^17,18^ Written informed consent from a community leader was required before the study start. Participants provided informed consent after passing a test of understanding. If the participant was unable to read or write, the procedures were explained, and informed consent was witnessed by a literate third person not involved in the study. Inclusion and exclusion criteria, and the procedures for obtaining written informed consent for stage 2 adult participants were similar to those for stage 1 participants. Stage 2 also enrolled children aged 1–17 years, and data from these paediatric cohorts are presented in a separate publication.^19^ The full list of inclusion and exclusion criteria is provided in the study protocol (appendix pp 84–88).

### Randomisation and masking

There was no randomisation in stage 1. Stage 2 participants were randomly assigned (3:1) to receive either Ad26.ZEBOV and MVA-BN-Filo (Ebola vaccine group) or the meningococcal quadrivalent (serogroups A, C, W135, and Y) conjugate vaccine (MenACWY) and placebo (control group). Randomisation was done centrally by computer-generated block randomisation (block size of eight) via an interactive web response system, which was operated by a study pharmacist. Study team personnel (except those with primary responsibility for study vaccine preparation) and participants were masked to study vaccine allocation until all participants had completed the last follow-up visit and the database was locked. Masking was achieved by use of syringes of identical volume, which were taped to conceal the colour of the liquid inside.

### Procedures

**Figure 1:**
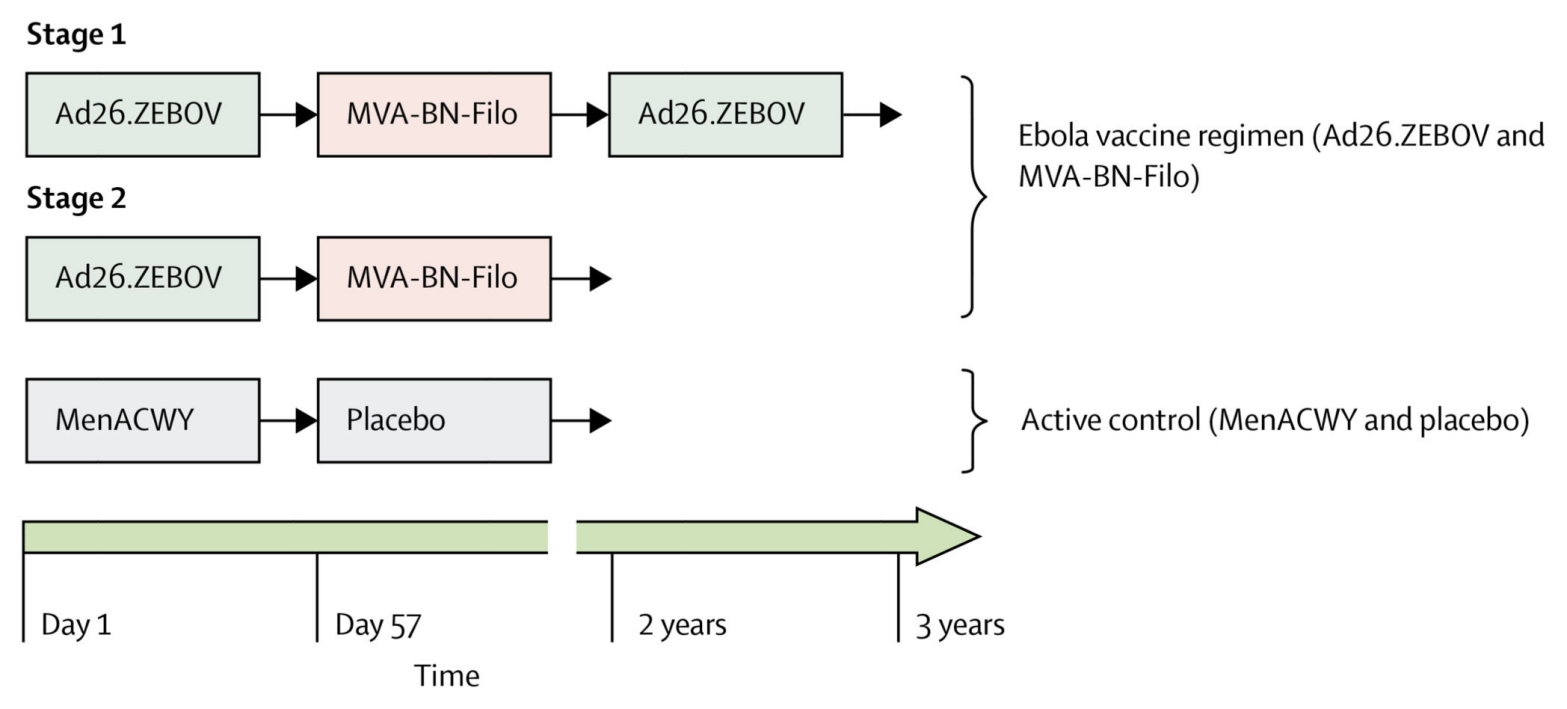
Study design Vaccine doses were 5×10^10^ viral particles for Ad26.ZEBOV, 1×10^8^ infectious units for MVA-BN-Filo, 0·5 mL reconstituted vaccine solution for MenACWY, and 0·5 mL of 0·9% sodium chloride solution for the placebo. Ad26. ZEBOV=adenovirus type 26 vector-based vaccine encoding the Ebola virus glycoprotein. MenACWY=meningococcal quadrivalent (serogroups A, C, W135, and Y) conjugate vaccine. MVA-BN-Filo=modified vaccinia Ankara vector-based vaccine, encoding glycoproteins from the Ebola virus, Sudan virus, and Marburg virus, and the nucleoprotein from the Tai Forest virus.

In stage 1, all participants received Ad26.ZEBOV (Janssen Vaccines & Prevention BV, Leiden, Netherlands; first dose) followed by MVA-BN-Filo (Bavarian Nordic, Planegg, Germany; second dose) 56 days after the first dose. An Ad26.ZEBOV booster vaccination was also offered to stage 1 participants at 2 years (720 days) after the first dose (figure 1). Stage 2 adult participants in the Ebola vaccine group received the Ebola vaccine regimen (Ad26.ZEBOV followed by MVA-BN-Filo), and those in the control group received one dose of the MenACWY vaccine (Menveo [GSK Vaccines, Brentford, UK]; or Nimenrix [Pfizer, New York, NY, USA; first dose]) followed by a saline placebo (second dose) at 56 days after the first dose (figure 1). All vaccines were administered as a single 0·5 mL intramuscular injection into the deltoid muscle at a dose of 5×10^10^ viral particles for Ad26. ZEBOV, 1 ×10^8^ infectious units for MVA-BN-Filo, 0·5 mL reconstituted vaccine solution for MenACWY, and 0·5 mL sodium chloride solution (0·9%) as the placebo.

To record any immediate adverse events, participants were observed for at least 30 min after each vaccination. Participants recorded any solicited local and systemic adverse events using diary cards for 7 days following each vaccination. Clinical laboratory tests were done at 7 days after each vaccination, comprising a haematology panel (haemoglobin, haematocrit, red blood cell count, platelet count, and white blood cell count with differential) and a serum chemistry panel (alanine aminotransferase, aspartate aminotransferase, and creatinine) to check if there were any clinically relevant laboratory abnormalities that were reported as adverse events (appendix p 10). All participants received a 24-h telephone number to contact an on-call study physician in case of any medical problems. In stage 1, all adverse events were recorded from the first dose until 56 days after the second dose, and then again from the day of the booster vaccination until 28 days after the booster vaccination. In stage 2, adverse events were recorded for 28 days after each vaccination. In both stages 1 and 2, serious adverse events were recorded from the first dose until each participant’s last study visit (ie, up to 3 years after the first dose in stage 1, and up to 2 years after the first dose in stage 2). Further information on the grading of adverse events is presented in the appendix (pp 119, 143–148).

In stage 1, immunological assays were done on blood samples taken immediately before the first and second doses, then at 21 days after the second dose, 155 and 359 days after the first dose, and, thereafter, once every 6 months up to 3 years after the first dose. In participants who agreed to the booster vaccination, additional immunogenicity samples were collected immediately before the booster vaccination and at 4 days, 7 days, 21 days, 6 months, and 1 year after the booster vaccination. After initial results from the phase 1 studies^12–14^ were obtained, some timepoints were considered less relevant and the samples were not analysed. In stage 2, immunogenicity samples were collected immediately before the first dose, 28 days after the first dose, immediately before the second dose, 21 days and 6 months after the second dose, and 1 year and 2 years after the first dose.

Binding antibody responses against Ebola virus glycoprotein were analysed by use of the Ebola virus glycoprotein (Kikwit strain) Filovirus Animal Non-Clinical Group ELISA (validated by and done at Q^2^ Solutions Vaccine Testing Laboratory [San Juan Capistrano, CA, USA]) using the methods described in previous studies.^12–15^ In a randomly selected subset of stage 2 participants, the Ebola virus glycoprotein-specific neutralising antibody response was assessed by use of an Ebola virus glycoprotein (Makona strain) pseudovirion neutralisation assay, which was developed and validated by Monogram Biosciences (San Francisco, CA, USA), where this analysis was done (appendix pp 2). The presence of neutralising antibodies against the Ad26 and MVA vector backbones were measured at baseline by use of an Ad26-specific virus neutralisation assay, which was developed and qualified by Janssen Vaccines & Prevention BV, where this analysis was done, and a plaque reduction neutralisation test, which was developed and validated by Bavarian Nordic (Planegg, Germany), where this analysis was also done.

### Outcomes

For stage 1 and 2, the primary study outcome was to assess the safety of the Ad26.ZEBOV and MVA-BN-Filo vaccine regimen, defined as the occurrence of participants with solicited local and systemic adverse events in the first 7 days after each vaccination, unsolicited adverse events in the first 28 days after each vaccination, and serious adverse events or immediate reportable events up to the final study visit. The secondary outcomes were to assess Ebola virus glycoprotein-specific binding IgG antibody responses, as measured by ELISA at 21 days after the second dose in stage 1 and 2 participants; and to assess the safety and tolerability of the Ad26.ZEBOV booster vaccination administered at least 2 years after the first dose in stage 1 participants. Participants were considered as responders by ELISA if samples were negative at baseline and positive post-baseline with a value that was greater than 2·5 times the lower limit of quantification (LLOQ; 36·11 ELISA units [EU] per mL), or if a sample was positive both at baseline and post-baseline and there was a greater than 2·5-times increase from baseline.

The exploratory outcomes were to assess Ebola virus glycoprotein-specific binding antibody responses at other relevant timepoints (at 56, 155, 359, 539, and 719 days after the first dose, and at 4, 7, 21, and 359 days after the booster dose for stage 1; and at 56, 359 and 719 days after the first dose for stage 2) and to assess the neutralising activity of vaccine-induced antibody responses directed against Ebola virus glycoprotein and against the Ad26 and MVA vectors. Participants were considered as responders for the pseudovirion neutralisation assay if a sample was negative at baseline and positive post-baseline and the post-baseline value was greater than two times the LLOQ (a half maximal inhibitory concentration [IC50] titre of 120), or samples were positive both at baseline and post-baseline and there was a greater than two-times increase from baseline. Participants were considered as positive for the Ad26-specific virus neutralisation assay if a sample was greater than the LLOQ (a 90% inhibitory concentration titre of 17), and positive for the plaque reduction neutralisation test if the sample was greater than the LLOQ (an IC50 titre of 8). Only data from baseline samples are presented.

### Statistical analysis

The planned sample size for stage 1 (n=40) and stage 2 (n=400; 300 receiving Ad26.ZEBOV and MVA-BN-Filo, and 100 receiving MenACWY and placebo) were calculated to provide, when combined, a probability of 99% or higher of observing at least one serious adverse event occurring in at least 10% of participants in each group. The probability of observing at least one serious adverse event occurring in 1% of participants was 95% with a total sample size of 300 participants.

For the analysis of the Ebola virus glycoprotein-specific neutralising antibody response, a subset of 74 (28%) of 260 stage 2 participants were selected at random with SAS (version 9.2) in a 3:1 ratio of Ebola vaccine group participants to control group participants to ensure that the distribution of the selected participants was similar to the overall distribution of participants across the randomised groups in stage 2. The random selection was done before the sample analysis among 260 stage 2 participants with available samples and no protocol deviations that could have influenced the immune response. Ebola virus glycoprotein-specific neutralising antibody responses were not analysed in stage 1 participants. The selection of a subset of 74 participants for this analysis was not based on a separate sample size calculation, but was instead based on the number of samples that could be analysed in a reasonable amount of time, and was considered large enough to provide a representative characterisation of the neutralising antibody response. For the analysis of the neutralising antibodies against the Ad26 (with the virus neutralisation assay) and MVA (with the plaque reduction neutralisation test) vectors, all stage 1 participants and the subset of 74 stage 2 participants were included. We subsequently decided to analyse the neutralising antibody response against the Ad26 vector in all remaining stage 2 participants in the per-protocol analysis set who received the Ebola vaccine regimen.

Analysis of the primary outcome in stage 1 and stage 2 was done when all participants had completed the study or had discontinued early. The primary analysis set for safety (full analysis set) comprised all participants who had received at least one dose of study vaccine. Data are shown by vaccination group (as treated). The primary analysis set for immunogenicity (per-protocol) included all vaccinated participants who received both the first and second doses within the protocol-defined window, had at least one evaluable post-vaccination sample, and had no major protocol deviations that could have influenced the immune response. A sensitivity analysis was done in participants who received the second dose outside the protocol-defined window. Since the main purpose of stages 1 and 2 was to provide preliminary evaluation of safety and immunogenicity without formal hypothesis testing, all data were analysed by use of descriptive statistics.

Binding antibody responses against Ebola virus glycoprotein are shown as geometric mean concentrations (GMCs), and neutralising antibody activity is shown as geometric mean titres (GMTs), both with their associated 95% CIs. All values less than the LLOQ were imputed as half the LLOQ value. We calculated Spearman’s correlation coefficients to assess associations between Ebola virus glycoprotein-specific binding antibody concentrations and pseudovirion neutralisation assay titres at 21 days after the second dose. We did a post-hoc correlation analysis between Ad26 neutralising antibody titres before vaccination and Ebola virus glycoprotein-specific binding antibody responses at 21 days after the second dose. In addition, a post-hoc correlation analysis between Ebola virus glycoprotein-specific binding antibody concentrations measured at baseline and Ebola virus glycoprotein-specific binding antibody concentrations at 21 days after the second dose was done (appendix pp 3, 20).

All statistical analyses were done using SAS, version 9.2. This study is registered with ClinicalTrials. gov, NCT02509494.

### Role of the funding source

The Innovative Medicines Initiative 2 Joint Undertaking had no role in study design, data collection, data analysis, data interpretation, or writing of this report. Janssen Vaccines & Prevention BV had a role in study design, data collection, data analysis, data interpretation, and writing of the report.

## Results

**Figure 2:**
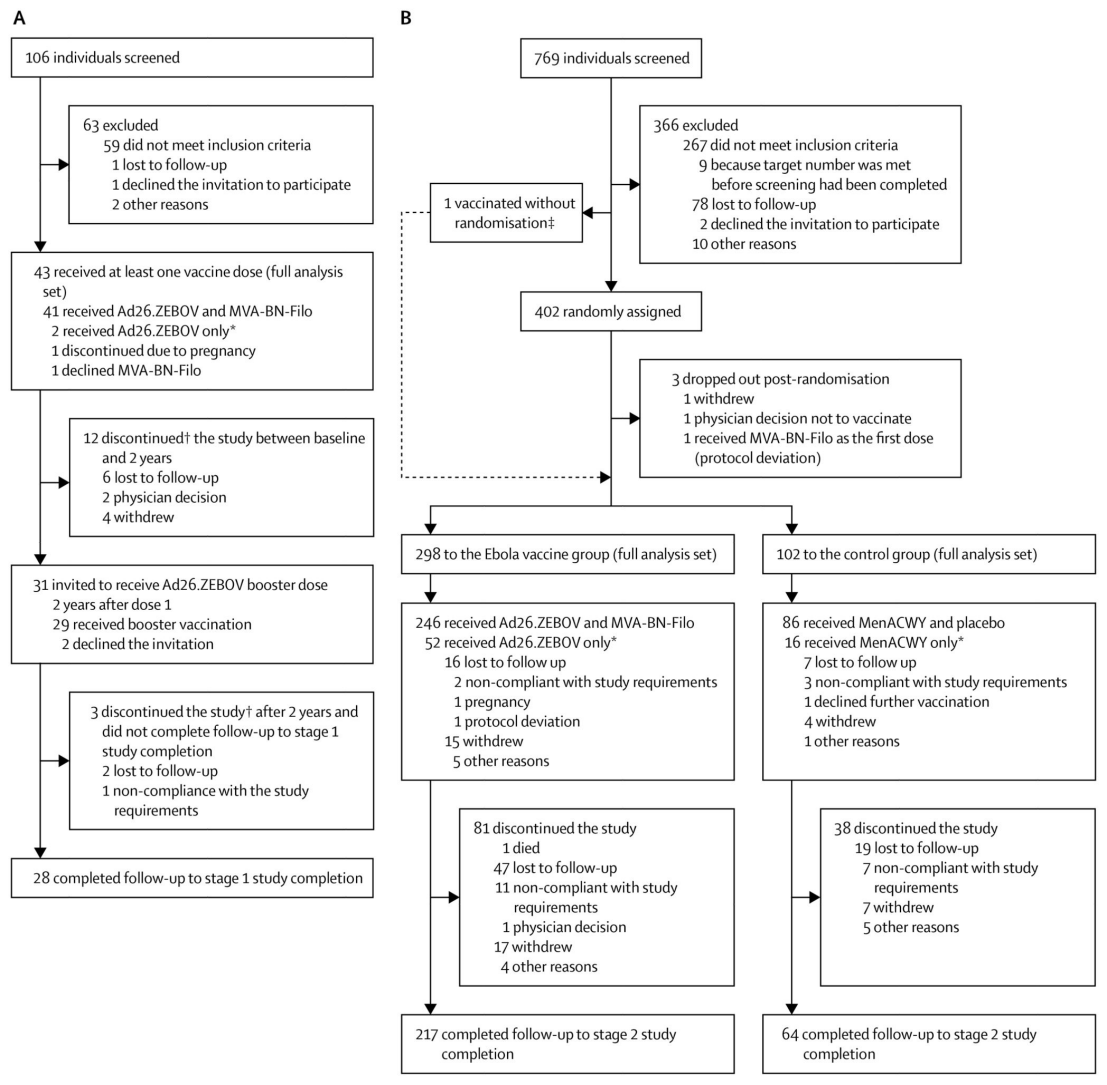
Stage 1 (A) and stage 2 (B) trial profiles Ad26.ZEBOV=adenovirus type 26 vector-based vaccine encoding the Ebola virus glycoprotein. MenACWY=meningococcal quadrivalent (serogroups A, C, W135, and Y) conjugate vaccine. MVA-BN-Filo=modified vaccinia Ankara vector-based vaccine, encoding glycoproteins from the Ebola virus, Sudan virus, and Marburg virus, and the nucleoprotein from the Tai Forest virus. * Participants did not receive the second vaccine irrespective of whether follow-up continued to study completion. †Follow-up did not continue to the end of the study, irrespective of the number of doses received. ‡This individual was properly screened and found to be eligible, but received the Ad26.ZEBOV vaccine before randomisation due to a protocol deviation.

Between Sept 30, 2015, and Oct 19, 2016, adult participants were recruited, and follow-up was completed on Nov 28, 2018. In stage 1, 106 individuals were screened, of whom 43 received at least the first dose of the Ad26.ZEBOV and MVA-BN-Filo vaccine regimen and were included in the full analysis set (figure 2A). Of 769 screened individuals in stage 2, 402 were randomly assigned and 400 received at least the first dose of the Ad26.ZEBOV and MVA-BN-Filo vaccine regimen (Ebola vaccine group; n=298) or the MenACWY and placebo regimen (control group; n=102; figure 2B) and were included in the full analysis set. The baseline characteristics of all participants are shown in table 1. 29 (94%) of 31 stage 1 participants invited to receive the booster vaccination received the booster at 2 years after the first dose.

**Figure 3:**
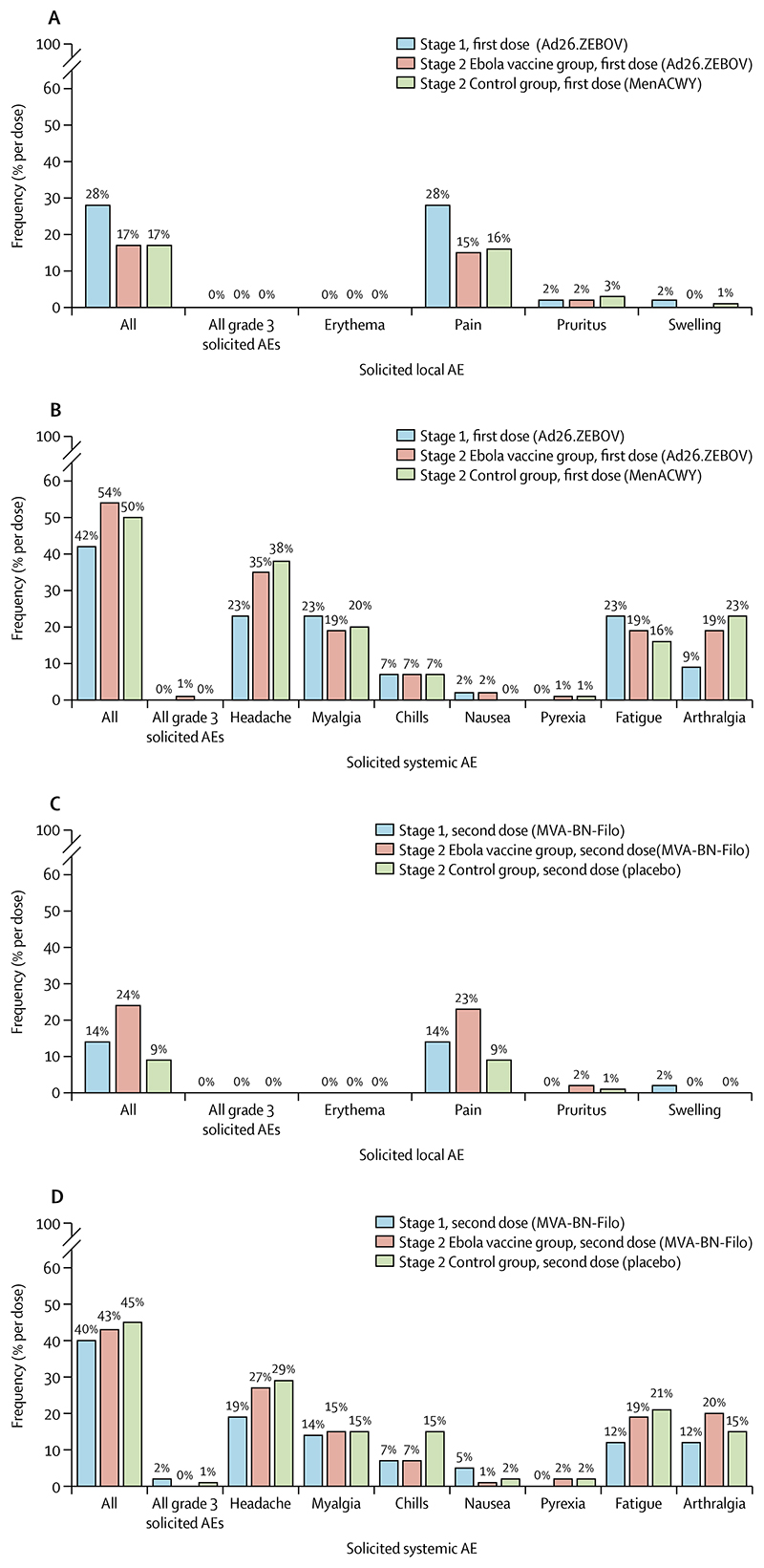
Solicited AEs after vaccination in stage 1 and stage 2 participants Solicited local (A) and systemic (B) AEs after the first dose, and solicited local (C) and systemic (D) AEs after the second dose. Solicited AEs were observed during the period of 7 days after vaccination. Grade 3 solicited AEs were severe AEs requiring medical attention, but which were not immediately life-threatening. Ad26.ZEBOV=adenovirus type 26 vector-based vaccine encoding the Ebola virus glycoprotein. MenACWY=meningococcal quadrivalent (serogroups A, C, W135, and Y) conjugate vaccine. MVA-BN-Filo=modified vaccinia Ankara vector-based vaccine, encoding glycoproteins from the Ebola virus, Sudan virus, and Marburg virus, and the nucleoprotein from the Tai Forest virus.

Solicited adverse events were mostly mild to moderate (grade 1 and 2) in severity (figure 3; appendix pp 4–8). In stage 1, at least one solicited local adverse event was reported in 12 (28%) of 43 participants after Ad26. ZEBOV vaccination and in six (14%) participants after MVA-BN-Filo vaccination (figure 3A, C; appendix pp 4–5). In stage 2, at least one solicited local adverse event was reported in 51 (17%) of 298 participants after Ad26.ZEBOV vaccination and in 58 (24%) of 246 participants after MVA-BN-Filo vaccination. In the control group, at least one solicited local adverse event was reported in 17 (17%) of 102 participants after MenACWY vaccination and in eight (9%) of 86 participants after placebo injection (figures 3A, C; appendix pp 4–5). The most frequent solicited local adverse event was injection-site pain after any vaccination (figure 3A, C; appendix pp 4–5). One (<1%) stage 2 participant had a grade 3 solicited local adverse event of injection-site pain after MVA-BN-Filo vaccination (figure 3C; appendix pp 4–5).

Solicited systemic adverse events in stage 1 were reported by 18 (42%) participants after Ad26.ZEBOV vaccination and by 17 (40%) after MVA-BN-Filo vaccination (figure 3B, D; appendix pp 6–8). In stage 2, at least one solicited systemic adverse event was reported in 161 (54%) participants after Ad26.ZEBOV vaccination, in 107 (43%) after MVA-BN-Filo vaccination, in 51 (50%) after MenACWY vaccination, and in 39 (45%) after placebo injection (figure 3B, 3D; appendix pp 6–8). Headache, myalgia, fatigue, and arthralgia were the most frequently reported solicited systemic adverse events after any vaccination, and grade 3 solicited systemic adverse events were infrequently observed (figure 3B, D; appendix pp 6–8).

In stage 1, unsolicited adverse events occurred in 17 (40%) of 43 participants after dose 1 and in 17 (40%) after dose 2. In stage 2, unsolicited events were reported in 198 (66%) of 298 participants after Ad26.ZEBOV vaccination, 145 (59%) of 246 after MVA-BN-Filo vaccination, 65 (64%) of 102 after MenACWY vaccination, and 48 (56%) of 86 after placebo injection (appendix p 9). The most frequent unsolicited adverse event after the first dose was headache in stage 1 and malaria in stage 2. Malaria was the most frequent unsolicited adverse event after the second dose in both stage 1 and 2 (appendix p 9). Grade 3 unsolicited adverse events were infrequent; observed in 2% of participants at most, regardless of vaccine received (appendix p 10).

At least one serious adverse event was reported in 23 (5%) of all 443 stage 1 and stage 2 participants during the study period (appendix pp 11–12); some participants had more than one serious adverse event. In 20 (87%) of 23 participants who reported a serious adverse event during the study period, the event occurred more than 28 days after vaccination, either with the first dose, the second dose, or the booster. In the 28-day period after the first dose, no stage 1 participants and two (<1%) of 298 stage 2 participants in the Ebola vaccine group reported at least one serious adverse event after Ad26. ZEBOV vaccination. One (1%) of 102 stage 2 participants in the control group reported at least one serious adverse event within 28 days of receiving the MenACWY vaccination. No serious adverse events were reported in stage 1 or stage 2 participants in the 28-day period after receiving the second dose. In addition, no stage 1 participants reported a serious adverse event in the 28-day period after receiving the booster dose. No reported serious adverse event was considered related to the study vaccine, and no immediate reportable events were observed. One death occurred in the Ebola vaccine group during the long-term follow-up period at day 198 after the second dose. This individual, who had a history of heavy alcohol consumption and use of unidentified traditional herbal medications, died due to severe dehydration caused by severe vomiting. The most commonly reported laboratory abnormality in stage 1 and 2 participants was a decrease in haemoglobin concentrations from baseline. Only two participants had haemoglobin concentrations less than the local laboratory range of normal, and no laboratory abnormalities were considered clinically significant by the investigator.

The post-booster vaccination adverse event profile in stage 1 participants who received the Ad26.ZEBOV booster vaccination at 2 years after the first dose was not notably different to that observed after the first dose (appendix pp 4–12).

**Figure 4:**
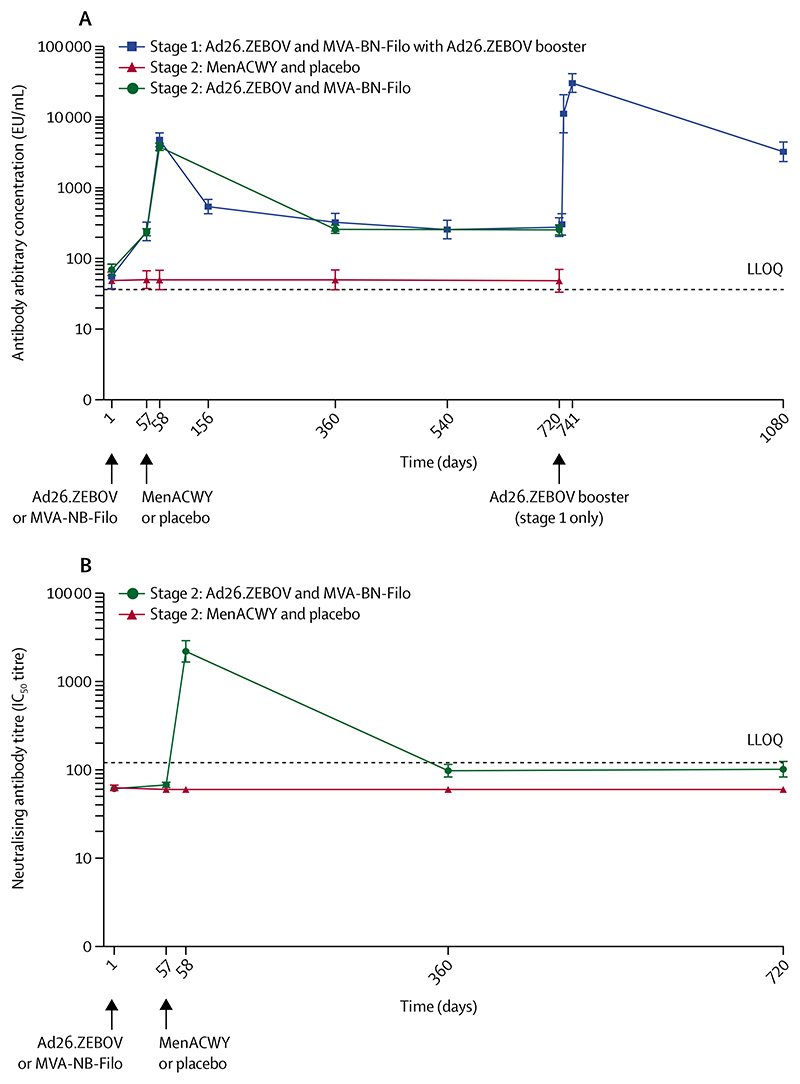
Ebola virus glycoprotein-specific binding antibody responses in stage 1 and 2 participants (A) and Ebola virus glycoprotein-specific neutralising antibody responses in stage 2 participants (B) In (A), the response profile for each study group is shown as geometric mean concentrations of anti-Ebola virus glycoprotein IgG. The error bars show the 95% CIs. Labels for day 724 (4 days after the booster vaccination) and day 727 (7 days after the booster vaccination) have been omitted. In (B), the response profile for each study group is shown as geometric mean titres. The error bars show the 95% CIs. Ad26.ZEBOV=adenovirus type 26 vector-based vaccine encoding the Ebola virus glycoprotein. EU=ELISA units. IC_50_=half maximal inhibitory concentration. LLOQ=lower limit of quantification. MenACWY=meningococcal quadrivalent (serogroups A, C, W135, and Y) conjugate vaccine. MVA-BN-Filo=modified vaccinia Ankara vector-based vaccine, encoding glycoproteins from the Ebola virus, Sudan virus, and Marburg virus, and the nucleoprotein from the Tai Forest virus.

All 43 stage 1 participants and 259 (65%) of 400 stage 2 participants (191 in the Ebola vaccine group and 68 in the control group) fulfilled the criteria for the per-protocol analysis set for the immunogenicity analyses. At 56 days after the first dose, Ebola virus glycoprotein-specific binding antibody responses were observed in 28 (65%) of 43 stage 1 participants (GMC 269 EU/mL [95% CI 208–347]) and 101 (54%) of 187 stage 2 participants (236 EU/mL [206–270]) in the Ebola vaccine group (table 2; figure 4A). At 21 days after the second dose, Ebola virus glycoprotein-specific binding antibody responses were observed in 41 (98%) of 42 stage 1 participants (4784 EU/mL [3736–6125]) and in 176 (98%) of 179 stage 2 participants (3810 EU/mL [3312–4383]).

Due to a study pause, which occurred between April 27 and June 16, 2016 for precautionary reasons during the evaluation of two serious adverse events following the administration of the same Ebola vaccine regimen in a different study,^15^ the second dose was delayed in 72 (18%) of 400 stage 2 participants (the time interval between the first and second doses ranged from 96 days to 147 days). This delayed administration of the second dose did not have a negative effect on Ebola virus glycoprotein-specific binding antibody responses. At 21 days after the second dose, antibody responses were observed in 44 (98%) of 45 stage 2 participants in the Ebola vaccine group who received the delayed second dose, with a GMC similar to that observed in participants who received the second dose within the protocol-defined window (delayed second dose GMC 5761 EU/mL [95% CI 3926–8455] *vs* second dose within protocol-defined window 3823 EU/mL [3334–4383]; appendix pp 13–14).

At day 156 (3 months after the second dose), the magnitude of Ebola virus glycoprotein-specific binding antibody concentrations in stage 1 participants had decreased compared with 21 days after the second dose, with a GMC of 544 EU/mL (95% CI 422–701), and remained largely stable until day 720 (table 2; figure 4A). At day 360, persistent Ebola virus glycoprotein-specific binding antibody responses were observed in 24 (77%) of 31 stage 1 participants (GMC 325 EU/mL [95% CI 238–445]) and in 82 (49%) of 166 stage 2 participants (259 EU/mL [223–301]). At day 720, a persistent antibody response was observed in 21 (68%) of 31 stage 1 participants (279 EU/mL [201–386]) and in 78 (50%) of 155 stage 2 participants (255 EU/mL [212–306]).

At 7 days after the Ad26.ZEBOV booster vaccination, given to a subset of 29 stage 1 participants 2 years after the first dose, 24 (96%) of 25 showed a strong increase in Ebola virus glycoprotein-specific binding antibody concentrations, with a GMC of 11166 EU/mL (95% CI 5881–21 201), which is 40-times higher than the GMC at the pre-booster vaccination timepoint (279 [95% CI 201–386]). At 21 days after the booster vaccination, all 29 participants had an Ebola virus glycoprotein-specific binding antibody response, with a GMC of 30 411 EU/mL (21 972–42 091), which was approximately 110-times higher than the pre-booster vaccination GMC (table 2; figure 4A) and six-times higher than the GMC at 21 days after the second dose. Ebola virus glycoprotein-specific binding antibody concentrations decreased at 1 year after the booster vaccination, with a GMC of 3237 EU/mL (2305–4547); however, persistent responses were observed in all 26 participants still on follow-up at this timepoint, at a level that was at least nine-times higher than that observed at 1 year and 2 years after the first dose.

Ebola virus glycoprotein-specific neutralising antibody titres were measured in a randomly selected subset of 74 stage 2 participants (55 [18%] of 298 in the Ebola vaccine group and 19 [19%] of 102 in the control group; figure 4B; appendix pp 15–16). At 56 days after the first dose, an Ebola virus glycoprotein-specific neutralising antibody response was observed in one (2%) of 51 participants in the Ebola vaccine group, with a GMT less than the LLOQ. At 21 days after the second dose, an Ebola virus glycoprotein-specific neutralising antibody response was observed in 52 (98%) of 53 participants in the Ebola vaccine group, with a GMT of 2199 (95% CI 1634–2960). There was a strong positive correlation between Ebola glycoprotein-specific binding antibody concentrations and neutralising antibody titres at 21 days after the second dose in participants who received both doses of the Ebola vaccine regimen (*r*=0·751; appendix p 19). At day 360, the neutralising antibody response persisted in three (6%) of 53 participants in the Ebola vaccine group. At approximately 2 years after the first dose, neutralising antibody responses were observed in six (12%) of 51 participants in the Ebola vaccine group.

Pre-existing Ad26-specific neutralising antibody titres were measured in all 43 stage 1 participants, and in 209 (52%) of 400 stage 2 participants (191 [64%] of 298 in the Ebola vaccine group and 18 [18%] of 102 in the control group). Pre-existing Ad26-specific neutralising antibodies were detected in 40 (93%) stage 1 participants, in 177 (93%) stage 2 participants in the Ebola vaccine group, and in 17 (94%) stage 2 participants in the control group, with similar GMTs observed among the three groups (90% inhibitory concentration GMTs of 111 [95% CI 75–163] in stage 1 participants, 124 [101–151] in stage 2 participants in the Ebola vaccine group, and 104 [57–190] in stage 2 participants in the control group; appendix p 16). There was no correlation between baseline Ad26-specific neutralising antibody titres and vaccine-induced Ebola virus glycoprotein-specific binding antibody concentrations at 21 days after the second dose (*r*=–0·145; appendix p 19).

Before vaccination, MVA-specific neutralising antibodies were analysed in almost all stage 1 participants (42 [98%] of 43) and in 74 (19%) of 400 stage 2 participants [56 [19%] of 298 in the Ebola vaccine group and 18 (18%) of 102 in the control group). Neutralising antibodies against the MVA vector were observed in only two (5%) stage 1 participants, in three (5%) stage 2 participants in the Ebola vaccine group, and in three (17%) stage 2 participants in the control group. The GMT values for all three groups of participants at baseline were all less than the LLOQ (appendix p 17).

## Discussion

To our knowledge, this is the first clinical study to assess the safety and tolerability two-dose heterologous Ad26. ZEBOV and MVA-BN-Filo vaccine regimen in a region affected by the west African Ebola virus disease outbreak in 2014–16. The results showed that this regimen was well tolerated; injection-site pain was the most frequent solicited local adverse event, and headache, myalgia, fatigue, and arthralgia were the most frequent solicited systemic adverse events. No serious adverse events were considered related to the study vaccine.

The Ad26.ZEBOV and MVA-BN-Filo vaccine regimen induced Ebola virus glycoprotein-specific binding and neutralising antibody responses in 98% of participants at 21 days after the second dose. At this timepoint, a strong positive correlation was observed between binding antibody concentrations and neutralising antibody titres. The magnitude of antibody responses declined over time, although Ebola virus glycoprotein-specific binding antibody responses persisted in 24 (77%) of 31 stage 1 participants and in 82 (49%) of 166 stage 2 participants in the Ebola vaccine group at 1 year after the first dose and in 21 (68%) of 31 stage 1 participants and 78 (50%) of 155 stage 2 participants at 2 years after the first dose. In a randomly selected subset of stage 2 participants in the Ebola vaccine group, neutralising antibody responses persisted in three (6%) of 53 participants at 1 year after the first dose and in six (12%) of 51 participants at 2 years after the first dose.

Although more than 90% of participants had pre-existing neutralising antibodies specific for the Ad26 vector, correlation analyses indicated that there was no association between pre-existing Ad26-specific immunity and the vaccine-induced Ebola virus glycoprotein-specific binding antibody responses (appendix p 19).

The immunogenicity findings described in this report are consistent with data observed in previous studies showing the safety and immunogenicity of the Ad26. ZEBOV and MVA-BN-Filo vaccine regimen in a European population^13,15,20^ and in east African populations that were not affected by the 2014–16 Ebola outbreak.^12,14^ The kinetics of the humoral responses observed in phase 1 and 2 clinical studies are supported by the results of our study.^12–15,20^

A small proportion (18%) of stage 2 participants received their second dose outside the protocol-defined window. A sensitivity analysis showed that extension of the time interval between Ad26.ZEBOV and MVA-BN-Filo doses did not have a negative effect on vaccine-induced immune responses at 21 days after the second dose, as 44 (98%) of 45 participants who received a delayed second dose had Ebola virus glycoprotein-specific binding antibody responses, with a GMC similar to that observed in participants who received the second dose within the protocol-defined window. Our study also showed that a booster vaccination with Ad26.ZEBOV given at 2 years after the first dose was well tolerated and induced a strong anamnestic response, as evidenced by Ebola virus glycoprotein-specific binding antibody concentrations that were approximately 40-times higher at 7 days after the booster vaccination and approximately 110-times higher at 21 days after the booster vaccination than immediately before the booster. Ebola virus glycoprotein-specific binding antibody concentrations decreased at 1 year after the booster vaccination; however, responses were observed in all participants at that timepoint, at a concentration that was at least nine-times higher than that observed at 1 year and 2 years after the first dose. This finding indicates that the Ad26.ZEBOV and MVA-BN-Filo vaccine regimen had induced humoral immune memory, which we believe can be triggered by future natural infections and is important for subsequent considerations of the deployment of this vaccine. Prophylactic vaccination with the Ad26.ZEBOV and MVA-BN-Filo vaccine regimen could be considered as a medium-term to long-term strategy. In addition, as a precautionary measure, a booster vaccination with Ad26.ZEBOV could be considered in anticipation of an imminent exposure to Ebola virus.

This study has some limitations, including the imbalance in the numbers of men and women in the study population (most participants were men because of local socioeconomic and cultural factors); the exclusion of pregnant women, as is generally conventional during trials of new investigational products (with the related requirement for contraception in those of childbearing potential);^21^ the measurement of Ebola virus glycoprotein-specific neutralising antibody titres in only a subset of participants; and the offer of a booster dose to only stage 1 participants. The study was initially planned as a large cluster-randomised trial, with vaccine effectiveness as the primary endpoint; however, the study design and outcomes were changed after the Ebola virus disease outbreak in Sierra Leone declined (ie, the cluster-randomised trial component was removed, the follow-up period was extended, and the booster dose in stage 1 participants was included). The addition of the booster dose was to ascertain whether the Ad26.ZEBOV and MVA-BN-Filo vaccine regimen could establish a memory response. As stage 1 participants were the first to be vaccinated in the study, they were also the first group to reach the follow-up timepoint of 2 years after the first dose, when the booster dose was due to be administered and when the collection of data at 1 year after the booster dose in this group would not have delayed the reporting of the results of the overall study.

Aside from these limitations, the study has many strengths, including the enrolment of participants in a country previously affected by Ebola virus; a 2-year follow-up period, which provided the opportunity to assess the durability of immune responses; and the inclusion of a booster vaccination given 2 years after the initial vaccination. Starting the study during the Ebola outbreak, in a largely rural setting, and in a research-naive population, has provided valuable lessons regarding clinical trial implementation and conduct under difficult conditions.^18^ Participant retention was challenging, especially in the aftermath of the outbreak, as some individuals relocated outside the study area for work, business, or study. Despite this challenge, reasonable long-term retention rates were achieved due to concerted community trust-building and participant follow-up arrangements.^17,18,22^

The Ad26.ZEBOV and MVA-BN-Filo vaccine regimen, with a 56-day interval between the two doses, assessed in this study, received marketing authorisation on July 1, 2020, for prophylactic use, under exceptional circumstances, in adults and children aged 1 year or older in the EU.^10^ This vaccine regimen was previously shown to provide protection in vaccinated non-human primates against an Ebola virus challenge, which is fully lethal in unvaccinated control animals.^11^ In the absence of clinical efficacy data in humans, a statistical approach referred to as immunobridging using data from this study and other clinical studies was used to infer the likelihood of protection induced by vaccination by correlating the magnitude of vaccine-elicited immune parameters in non-human primates with those observed in vaccinated humans.^23^ Although a mechanistic correlate of protection has not yet been identified, the Ebola virus glycoprotein-specific binding antibody GMCs observed 21 days after the second dose in participants who received the Ad26. ZEBOV and MVA-BN-Filo vaccine regimen were similar to the GMC of 1262 EU/mL (95% CI 1169–1363) reported at 28 days after rVSV-ZEBOV-GP vaccination by use of the same assay in the same laboratory.^24^ rVSV-ZEBOV-GP, which was the first Ebola virus vaccine to receive conditional marketing authorisation in Europe and approval for use in adults in the USA and several African countries,^7–9^ is the only vaccine for which data on vaccine effectiveness are currently available (ie, estimated vaccine effectiveness of 100% from 10 days after vaccination onwards in a phase 3 trial in Guinea during the west African outbreak,^4^ and an estimated vaccine effectiveness of 97·5% in DR Congo).^6^

Recognising the threat of unpredictable future Ebola virus disease outbreaks, further vaccine development work is crucial to strengthen international health security by diversifying vaccination strategy options. Additional studies are in progress, such as PREVAC (NCT02876328), a randomised trial currently underway in Sierra Leone, Guinea, Liberia, and Mali assessing three vaccine strategies in adults and children, including the Ad26. ZEBOV and MVA-BN-Filo vaccine regimen, the single-dose rVSV-ZEBOV-GP vaccine, and a two-dose rVSV-ZEBOV-GP vaccine regimen.^25^ Another study, DRC-EB-001 (NCT04152486), is currently underway in North Kivu, DR Congo, to assess the feasibility and safety of the two-dose Ad26.ZEBOV and MVA-BN-Filo regimen at the population level. EBL2007 (NCT0418600) in DR Congo and EBL2009 (NCT04028349) in Uganda are two ongoing open-label trials that will provide additional information on the immunogenicity and safety of the Ad26.ZEBOV and MVA-BN-Filo vaccine regimen.

In conclusion, our findings show that in healthy African adult volunteers living in a region previously affected by Ebola virus disease, the Ad26.ZEBOV and MVA-BN-Filo vaccine regimen administered with a 56-day interval between the two doses is well tolerated and induces humoral immune responses that persist for at least 2 years, as well as humoral immune memory. Booster vaccination with Ad26.ZEBOV administered 2 years after the first dose induces a strong anamnestic response within 7 days, which could be valuable for populations at imminent risk of exposure to Ebola virus, such as health workers in Ebola-endemic settings.

## Supplementary Material

Supplementary appendix

## Figures and Tables

**Table 1:** Participant demographic and baseline characteristics

	Stage 1 (n=43)	Stage 2	
		Ad26.ZEBOV and MVA-BN-Filo Ebola vaccine group (n=298)	MenACWY and placebo control group (n=102)
Sex			
Female	1 (2%)	50 (17%)	22 (22%)
Male	42 (98%)	248 (83%)	80 (78%)
Age at screening, years	23 (20-27)	23 (21-31)	25 (21-35)
Height, cm	170 (167-173)	169 (163-173)	166 (162-173)
Weight, kg	63 (58-68)	62 (56-67)	61 (56-67)
Body-mass index, kg/m^2^	22(21-23)	22 (20-23)	22 (20-23)

Data are n (%) or median (IQR). Participants in stage 1 were assigned to receive Ad26.ZEBOV, followed by MVA-BN-Filo 56 days later; a subset of these participants received a booster of Ad26.ZEBOV 2 years after the first dose. Ad26. ZEBOV=adenovirus type 26 vector-based vaccine encoding the Ebola virus glycoprotein. MVA-BN-Filo=modified vaccinia Ankara vector-based vaccine, encoding glycoproteins from the Ebola virus, Sudan virus, and Marburg virus, and the nucleoprotein from the Tai Forest virus. MenACWY=meningococcal quadrivalent (serogroups A, C, W135, and Y) conjugate vaccine.

**Table 2:** Ebola glycoprotein-specific binding antibody responses in each study group from baseline to study completion

	Stage 1 (Ad26.ZEBOV and MVA-BN-Filo with an Ad26.ZEBOV booster at 2 years after dose 1)	Stage 2	
		Ad26.ZEBOV and MVA-BN-Filo Ebola vaccine group	MenACWY and placebo control group
**Day 1 (baseline)**			
Number of participants*	43	188	66
GMC (95% CI), EU/mL	60 (40–90)	69 (56–85)	49 (36–66)
**Day 57 (56 days after the first dose)**			
Number of participants*	43	190	68
GMC (95% CI), EU/mL	269 (208–347)	236 (206–270)	50 (37–69)
Responders†	28/43 (65%; 49–79)	101/187 (54%; 47–61)	4/66 (6%; 2–15)
**Day 78 (21 days after the second dose)**			
Number of participants*	42	182	62
GMC (95% CI), EU/mL	4784 (3736–6125)	3810 (3312–4383)	50 (<LLOQ–70)
Responders†	41/42 (98%; 87–100)	176/179 (98%; 95–100)	2/60 (3%; 0–5)
**Day 156 (155 days after the first dose)**			
Number of participants*	41	..	..
GMC (95% CI), EU/mL	544 (422–701)	..	..
Responders†	32/41 (78%; 62–89)	..	..
**Day 360 (359 days after the first dose)**			
Number of participants*	31	168	62
GMC (95% CI), EU/mL	325 (238–445)	259 (223–301)	50 (<LLOQ–71)
Responders†	24/31 (77%; 59–90)	82/166 (49%; 42–57)	4/60 (7%; 2–16)
**Day 540 (539 days after the first dose)**			
Number of participants*	32	..	..
GMC (95% CI), EU/mL	257 (186–356)	..	..
Responders†	23/32 (72%; 53–86)	..	..
**Day 720 (719 days after the first dose)**			
Number of participants*	31	158	48
GMC (95% CI), EU/mL	279 (201–386)	255 (212–306)	49 (<LLOQ–72)
Responders†	21/31 (68%; 49–83)	78/155 (50%; 42–58)	7/47 (15%; 6–28)
**Day 724 (4 days after booster vaccination)**			
Number of participants*	27	NA	NA
GMC (95% CI), EU/mL	304 (211–440)	NA	NA
Responders†	19/27 (70%; 50–86)	NA	NA
**Day 727 (7 days after booster vaccination)**			
Number of participants*	25	NA	NA
GMC (95% CI), EU/mL	11 166 (5881–21 201)	NA	NA
Responders†	24/25 (96%; 80–100)	NA	NA
**Day 741 (21 days after booster vaccination)**			
Number of participants*	29	NA	NA
GMC (95% CI), EU/mL	30 411 (21 972–42 091)	NA	NA
Responders†	29/29 (100%; 88–100)	NA	NA
**Day 1080 (359 days after booster vaccination)**			
Number of participants*	26	NA	NA
GMC (95% CI), EU/mL	3237 (2305–4547)	NA	NA
Responders†	26/26 (100%; 87–100)	NA	NA

For the proportions of responders, exact (Clopper-Pearson) 95% CIs are shown. A participant was considered a responder at a specific timepoint if either: (1) the sample was negative at baseline and positive post-baseline, and the post-baseline value was more than 2·5-times higher than the LLOQ; or (2) the sample was positive both at baseline and post-baseline, and there was a greater than 2·5-times increase from baseline. Ad26.ZEBOV=adenovirus type 26 vector-based vaccine encoding the Ebola virus glycoprotein. EU=ELISA units. GMC=geometric mean concentration. LLOQ=lower limit of quantification. MenACWY=meningococcal quadrivalent (serogroups A, C, W135, and Y) conjugate vaccine. MVA-BN-Filo=modified vaccinia Ankara vector-based vaccine, encoding glycoproteins from the Ebola virus, Sudan virus, and Marburg virus, and the nucleoprotein from the Tai Forest virus. NA=not appropriate. *Refers to the number with data at that timepoint. †Expressed as n/N (%; 95% CI), where n is the number of responders at that timepoint and N is the total number of participants with data at baseline and at that timepoint.

## Data Availability

Janssen has an agreement with the Yale Open Data Access (YODA) Project to serve as the independent review panel for evaluation of requests for clinical study reports and participant-level data from investigators and physicians for scientific research that will advance medical knowledge and public health. Data will be made available following publication and approval by YODA of any formal requests with a defined analysis plan. For more information on this process, or to make a request, please visit The YODA Project website at http://yoda.yale.edu. The data sharing policy of Janssen is available at https://www.janssen.com/clinical-trials/transparency. We consider that the study methods and results in adult participants are clearly documented in this Article. Study methods for enrolment of children and their results will be presented in a separate publication. The clinical study protocol is available in the appendix (pp 25–154).

## Research in context

### Evidence before this study

We searched PubMed on Feb 20, 2020, using the search terms “ebola” AND “vaccin* OR immunis* OR immuniz*” AND “trial* OR study”. We searched for articles published between database inception up to Feb 20, 2020, with no language restrictions. We identified 733 articles. After screening the titles and abstracts, we identified 40 publications reporting on the immunogenicity or safety, or both, of Ebola vaccine candidates across 34 clinical trials. We also consulted a WHO overview of candidate Ebola virus vaccines dated Aug 19, 2019. Several vaccine candidates have been tested in phase 1 and 2 clinical trials (eg, the recombinant vesicular stomatitis virus-vectored vaccine expressing the glycoprotein of Ebola virus [rVSV-ZEBOV-GP]; the recombinant chimpanzee adenovirus type 3 virus-vectored vaccine encoding the glycoprotein of Ebola virus [ChAd3-EBO-Z]; the recombinant adenovirus type-5 vectored vaccine encoding the glycoprotein of Ebola virus [Ad5-EBOV]; and a vaccine consisting of rVSV and Ad5 encoding the glycoprotein of Ebola virus [GamEvac Combi]), with acceptable safety profiles and promising immunogenicity results. Data on the effectiveness against Ebola virus disease were available for only the rVSV-ZEBOV-GP vaccine, with an estimated effectiveness of 100% in a ring vaccination trial done in Guinea during the 2014-16 outbreak, and of 97·5% in a ring vaccination strategy to control the 2018-20 Ebola virus disease outbreak in the Democratic Republic of the Congo. The two-dose heterologous vaccination regimen with a replication deficient adenovirus type 26 vector-based vaccine encoding the Ebola virus glycoprotein (Ad26.ZEBOV), and a non-replicating, recombinant, modified vaccinia Ankara vector-based vaccine, encoding glycoproteins from the Ebola virus, Sudan virus, and Marburg virus, and the nucleoprotein from the Tai Forest virus (MVA-BN-Filo) was shown to have a good safety profile in three phase 1 studies in European and healthy African individuals living in areas unaffected by the Ebola virus. The most common adverse events were pain at the injection site and headache. No vaccine-related serious adverse events were reported. The Ad26.ZEBOV and MVA-BN-Filo vaccine regimen induced durable immune responses for at least 1 year in healthy adults.

### Added value of this study

To our knowledge, this is the first study to provide data on the safety, long-term immunogenicity, and humoral immune memory responses induced by the Ad26.ZEBOV and MVA-BN-Filo Ebola vaccine regimen in healthy adults from a population severely affected by the 2014-16 Ebola virus disease epidemic in west Africa. The vaccine regimen was well tolerated and induced humoral immune responses that persisted for at least 2 years. Booster vaccination with Ad26.ZEBOV, given 2 years after the initial vaccination, induced a strong anamnestic response within 7 days. These findings will inform the future use of this vaccine regimen; for instance, by justifying the strategy of providing a booster to previously immunised individuals at the start of an Ebola virus disease outbreak. Our findings also supported the decision of the European Commission to grant marketing authorisations for the Ad26.ZEBOV and MVA-BN-Filo Ebola vaccine regimen in the EU.

### Implications of all the available evidence

Several vaccines against Ebola virus disease have been shown to be safe and immunogenic in clinical trials. One vaccine, rVSV-ZEBOV-GP, has also been shown to be highly effective in preventing Ebola virus disease. Vaccine research should continue to ascertain the long-term immunogenicity of these vaccines, and to assess different options for prophylactic vaccination in populations at potential risk of Ebola virus disease or for reactive vaccination during Ebola virus disease outbreaks.
