# Supplementary material for: Safety and long-term immunogenicity of the two-dose heterologous Ad26.ZEBOV and MVA-BN-Filo Ebola vaccine regimen in adults in Sierra Leone: a combined open-label, non-randomised stage 1, and a randomised, double-blind, controlled stage 2 trial
Source: Lancet Infect Dis. Author manuscript; Available in PMC 2022 Sep 7. (PMC7613326; doi:10.1016/S1473-3099(21)00125-0)
Supplement: Supplementary appendix [file EMS152543-supplement-Supplementary_appendix.pdf]

THE LANCET
Infectious Diseases

Supplementary appendix

This appendix formed part of the original submission and has been peer reviewed. We post it as supplied by the authors.

Supplement to: Ishola D, Manno D, Afolabi MO, et al. Safety and long-term immunogenicity of the two-dose heterologous Ad26.ZEBOV and MVA-BN-Filo Ebola vaccine regimen in adults in Sierra Leone: a combined open-label, non-randomised stage 1, and a randomised, double-blind, controlled stage 2 trial. *Lancet Infect Dis* 2021; published online Sept 13. https://doi.org/10.1016/S1473-3099(21)00125-0.

Safety and long-term immunogenicity of a two-dose Ad26.ZEBOV, MVA-BN-Filo Ebola vaccine regimen in adults: a randomised, double-blind, controlled trial in Sierra Leone

Supplementary material

Table of contents

1. SUPPLEMENTARY METHODS 2
1.1. Data management 2
1.2. Determination of neutralising antibody activity in an EBOV GP pseudovirion neutralisation assay 2

2. SUPPLEMENTARY RESULTS 3
2.1. *Table S1*: Solicited adverse events: Solicited local adverse events by worst severity grade 4
2.2. *Table S2*: Solicited adverse events: Solicited systemic adverse events by worst severity grade. 6
2.3. *Table S3*: Unsolicited adverse events: Most frequent (at least 10% of participants in any vaccination schedule) unsolicited adverse events by system organ class and dictionary-derived term. 9
2.4. *Table S4*: Grade 3 unsolicited adverse events reported after each dose (up to 28 days post dose) 10
2.5. *Table S5*: Serious adverse events reported during the study. 11
2.6. *Table S6*: Ebola glycoprotein-specific binding antibody, stratified by the timing of dose 2 vaccination (dose 2 vaccination received at the protocol planned time, or delayed). 13
2.7. *Table S7*: Ebola glycoprotein-specific neutralising antibody concentrations, as measured in a subset of Stage 2 participants 15
2.8. *Table S8*: Ad26 neutralizing antibodies (Ad26 VNA, IC_{90} titre): Geometric mean and sample interpretation: per protocol analysis set. 16
2.9. *Table S9*: MVA neutralizing antibodies (MVA PRNT, IC_{50} titre): Geometric mean and sample interpretation: per protocol analysis set. 17
2.10. *Figure S1*: Correlation between Ebola glycoprotein-specific binding antibody concentrations and Ebola glycoprotein-specific neutralizing antibody titres at 21 days post dose 2 in participants who received Ebola vaccine regimen 18
2.11. *Figure S2*: Correlation between baseline Ad26-specific nAb titres and the vaccine-induced EBOV GP-specific binding antibody concentrations at 21 days post-dose 2 19
2.12. *Figure S3*: Correlation between pre-existing Ebola glycoprotein-binding antibody at baseline and at 21 days post dose 2 in participants who received Ebola vaccine regimen 20

3. EBL3001 STUDY GROUP 21

4. STUDY PROTOCOL 25

1. SUPPLEMENTARY METHODS

1.1. Data management

Paper source document data were entered onto electronic data collection forms in Medidata Rave Clinical Cloud™. Data were subjected to visual (ie, completeness of source documents) and electronic validation to check for discrepancies, missing data and protocol deviations in the database. Issues were notified to the site as queries for resolution. Data were source-verified, and all queries were resolved before data lock for interim and final analyses.

1.2. Determination of neutralising antibody activity in an Ebola Virus Glycoprotein (EBOV GP) pseudovirion neutralisation assay

In order to assess the functionality of vaccine-induced antibody responses, a pseudovirion neutralisation assay (psVNA) was developed at Monogram Biosciences (San Francisco, CA, USA) and was qualified with human serum. Samples for the current report were assayed at Monogram Biosciences according to the standard operating procedure ‘Crucell EBOV Neutralization Assay’.

Pseudovirions expressing the glycoprotein of an EBOV isolate from the 2014 outbreak (Makona variant) were produced in human embryonic kidney 293 (HEK293) cells by transfection with a GP expression plasmid and a vector encoding a firefly luciferase gene and all of the human immunodeficiency virus (HIV) type 1 genes required for viral replication, except for the envelope gene. Sera were pretreated to remove any nonspecific neutralising factors. A fixed amount of pseudovirions was mixed with a series of serial dilutions of serum samples. Following incubation, the samples were transferred to a HEK293 cell monolayer. The inhibition of pseudovirion infection was measured by luciferase reporter gene expression. The assay responses of the serially diluted samples were plotted in a 4-parameter logistic regression curve and the 50% inhibitory concentration (IC_{50}) of each curve was reported as each a neutralisation titre for each serum sample.

A psVNA result (IC_{50} titre) was considered positive if the specific IC_{50} titre was more than three times amphotropic murine leukaemia virus (aMLV) and above the assay-specific lower limit of quantitation (LLOQ). Values that were less than three times aMLV or below the LLOQ were imputed with LLOQ/2 (120/2). For the calculation of fold increases, values that were less than three times aMLV or below the LLOQ were imputed with the LLOQ. The psVNA values were $\log_{10}$-transformed before further handling. The $\log_{10}$-transformed values were used throughout the entire analysis.

For psVNA, a participant was a responder at a considered time point if the sample interpretation was negative at baseline and positive post baseline and the post-baseline value was greater than twice the LLOQ, or sample interpretation was positive both at baseline and post baseline and there was a greater than two-fold increase from baseline.

2. SUPPLEMENTARY RESULTS

In 142 participants who received the Ad26.ZEBOV, MVA-BN-Filo vaccine regimen (25/43 [58%] participants in Stage 1 and 117/188 [62%] participants in Stage 2), the baseline sample interpretation was positive for EBOV GP-specific binding antibodies (ie, the sample value was above the LLOQ). Yet, the GMC values at baseline were low (60 ELISA units/mL in Stage 1 and 69 ELISA units/mL in Stage 2). To evaluate the potential impact of EBOV GP-specific binding antibody concentrations detected by ELISA at baseline on the 21 days post dose 2 ELISA values, a correlation analysis was performed (Figure S3). If baseline ELISA concentrations were an indication of priming of the immune system triggered by previous natural Ebola virus infection, one would expect a positive (anamnestic response) or a negative (immune interference) correlation between the baseline and the post-dose 2 ELISA concentrations. However, a negligible positive correlation was observed, indicating that there is no apparent influence of the observed baseline EBOV GP FANG ELISA positivity on the binding antibody concentrations following Ad26.ZEBOV, MVA-BN-Filo vaccination (Spearman correlation coefficient: 0·104).

2.1. *Table S1*: Solicited adverse events: Solicited local adverse events by worst severity grade

		Stage 1	Stage 2	
**Post dose 1**		**Ad26.ZEBOV**	**Ad26.ZEBOV**	**MenACWY**
**n**		**43**	**298**	**102**
Any solicited local event, n (%)	Any	12 (28)	51 (17)	17 (17)
	Grade 1	11 (26)	50 (17)	14 (14)
	Grade 2	1 (2)	1 (0)	3 (3)
Injection site erythema	Any	0	1 (0)	0
	Grade 2	0	1 (0)	0
Injection site pain	Any	12 (28)	44 (15)	16 (16)
	Grade 1	11 (26)	44 (15)	13 (13)
	Grade 2	1 (2)	0	3 (3)
Injection site pruritus	Any	1 (2)	6 (2)	3 (3)
	Grade 1	1 (2)	6 (2)	2 (2)
	Grade 2	0	0	1 (1)
Injection site swelling	Any	1 (2)	1 (0)	1 (1)
	Grade 1	1 (2)	1 (0)	0
	Grade 2	0	0	1 (1)
**Post dose 2**		**MVA-BN-Filo**	**MVA-BN-Filo**	**Placebo**
**n**		**43**	**246**	**86**
Any solicited local event, n (%)	Any	6 (14)	58 (24)	8 (9)
	Grade 1	6 (14)	47 (19)	8 (9)
	Grade 2	0	10 (4)	0
	Grade 3	0	1 (0)	0
Injection site erythema	Any	0	0	0
Injection site pain	Any	6 (14)	57 (23)	8 (9)
	Grade 1	6 (14)	47 (19)	8 (9)
	Grade 2	0	9 (4)	0
	Grade 3	0	1 (0)	0
Injection site pruritus	Any	0	5 (2)	1 (1)
	Grade 1	0	4 (2)	1 (1)
	Grade 2	0	1 (0)	0
Injection site swelling	Any	1 (2)	0	0
	Grade 1	1 (2)	0	0
**Post booster dose**		**Ad26.ZEBOV**		
**n**		**29**	–	–
Any solicited local event, n (%)	Any	5 (17)		
	Grade 1	5 (17)		
Injection site erythema	Any	0		
Injection site pain	Any	5 (17)		
	Grade 1	5 (17)		
Injection site pruritus	Any	1 (3)		
	Grade 1	1 (3)		
Injection site swelling	Any	0		

Vaccines: Ad26.ZEBOV; MVA-BN-Filo. Control: Meningococcal quadrivalent (serogroups A, C, W135 and Y) conjugate vaccine (MenACWY; dose 1), Placebo (dose 2). n=number of participants with data.

2.2. ***Table S2*: Solicited adverse events: Solicited systemic adverse events by worst severity grade**

		Stage 1	Stage 2	
**Post dose 1**		**Ad26.ZEBOV**	**Ad26.ZEBOV**	**MenACWY**
**n**		**43**	**298**	**102**
Any solicited systemic event, n (%)	Any	18 (42)	161 (54)	51 (50)
	Grade 1	15 (35)	144 (48)	45 (44)
	Grade 2	3 (7)	14 (5)	6 (6)
	Grade 3	0	3 (1)	0
Arthralgia	Any	4 (9)	57 (19)	23 (23)
	Grade 1	4 (9)	54 (18)	20 (20)
	Grade 2	0	2 (1)	3 (3)
	Grade 3	0	1 (0)	0
Chills	Any	3 (7)	22 (7)	7 (7)
	Grade 1	3 (7)	20 (7)	7 (7)
	Grade 2	0	2 (1)	0
Fatigue	Any	10 (23)	57 (19)	16 (16)
	Grade 1	9 (21)	50 (17)	15 (15)
	Grade 2	1 (2)	7 (2)	1 (1)
Headache	Any	10 (23)	105 (35)	39 (38)
	Grade 1	7 (16)	95 (32)	38 (37)
	Grade 2	3 (7)	9 (3)	1 (1)
	Grade 3	0	1 (0)	0
Myalgia	Any	10 (23)	57 (19)	20 (20)
	Grade 1	9 (21)	54 (18)	19 (19)
	Grade 2	1 (2)	3 (1)	1 (1)
Nausea	Any	1 (2)	6 (2)	0
	Grade 1	1 (2)	5 (2)	0
	Grade 3	0	1 (0)	0
Pyrexia	Any	0	2 (1)	1 (1)
	Grade 1	0	0	1 (1)
	Grade 2	0	1 (0)	0
	Grade 3	0	1 (0)	0

Post dose 2		MVA-BN-Filo	MVA-BN-Filo	Placebo
n		43	246	86
Any solicited systemic event, n (%)	Any	17 (40)	107 (43)	39 (45)
	Grade 1	13 (30)	90 (37)	32 (37)
	Grade 2	3 (7)	17 (7)	6 (7)
	Grade 3	1 (2)	0	1 (1)
Arthralgia	Any	5 (12)	48 (20)	13 (15)
	Grade 1	4 (9)	42 (17)	10 (12)
	Grade 2	1 (2)	6 (2)	3 (3)
Chills	Any	3 (7)	17 (7)	13 (15)
	Grade 1	2 (5)	14 (6)	13 (15)
	Grade 2	1 (2)	3 (1)	0
Fatigue	Any	5 (12)	47 (19)	18 (21)
	Grade 1	2 (5)	43 (17)	15 (17)
	Grade 2	2 (5)	4 (2)	3 (3)
	Grade 3	1 (2)	0	0
Headache	Any	8 (19)	66 (27)	25 (29)
	Grade 1	8 (19)	57 (23)	21 (24)
	Grade 2	0	9 (4)	4 (5)
Myalgia	Any	6 (14)	37 (15)	13 (15)
	Grade 1	6 (14)	34 (14)	10 (12)
	Grade 2	0	3 (1)	3 (3)
Nausea	Any	2 (5)	2 (1)	2 (2)
	Grade 1	1 (2)	2 (1)	2 (2)
	Grade 3	1 (2)	0	0
Pyrexia	Any	0	5 (2)	2 (2)
	Grade 1	0	4 (2)	0
	Grade 2	0	1 (0)	1 (1)
	Grade 3	0	0	1 (1)

Post booster dose		Ad26.ZEBOV
n		29
Any solicited systemic event, n (%)	Any	9 (31)
	Grade 1	7 (24)

	Grade 2	2 (7)
Arthralgia	Any	2 (7)
	Grade 1	2 (7)
Chills	Any	1 (3)
	Grade 2	1 (3)
Fatigue	Any	5 (17)
	Grade 1	4 (14)
	Grade 2	1 (3)
Headache	Any	6 (21)
	Grade 1	6 (21)
Myalgia	Any	2 (7)
	Grade 1	2 (7)
Nausea	Any	0
Pyrexia	Any	0

Vaccines: Ad26.ZEBOV; MVA-BN-Filo. Control: Meningococcal quadrivalent (serogroups A, C, W135 and Y) conjugate vaccine (MenACWY; dose 1), Placebo (dose 2). n=number of participants with data.

2.3. *Table S3*: **Unsolicited adverse events: Most frequent (at least 10% of participants in any vaccination schedule) unsolicited adverse events by system organ class and dictionary-derived term**

	Stage 1	Stage 2	
**Post dose 1**	**Ad26.ZEBOV**	**Ad26.ZEBOV**	**MenACWY**
**n**	**43**	**298**	**102**
Any event, n (%)	17 (40)	198 (66)	65 (64)
Infections and infestations	7 (16)	129 (43)	40 (39)
Malaria	1 (2)	88 (30)	26 (26)
Nervous system disorders	7 (16)	33 (11)	10 (10)
Headache	7 (16)	32 (11)	10 (10)
Musculoskeletal and connective tissue disorders	4 (9)	24 (8)	14 (14)
**Post dose 2**	**MVA-BN-Filo**	**MVA-BN-Filo**	**Placebo**
**n**	**43**	**246**	**86**
Any event, n (%)	17 (40)	145 (59)	48 (56)
Infections and infestations	11 (26)	96 (39)	35 (41)
Malaria	4 (9)	58 (24)	22 (26)
Musculoskeletal and connective tissue disorders	2 (5)	27 (11)	5 (6)
**Post booster dose**	**Ad26.ZEBOV**	-	-
**n**	**29**		
Any event, n (%)	5 (17)		

Vaccines: Ad26.ZEBOV; MVA-BN-Filo. Control: Meningococcal quadrivalent (serogroups A, C, W135 and Y) conjugate vaccine (MenACWY; dose 1), Placebo (dose 2). n=number of participants with data.

This table only includes adverse events that were reported between dose 1 vaccination and 28 days post dose 1, between dose 2 vaccination and 28 days post dose 2, and between Ad26.ZEBOV booster dose vaccination and 28 days post Ad26.ZEBOV booster dose. Adverse events are coded using MedDRA version 21.1.

2.4. *Table S4*: Grade 3 unsolicited adverse events reported after each dose (up to 28 days post dose)

	Stage 1	Stage 2	
**Post dose 1**	**Ad26.ZEBOV**	**Ad26.ZEBOV**	**MenACWY**
**n**	**43**	**298**	**102**
Any event, n (%)	1 (2)	5 (2)	2 (2)
Investigations	0	3 (1)	1 (1)
Haemoglobin decreased	0	3 (1)	1 (1)
Blood and lymphatic system disorders	1 (2)	1 (0)	0
Thrombocytopenia	1 (2)	1 (0)	0
Infections and infestations	0	1 (0)	1 (1)
Brain abscess	0	1 (0)	0
Gastroenteritis	0	0	1 (1)

**Post dose 2**	**MVA-BN-Filo**	**MVA-BN-Filo**	**Placebo**
**n**	**43**	**246**	**86**
Any event, n (%)	1 (2)	5 (2)	1 (1)
Investigations	0	5 (2)	1 (1)
Haemoglobin decreased	0	4 (2)	1 (1)
Aspartate aminotransferase increased	0	1 (0)	0
Blood and lymphatic system disorders	1 (2)	0	0
Anaemia	1 (2)	0	0

**Post booster dose**	**Ad26.ZEBOV**		
**n**	**29**	-	-
Any event, n (%)	0		

Vaccines: Ad26.ZEBOV; MVA-BN-Filo. Control: Meningococcal quadrivalent (serogroups A, C, W135 and Y) conjugate vaccine (MenACWY; dose 1), Placebo (dose 2). n=number of participants with data.

MedDRA SOC term=MedDRA System Organ Class Dictionary-derived Term.
Events are coded using MedDRA version 21.1.

2.5. *Table S5*: Serious adverse events reported during the study

MedDRA SOC term	Stage 1	Stage 2	
	Ad26.ZEBOV, MVA-BN-Filo with an Ad26.ZEBOV booster at 2 years post dose 1	Ad26.ZEBOV, MVA-BN-Filo	MenACWY, Placebo
**Serious adverse events across the entire study, n**	**43**	**298**	**102**
Any event, n (%)	3 (7)	16 (5)	4 (4)
**28-day post dose 1, n**	**43**	**298**	**102**
Any event, n (%)	0	2 (1)	1 (1)
Infections and infestations	0	1 (0)	1 (1)
Brain abscess	0	1 (0)	0
Gastroenteritis	0	0	1 (1)
Subcutaneous abscess	0	1 (0)	0
Injury, poisoning and procedural complications	0	1 (0)	0
Ligament sprain	0	1 (0)	0
Skin laceration	0	1 (0)	0
**Further post-dose 1 FU, n**	**43**	**280**	**96**
Any event, n (%)	0	1 (0)	2 (2)
Infections and infestations	0	1 (0)	2 (2)
Gastroenteritis	0	1 (0)	2 (2)
Nervous system disorders	0	0	1 (1)
Syncope	0	0	1 (1)
**28-day post dose 2, n**	**43**	**246**	**86**
Any event, n (%)	0	0	0
**Further post-dose 2 FU, n**	**43**	**244**	**81**
Any event, n (%)	2 (5)	13 (5)	1 (1)
Infections and infestations	1 (2)	5 (2)	0
Malaria	0	3 (1)	0
Appendicitis	0	2 (1)	0
Gastroenteritis	0	1 (0)	0
Helminthic infection	0	1 (0)	0
Orchitis	1 (2)	0	0
Sepsis	0	1 (0)	0
Injury, poisoning and procedural complications	0	5 (2)	0
Head injury	0	2 (1)	0

Abortion induced incomplete	0	1 (0)	0
Chest injury	0	1 (0)	0
Multiple injuries	0	1 (0)	0
Open globe injury	0	1 (0)	0
Radius fracture	0	1 (0)	0
Blood and lymphatic system disorders	0	2 (1)	0
Anaemia	0	1 (0)	0
Anaemia of pregnancy	0	1 (0)	0
Gastrointestinal disorders	1 (2)	1 (0)	0
Abdominal pain	0	1 (0)	0
Peptic ulcer	1 (2)	0	0
Metabolism and nutrition disorders	0	1 (0)	0
Dehydration*	0	1 (0)	0
Pregnancy, puerperium and perinatal conditions	0	1 (0)	0
Abortion threatened	0	1 (0)	0
Haemorrhage in pregnancy	0	1 (0)	0
Placenta praevia	0	1 (0)	0
Premature labour	0	1 (0)	0
Renal and urinary disorders	0	0	1 (1)
Renal haematoma	0	0	1 (1)
Vascular disorders	0	1 (0)	0
Hypovolaemic shock	0	1 (0)	0
**28-day post booster dose (given at 2 years post dose 1), n**	**29**	-	-
Any event, n (%)	0		
**Further post booster dose FU, n**	**28**	-	-
Any event, n (%)	1 (4)		
Eye disorders	1 (4)		
Retinal detachment	1 (4)		
Infections and infestations	1 (4)		
Chorioretinitis	1 (4)		

Vaccines: Ad26.ZEBOV; MVA-BN-Filo. Control: Meningococcal quadrivalent (serogroups A, C, W135 and Y) conjugate vaccine (MenACWY; dose 1), Placebo (dose 2). FU=follow up. Further post-dose 1 FU: from 29 days post dose 1 to dose 2 or date of last contact if lost to follow-up. Further post-dose 2 FU: from 29 days post dose 2 to booster dose (only in stage 1) or date of last visit or date of last contact if lost to follow-up. Further post-booster dose FU: from 29 days post-booster dose to date of last visit or date of last contact if lost to follow-up; n=number of participants with data.

*Fatal case

2.6. *Table S6:* Ebola glycoprotein-specific binding antibody, stratified by the timing of dose 2 vaccination (dose 2 vaccination received at the protocol planned time, or delayed*)

	Stage 2	
	Ad26.ZEBOV, MVA-BN-Filo	MenACWY, Placebo
**Second dose timing: within protocol-defined window**		
Day 1 (Baseline)		
n	189	66
GMC (95% CI)	69 (56–85)	49 (36–66)
Day 57 (56 days post dose 1)		
n	191	68
GMC (95% CI)	235 (205–269)	50 (37–69)
Responder (n/N*, %)	102/188 (54)	4/66 (6)
(95% CI)	(47–62)	(2–15)
Day 78 (21 days post dose 2)		
n	187	64
GMC (95% CI)	3823 (3334–4383)	48 (<LLOQ–67)
Responder (n/N*, %)	181/184 (98)	2/62 (3)
(95% CI)	(95–100)	(0–11)
Day 360 (359 days post dose 1)		
n	171	62
GMC (95% CI)	258 (222–300)	50 (<LLOQ–71)
Responder (n/N*, %)	84/169 (50)	4/60 (7)
(95% CI)	(42–58)	(2–16)
Day 720 (719 days post dose 1)		
n	159	49
GMC (95% CI)	255 (213–306)	52 (<LLOQ–78)
Responder (n/N*, %)	79/156 (51)	7/48 (15)
(95% CI)	(43–59)	(6–28)
**Second dose timing: Delayed* dose 2**		
Day 1 (Baseline)		
n	53	17
GMC (95% CI)	71 (50–100)	56 (<LLOQ–123)
Day 57 (56 days post dose 1)		
n	54	18
GMC (95% CI)	241 (192–302)	94 (37–238)
Responder (n/N*, %)	34/53 (64)	1/17 (6)
(95% CI)	(50–77)	(0–29)

	Stage 2	
	Ad26.ZEBOV, MVA-BN-Filo	MenACWY, Placebo
Delayed* dose 2 (21 days post dose 2)		
n	46	15
GMC (95% CI)	5761 (3926–8455)	93 (<LLOQ–268)
Responder (n/N*, %)	44/45 (98)	3/14 (21)
(95% CI)	(88–100)	(5–51)
Day 360 (359 days post dose 1)		
n	48	11
GMC (95% CI)	390 (313–486)	45 (<LLOQ–126)
Responder (n/N*, %)	29/47 (62)	2/11 (18)
(95% CI)	(47–76)	(2–52)

Vaccines: Ad26.ZEBOV; MVA-BN-Filo. Control: Meningococcal quadrivalent (serogroups A, C, W135 and Y) conjugate vaccine (MenACWY; dose 1), Placebo (dose 2). n=number of participants with data. CI= confidence interval; GMC=geometric mean concentration; LLOQ=lower limit of quantification; N*=number of participants with data at baseline and at that time point.

GMCs and corresponding CIs are shown as ELISA units/mL.
For the responder rates, Exact Clopper-Pearson CI are shown. A participant was a responder at a considered time point if either: (i) the sample interpretation was negative at baseline and positive post baseline and the post-baseline value was greater than 2·5 x LLOQ; or (ii) if the sample interpretation was positive both at baseline and post baseline and there was a greater than 2·5-fold increase from baseline.

Only Stage 2 participants who received dose 2 are shown.

* Due to a study pause (for precautionary reasons during the evaluation of two SAEs in a different study), in 72 Stage 2 participants dose 2 was delayed (with the time interval between dose 1 and dose 2 ranging from 96 to 147 days)

2.7. *Table S7*: Ebola glycoprotein-specific neutralising antibody concentrations, as measured in a subset of Stage 2 participants

	Stage 2	
	**Ad26.ZEBOV, MVA-BN-Filo**	**MenACWY, placebo**
Day 1 (Baseline)		
n	53	19
GMT (95% CI)	<LLOQ (<LLOQ–<LLOQ)	<LLOQ (<LLOQ–<LLOQ)
Day 57 (56 days post dose 1)		
n	53	19
GMT (95% CI)	<LLOQ (<LLOQ–<LLOQ)	<LLOQ
Responder (n/N*, %)	1/51 (2)	0/19 (0)
(95% CI)	(0–10)	(0–18)
Day 78 (21 days post dose 2)		
n	55	19
GMT (95% CI)	2199 (1634–2960)	<LLOQ
Responder (n/N*, %)	52/53 (98)	0/19 (0)
(95% CI)	(90–100)	(0–18)
Day 360 (359 days post dose 1)		
n	55	19
GMT (95% CI)	<LLOQ (<LLOQ–<LLOQ)	<LLOQ
Responder (n/N*, %)	3/53 (6)	0/19 (0)
(95% CI)	(1–16)	(0–18)
Day 720 (719 days post dose 1)		
n	53	17
GMT (95% CI)	<LLOQ (<LLOQ–126)	<LLOQ
Responder (n/N*, %)	6/51 (12)	0/17 (0)
(95% CI)	(4–24)	(0–20)

Vaccines: Ad26.ZEBOV; MVA-BN-Filo. Control: Meningococcal quadrivalent (serogroups A, C, W135 and Y) conjugate vaccine (MenACWY; dose 1), Placebo (dose 2). n=number of participants with data.
CI=confidence interval; GMC=geometric mean concentration; LLOQ=lower limit of quantification;
N*=number of participants with data at baseline and at that time point.

GMTs and their corresponding CIs are shown on the reported scale (psVNA IC_{50} titer).
For the responder rates, Exact Clopper-Pearson Cis are shown. A participant was a responder at a considered time point either (i) if the sample interpretation was negative at baseline and positive post baseline and the post-

baseline value was greater than 2x LLOQ; or (ii) if the sample interpretation was positive both at baseline and post baseline and there was a greater than 2-fold increase from baseline.

2.8. *Table S8*: Ad26 neutralizing antibodies (Ad26 VNA, IC_{90} titre): Geometric mean and sample interpretation: per protocol analysis set

	Stage 1	Stage 2	
	Ad26.ZEBOV, MVA-BN-Filo	Ad26.ZEBOV, MVA-BN-Filo	Control
Day 1 (Baseline)			
n	43	191	18
GMT (95% CI)	111 (75–163)	124 (101–151)	104 (57–190)
Positive Sample (n (%))	40 (93)	177 (93)	17 (94)
(95% CI)	(81–99)	(88–96)	(73–100)

Vaccines: Ad26.ZEBOV; MVA-BN-Filo. Control: Meningococcal quadrivalent (serogroups A, C, W135 and Y) conjugate vaccine (MenACWY; dose 1), Placebo (dose 2). CI=confidence interval; GMC=geometric mean concentration; n=number of participants with data.

The geometric mean titre and corresponding confidence interval are shown on the reported scale (IC_{90} titre). Exact Clopper-Pearson Confidence Interval is shown for the corresponding sample interpretation rate.

2.9. *Table S9*: **MVA neutralizing antibodies (MVA PRNT, IC_{50} titre): Geometric mean and sample interpretation: per protocol analysis set**

	Stage 1	Stage 2	
	Ad26.ZEBOV, MVA-BN-Filo	Ad26.ZEBOV, MVA-BN-Filo	Control
Day 1 (Baseline)			
n	42	56	18
GMT (95% CI)	<LLOQ (<LLOQ–<LLOQ)	<LLOQ (<LLOQ–<LLOQ)	<LLOQ (<LLOQ–8)
Positive Sample (n (%))	2 (5)	3 (5)	3 (17)
(95% CI)	(1–16)	(1–15)	(4–41)

Vaccines: Ad26.ZEBOV; MVA-BN-Filo. Control: Meningococcal quadrivalent (serogroups A, C, W135 and Y) conjugate vaccine (MenACWY; dose 1), Placebo (dose 2). CI=confidence interval; GMC=geometric mean concentration; n=number of participants with data.

The geometric mean titre and corresponding confidence interval are shown on the reported scale (MVA IC_{50} titre).

Exact Clopper-Pearson confidence interval is shown for the corresponding sample interpretation rate.

2.10. ***Figure S1*: Correlation between Ebola glycoprotein-specific binding antibody concentrations and Ebola glycoprotein-specific neutralizing antibody titres at 21 days post dose 2 in participants who received Ebola vaccine regimen**

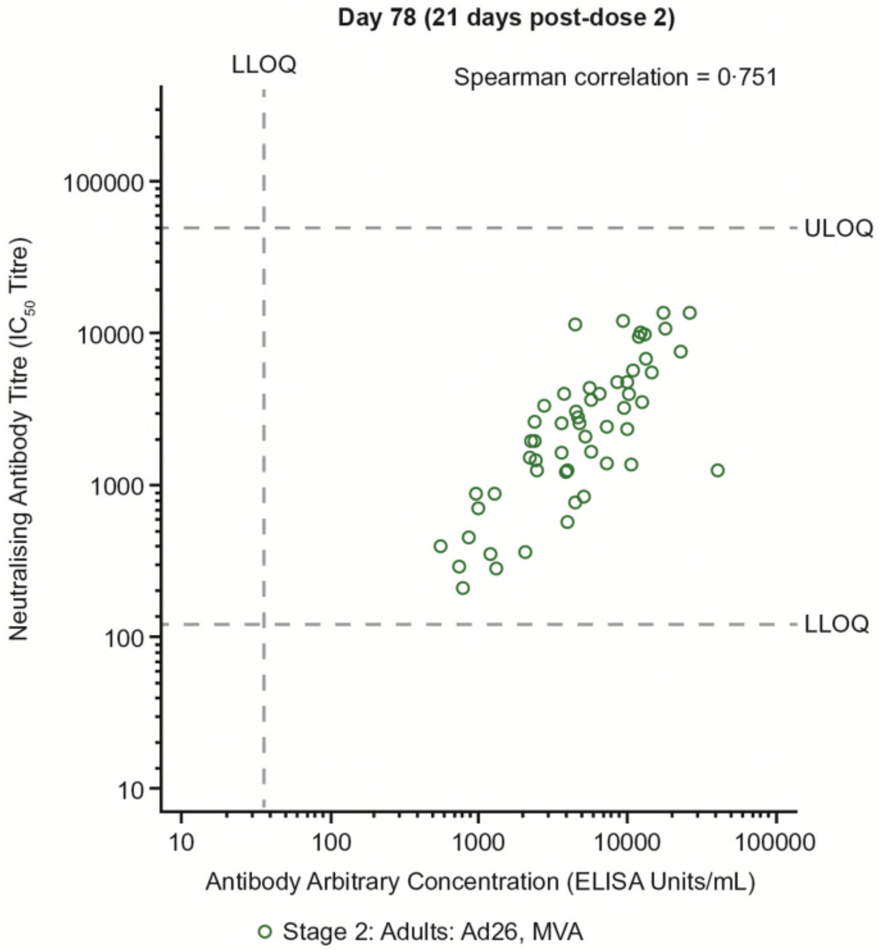

Vaccines: Ad26.ZEBOV (Ad26); MVA-BN-Filo (MVA).
LLOQ=lower limit of quantification; ULOQ=upper limit of quantification. Control participants are not included in this scatter diagram.

2.11. *Figure S2:* **Correlation between baseline Ad26-specific nAb titres and the vaccine-induced EBOV GP-specific binding antibody concentrations at 21 days post-dose 2**

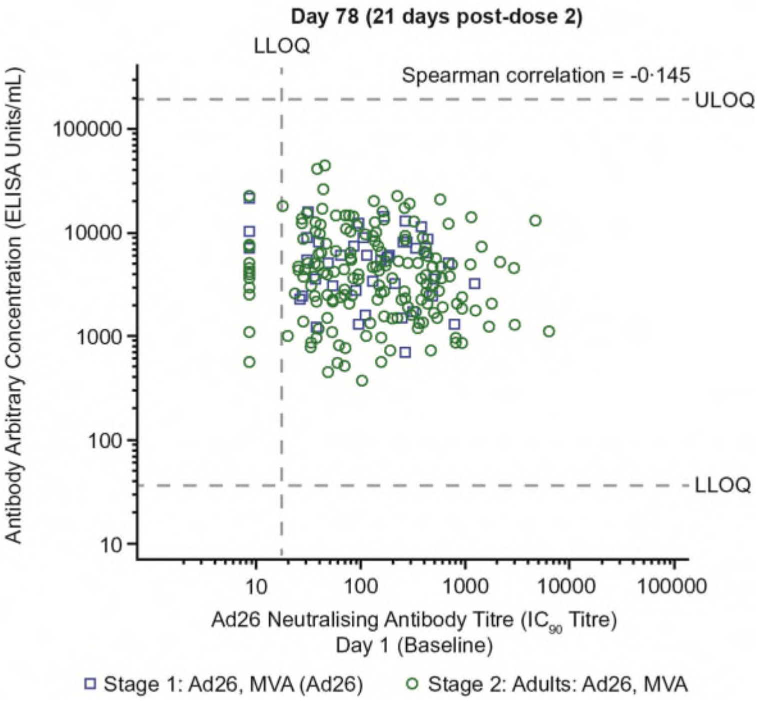

Vaccines: Ad26.ZEBOV (Ad26); MVA-BN-Filo (MVA).
LLOQ=lower limit of quantification; ULOQ=upper limit of quantification. Control participants are not included in this scatter diagram.

2.12. ***Figure S3*: Correlation between pre-existing Ebola glycoprotein-binding antibody at baseline and at 21 days post dose 2 in participants who received Ebola vaccine regimen**

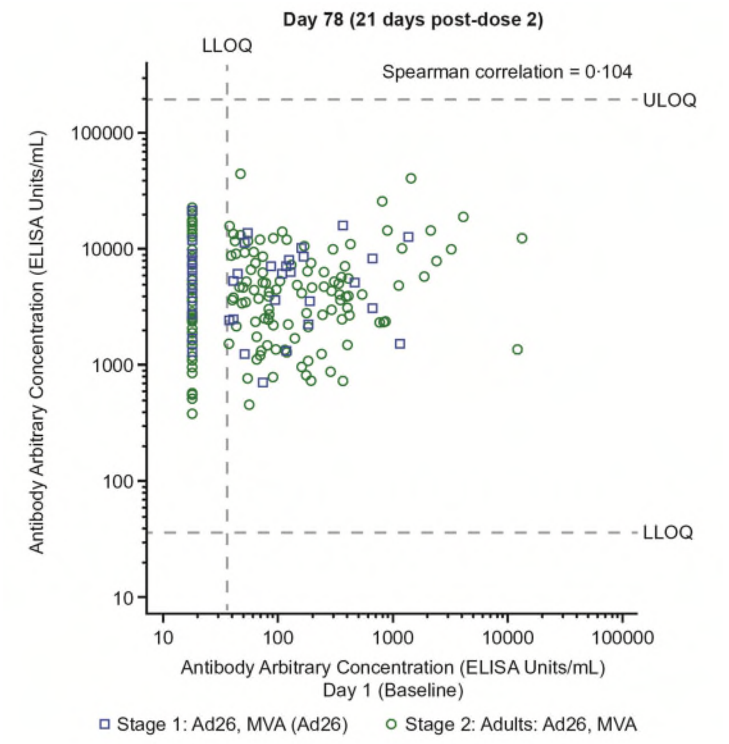

Vaccines: Ad26.ZEBOV (Ad26); MVA-BN-Filo (MVA).
LLOQ=lower limit of quantification; ULOQ=upper limit of quantification. Control participants are not included in this scatter diagram.

3. EBL3001 STUDY GROUP

List of contributors to the study over the four-year study duration period (2015–2019):

Biometrics team

Manager: Moughrphillea Kargbo. Operators: Elizabeth Bockarie, Ndeayah L James, Aminata Kabbah, Aminata Kamara, Kabba H Koroma, Samuel O Langley, Nathaniel William. Technician: Randolph Kessebeh.

Communications and community liaison

Communications Manager: Thomas Mooney. Communications Specialist (Ministry of Health and Sanitation): Lansana Conteh. Community Coordinator: Elizabeth Smout. Community Liaison Assistants: Kadie Allieu, Kadiatu Bangura, Masian S Bangura, Mohamed A Bangura, Hassan Jalloh, Abu Bakarr Jalloh, Isata Kamara, Mohamed Kamara, Adama Konteh, Suad Koroma, Claudia Marrah, Mohamed Sesay, Mohamed Tejan Sesay. Community Liaison Officer: Abdul Tejan Deen.

Community health officers

AbuBakarr Jalloh, Richard M Kaimbay, Desmond Kain, Abdul Kamara, Edward L Kamara, Mohamed Potho Kamara, Osman J Kamara, Ishmael Kamara, Stephen LM Kamara, Mohamed Kanneh, Alhassan H Koroma, Dauda Lahai, Ibrahim S Mansaray, Woria S Marah, Mary J Massaquoi, Abubakarr Nabie, Nyayia S Saidu, Ishmael Samai, Joseph N Tengheh, Andrew S Turay.

Data management

Assistant Data Manager: Alpha Fornah, Francis Sesay, Alphajor Sow, Ernest Swaray, Foday Mansaray. Data clerks: Theodore Ade-Cole, Lamin M Bangura, Mohamed L Conteh, Aminata Kabbah, Augustine M Koroma, Mohamed Koroma, Augustine Sam, Thelma Scott, TutuSessie, Joe-Henry C Sunders, Sorie I-S Turay, James Weekes. Data Manager: Mahmud Sheku. Senior Data Manager: Lorna Gibson (interim), Dickens Kowuor. IT manager: Irrfan Ahamed.

Field workers

Field supervisors: William Allieu, Divine U Kabba, Francess J Kamara, Mohamed S Kebbie, Momoh Pessima, Abdulai Wurie. Field workers: Fatmata Bah, Abdulai I Bangura, Richard AS Bangura, Lovetta Blango, Sia Boima, Memunatu Conteh, Yusufu Conteh, Mohamed L Daramy, Osman Fofanah, Eleanor George, Thelma F Hanson, Mariama I Jalloh, Mathew Kalawa, Abdulai M Kamara, Francis E Kamara, Gibril M Kamara, Hassanatu M Kamara, Posseh BD Kamara, Ramatu T Kamara, Rugiatu Kamara, Derick P Kanneh, Moses Kanneh, Ishmael Komeh, Musu Koroma, Maseray Kuyateh, Fatmata F Mansaray, Marina M Mansaray, Akim B Sillah, Musa A Tarawally, Osman S Turya, James B Yawmah.

Investigators (principals and directors)

Principal Investigator (COMAHS): Bailah Leigh. Principal Investigators (LSHTM): Deborah Watson-Jones, Brian Greenwood. Investigator / COMAHS Scientific Director: Mohamed H Samai. Investigator / COMAHS Clinical Director: Gibrilla Fadlu Deen. Investigator / EPI Director: Dennis Marke. LSHTM senior team: Peter Piot, Peter Smith, John Edmunds, Shelley Lees, Heidi Larson, Helen Weiss, Patrick Wilson.

Investigators and site coordination and medical team (site)

Epidemiologist: Caroline Maxwell. Investigator / Trial Manager: David Ishola. Investigators / Trial Coordinators: Muhammed Afolabi, Frank Baiden. Trial Coordinators: Pauline Akoo, Kwabena Owusu-Kyei, Daniel Tindanbil. Trial Manager: Hilary Bower (interim), James Stuart (interim). Investigators / Senior study doctors: Osman M Bah, Baimba T Rogers, Alimamy Serry-Bangura, Ibrahim Bob Swaray. Investigators / Study doctors: Agnes Bangura, Ifeolu John David, David GM Davies, Joseph Alpha Kallon, Abu Bakarr Kamara, Ibrahim F Kamara, Michael M Kamara, Foday E Morovia, Foday B Suma, Frederick Thompson. Kambia District Medical Officers: Mariama Murray, Foday Sesay. Kambia Government Hospital Medical Superintendents: Osman Kakay, Foday Suma, Isaac Sesay.

Coordination and project management team (London)

Assistant Trial Coordinator: Julie Foster, Robert Phillips. Trial Coordinators: Daniela Manno, Kate Gallager, Sharon Cox (interim), Natasha Howard (interim). Project Assistant: Maria Cesay, Paola Torrani, Sarah Sharma. Project managers: Emily Snowden, Thom Banks, Tomas Harber, Jennifer Brown, Kelly Howard, Natalie Melton. Senior Project Managers: Stuart Malcolm, Suzanne Welsh.

Matematical modelling team (London)

Rosalind Eggo, Mario Jendrossek, Carl Pearson.

Janssen team

Johan Van Hoof, Macaya Douoguih, Kim Offergelt, Cynthia Robinson, Babajide Keshinro, Melissa Van Alst, Navdeep Mahajan, Viki Bockstal, Neil Goldstein, Auguste Gaddah, Dirk Heerwegh, Kerstin Luhn, Maarten Leyssen.

Laboratory team

Laboratory Director: Brett Lowe. Laboratory Manager: Ken Awuondo. Laboratory Scientists: Hannah Hafezi, Emma Hancox, Brian Kohn, Godfrey O Tuda. Laboratory Technicians: Fatmata Koroma, Alima Kamara, Gerald Bangura, Mattu T Kroma, Lamin Fofanah, Alfred Pessima, Maariam Rogers, Osman Sheriff. Phlebotomists / Laboratory Assistants: Tosin W Ajala, Joseph Fangawa, Sahr Foday Jr, Issa Jabbie, Bobson Mansaray, Haja A Mansaray, Kandeh Sesay.

MOTECH (mobile technology) team

Analysts: Millicent K Charles, Pamela C Heroe, Mohamed Lamin Karbo, Ibrahim S Yansaneh. Administrators: Seth Gogo Egoeh, Augustin Trye. Consultant: Monica Amponsah.

Monitoring team

ICON Government and Public Health Solutions

Nurses

Nurses: Nasiru D Alghali, Adama Bah, Isata J Bangura, Alrine Cheryl Cole, Kadijatu Fofanah, Saidu Fofanah, Hawa Umu Jalloh, Kadijatu FN Jalloh, Nini Jalloh, Haja U Kabba, Jennifer N Kabba, Marion Kabba, Rashidatu Kamara, Juliana S Kamara, Finda Kanjie, Aminata P Kanu, Iynnah Kargbo, Gladys Kassa-Koroma, Susanette B Koroma, Adikalie Sankoh, Theresa Sankoh, Olaimatu D Sesay, Hellen Wilhem, Cecilia T Williams. Nurse assistants: Isata Bangura, Yeama Ben-Rogers, Adama Jalloh, Fatmata J Jamboria, Nashiratu Kamara, Isha Kanawah, Adama T Kargbo, Isata Swaray. Senior nurses: Lucinda Amara, Isatu Bundu, Hannah B Jakema, Kadiatu Kamara, Millicent F Sheku.

Paediatrics team

Paediatricians: Qadri Adeleye, Irene Akhigbe, Respicious Bakalemwa, Neema P Chami, Tulla Sylvester. Paediatric nurses: Lisa Altmann, Bomposseh Kamara.

Pharmacy / vaccine cold chain team

Cold Chain Coordinator Specialist: Karel van Roey. Cold Chain Officers: Philip Conteh, Moses Samura, Victor Gandie, Mohamed Marrah, Emmanuel Moinina, Joseph Kalokoh. District Pharmacist: Mohamed I Bangura. Lead Pharmacists: Samuel Bosompem, Trudi Hilton (interim), Morrison O Jusu. Pharmacists: Paul Borboh, Augustine S Brima, Amanda FY Caulker, Ahamed Kallon, Bockarie Koroma, Rachel C Macauley, Tamba MD Saquee, Harold I Williams.

Project management group

Administrators: Abdul Rahman Bangura, Juliana Fornah, Bockarie Idriss. Finance Accountant: Marion Sillah. Finance Director: Waltina Mackay. Finance Managers: Belayneh Aleghen, Tamba Murray. HR Director: Joseph Edem-Hotah. IT manager: Tamba Fatorma. Logistics Managers: Frederick Amara, Mohamed Kamara. Logistics Officers: Santigie Bangura, Elba Bonnie, Musa Sannoh. Operations Manager: Alexandra Donaldson, Samuel Ndingi, Dennis Nyaberi, Martin Pereira (interim), Antony Rothwell. Programme Manager: Vanessa Vy. Senior Administrator: Leonard Nyallay.

Quality Assurance officials

Quality Administrators: Augustin Fombah, Samuel Saidu. QA Manager: Emma Hancox, Nicholas Connor.

Registration team

Clerks: Thomas P Dambo, Philip J Fakaba, Mary ME Fatorma, Rexmonda H Freeman, Cordelia L Johnson, Memunatu Kamara, Darlinda B Kogba, Alfred Lahai, Willieta Vincent, Nabieu Yambasu.

Social science team

Graduate Researchers: Mahmood Bangura, Angus Tengbeh. Research Assistants: Kadiatu Bangura, Rosetta Kabia. Transcriber: Alhaji M Nyakoi. Social Science Leads: Mike Callaghan, Luisa Enria, Shona Lee.

4. STUDY PROTOCOL

Janssen Vaccines & Prevention B.V.* #

Clinical Protocol

A Staged Phase 3 Study, Including a Double-Blinded Controlled Stage to Evaluate the Safety and Immunogenicity of Ad26.ZEBOV and MVA-BN-Filo as Candidate Prophylactic Vaccines for Ebola

Protocol VAC52150EBL3001; Phase 3
AMENDMENT 7
EBOVAC – Salone

London School of Hygiene & Tropical Medicine#
Ministry of Health and Sanitation of Sierra Leone (MoHS)
College of Medicine and Allied Health Sciences (COMAHS)
Ebola Vaccine Deployment, Acceptance & Compliance (EBODAC) Program
Innovative Medicines Initiative (IMI)-1

VAC52150 (Ad26.ZEBOV/MVA-BN-Filo [MVA-mBN226B])

*Janssen Vaccines & Prevention B.V. (formerly known as Crucell Holland B.V.) is a Janssen pharmaceutical company of Johnson & Johnson and is hereafter referred to as the sponsor of the study. The sponsor is identified on the Contact Information page that accompanies the protocol.

EBOVAC1 Consortium Partners. Other Consortium Partners are: Institut National de la Santé et de la Recherche Médicale and Oxford University

.

Status: Approved
Date: 2 October 2018
EDMS number: EDMS-ERI-123324264, 10.0

GCP Compliance: This study will be conducted in compliance with Good Clinical Practice, and applicable regulatory requirements.

Confidentiality Statement
The information in this document contains trade secrets and commercial information that are privileged or confidential and may not be disclosed unless such disclosure is required by applicable law or regulations. In any event, persons to whom the information is disclosed must be informed that the information is privileged or confidential and may not be further disclosed by them. These restrictions on disclosure will apply equally to all future information supplied to you that is indicated as privileged or confidential.

TABLE OF CONTENTS

TABLE OF CONTENTS 2

LIST OF ATTACHMENTS 5

LIST OF IN-TEXT TABLES 5

PROTOCOL AMENDMENTS 6

SYNOPSIS 27

TIME AND EVENTS SCHEDULES 34

TIME AND EVENTS SCHEDULE – STAGE 1 34

TIME AND EVENTS SCHEDULE – STAGE 1 CONTINUED (THIRD VACCINATION) 37

TIME AND EVENTS SCHEDULE – STAGE 2 39

ABBREVIATIONS 42

DEFINITIONS OF TERMS 43

1. INTRODUCTION 44
1.1. Background 44
1.2. Risk Benefit Section 50
1.2.1. Known Benefits 50
1.2.2. Potential Benefits 50
1.2.3. Known Risks 50
1.2.4. Potential Risks 51
1.2.5. Unknown Risks 52
1.2.6. Overall Benefit/Risk Assessment 53
1.3. Overall Rationale for the Study 53

2. OBJECTIVES AND HYPOTHESIS 54
2.1. Objectives 54
2.2. Hypothesis 55

3. STUDY DESIGN AND RATIONALE 55
3.1. Overview of Study Design 55
3.2. Study Design Rationale 58

4. SUBJECT POPULATION 59
4.1. Inclusion Criteria 59
4.2. Exclusion Criteria 61
4.3. Prohibitions and Restrictions 64

5. TREATMENT ALLOCATION AND BLINDING 65

6. DOSAGE AND ADMINISTRATION 68

7. TREATMENT COMPLIANCE 69

8. PRESTUDY AND CONCOMITANT THERAPY 69

9. STUDY EVALUATIONS 70
9.1. Study Procedures 70
9.1.1. Overview 70

9.1.2. Screening Phase ... 71
9.1.3. Vaccination Period ... 73
9.1.4. Post-Boost and Post-Third Vaccination Period ... 74
9.1.5. Long-Term Follow-Up ... 74
9.1.6. Evaluation of Fever ... 75
9.1.7. Early Withdrawal ... 76
9.2. Immunogenicity ... 76
9.2.1. Endpoints ... 76
9.2.2. Evaluations ... 77
9.3. Safety ... 77
9.3.1. Endpoints ... 77
9.3.2. Evaluations ... 78
9.4. Sample Collection and Handling ... 81

10. SUBJECT COMPLETION/WITHDRAWAL ... 82
10.1. Completion ... 82
10.2. Discontinuation of Study Vaccine ... 82
10.3. Contraindications to Vaccination ... 83
10.4. Withdrawal From the Study ... 83

11. STATISTICAL METHODS ... 84
11.1. Analysis Sets ... 85
11.2. Subject Information ... 85
11.3. Sample Size Determination ... 85
11.4. Immunogenicity Analyses ... 86
11.5. Safety Analyses ... 87
11.6. Interim Analysis ... 88
11.7. Independent Data Monitoring Committee (IDMC) ... 88
11.7.1. Safety ... 88
11.8. Clinical Steering Committee and Management Committee ... 89
11.9. Pausing Rules ... 89
11.9.1. Stage 1 ... 89
11.9.2. Stage 2 ... 90
11.9.2.1. Adults and Adolescents (Aged ≥12 Years) ... 90
11.9.2.2. Children (Aged ≥1 to 11 Years, Inclusive) ... 90

12. ADVERSE EVENT REPORTING ... 91
12.1. Definitions ... 91
12.1.1. Adverse Event Definitions and Classifications ... 91
12.1.2. Attribution Definitions ... 93
12.1.3. Severity Criteria ... 94
12.2. Special Reporting Situations ... 94
12.3. Procedures ... 95
12.3.1. All Adverse Events ... 95
12.3.2. Serious Adverse Events ... 96
12.3.3. Immediate Reportable Events ... 97
12.3.4. Pregnancy ... 97
12.4. Contacting Sponsor Regarding Safety ... 98

13. PRODUCT QUALITY COMPLAINT HANDLING ... 98
13.1. Procedures ... 98
13.2. Contacting Sponsor Regarding Product Quality ... 98

14. STUDY VACCINE INFORMATION ... 99
14.1. Description of Study Vaccines ... 99
14.1.1. Ad26.ZEBOV ... 99
14.1.2. MVA-BN-Filo ... 99
14.1.3. MenACWY ... 99

14.1.4. Placebo 99
14.2. Packaging and Labeling 99
14.3. Preparation, Handling and Storage 100
14.4. Vaccine Accountability 100

15. STUDY-SPECIFIC MATERIALS 101

16. ETHICAL ASPECTS 101
16.1. Study-Specific Design Considerations 101
16.2. Regulatory Ethics Compliance 102
16.2.1. Investigator Responsibilities 102
16.2.2. Independent Ethics Committee or Institutional Review Board 103
16.2.3. Informed Consent 104
16.2.4. Privacy of Personal Data 105
16.2.5. Long-Term Retention of Samples for Additional Future Research 106

17. ADMINISTRATIVE REQUIREMENTS 107
17.1. Protocol Amendments 107
17.2. Regulatory Documentation 107
17.2.1. Regulatory Approval/Notification 107
17.2.2. Required Prestudy Documentation 107
17.3. Subject Identification, Enrollment, and Screening Logs 108
17.4. Source Documentation 108
17.5. Case Report Form Completion 109
17.6. Data Quality Assurance/Quality Control 110
17.7. Record Retention 110
17.8. Monitoring 110
17.9. Study Completion/Termination 111
17.9.1. Study Completion 111
17.9.2. Study Termination 111
17.10. On-Site Audits 112
17.11. Use of Information and Publication 112

REFERENCES 114

ATTACHMENTS 115

INVESTIGATOR AGREEMENT 129

LAST PAGE 129

LIST OF ATTACHMENTS

Attachment 1: Schematic Presentation of the IDMC Schedule for the Prime and Boost Vaccination 115
Attachment 2: Test of Understanding (TOU) 116
Attachment 3: Fever Management Algorithm 117
Attachment 4: Toxicity Tables for Use in Trials Enrolling Healthy Adults and Adolescents 118
Attachment 5: Toxicity Tables for Use in Trials Enrolling Children Greater Than 3 Months of Age........ 124

LIST OF IN-TEXT TABLES

Table 1: Number of Subjects Planned per Stage and Population for Safety and Immunogenicity Assessments 32
Table 2: Study Vaccination Schedules 68
Table 3: Summary of Potential Immunologic Assays (Serology)........ 77
Table 4: Number of Subjects Planned per Stage and Population for Safety and Immunogenicity Assessments 85
Table 5: Probability of Observing at Least One (Serious) Adverse Event........ 86
Table 6: Sample Size Justification Safety Evaluation in Stage 1 and 2 86

PROTOCOL AMENDMENTS

Protocol Version	Issue Date
Original Protocol	20-Mar-2015
Amendment 1	01-May-2015
Amendment 2	30-Nov-2015
Amendment 3	28-Jan-2016
Amendment 4	07-Sep-2016
Amendment 5	04-May-2017
Amendment 6	20-June-2018
Amendment 7	02-Oct-2018

Amendments below are listed beginning with the most recent amendment.

Amendment_7 (02-Oct-2018)

The overall reason for the amendment: This amendment is created to update the information on study unblinding and to update information on the roll-over of subjects from the VAC52150EBL3001 study to the VAC52150EBL3005 study.

The changes made to the clinical protocol VAC52150EBL3001 are listed below, including the rationale of each change and a list of all applicable sections.

Rationale: It is clarified that for each age group of Stage 2, subjects and study-site personnel (except for unblinded qualified study-site personnel with primary responsibility for study vaccine preparation and dispensing) will be blinded to the study vaccine allocation until the last subject in that age group has completed at least the 6 month post-boost visit or discontinued earlier and the database has been locked for that part.

SYNOPSIS

3.1 Overview of Study Design

5 TREATMENT ALLOCATION AND BLINDING

11 STATISTICAL METHODS

Rationale: It is clarified that roll-over of subjects in Stage 2 of the VAC52150EBL3001 to the VAC52150EBL3005 study will occur after unblinding of the VAC52150EBL3001 data and that only those subjects who received Ad26.ZEBOV and/or MVA-BN-Filo may roll-over.

SYNOPSIS

3.1 Overview of Study Design

Rationale: The protocol is adjusted to indicate that, in case there would have been females in Stage 1 of the study who became pregnant and met the criteria as outlined in the protocol, they would have been allowed to enroll in the follow-up study VAC52150EBL3005.

SYNOPSIS

3.1 Overview of Study Design

Rationale: Minor corrections have been made.

1.2.3 Known Risks

1.2.4 Potential Risks

3.1 Overview of Study Design

14.4 Vaccine Accountability

Amendment_6 (20-June-2018)

The overall reason for the amendment: This amendment is created to replace information on Study VAC52150EBL4001 with information on the new planned long-term follow-up Study VAC52150EBL3005, to remove references to the Menveo® vaccine, to add information on the requirement to consent or to give positive assent for subjects who turn 18 or 7 years of age, respectively, during active participation in the study, and to add that subjects in Stage 1 and Stage 2 may be contacted approximately 1 month after their last study visit to inquire after their general health status.

The changes made to the clinical protocol VAC52150EBL3001 are listed below, including the rationale of each change and a list of all applicable sections.

Rationale: The planned VAC52150 Vaccine Development Roll-over Study, VAC52150EBL4001, has been replaced by the long-term follow-up Study VAC52150EBL3005. Subjects enrolled in VAC52150EBL3001 who were eligible to be followed-up in the VAC52150EBL4001 study (ie, female subjects who became pregnant with estimated conception within 28 days after vaccination with MVA-BN-Filo [or placebo] or within 3 months after vaccination with Ad26.ZEBOV [or active control vaccine] and children born to vaccinated female subjects who became pregnant with estimated conception within 28 days after vaccination with MVA-BN-Filo [or placebo] or within 3 months after vaccination with Ad26.ZEBOV [or active control vaccine]) will now be eligible to roll-over to the VAC52150EBL3005 study as will all other subjects enrolled in Stage 2 of the current Study VAC52150EBL3001. VAC52150EBL3005 will be a long-term follow-up study evaluating up to 5-year safety and binding antibody persistence of Ad26.ZEBOV at a dose of $5x10^{10}$ viral particles (vp) followed by MVA-BN®-Filo at a dose of $1x10^{8}$ TCID50 56 days later. Since subjects in Stage 1 will be asked to consent to a third open-label vaccination at least 2 years post-prime vaccination, these subjects will not be eligible for long-term follow-up in Study VAC52150EBL3005.

SYNOPSIS

1.2.4 Potential Risks

1.2.6 Overall Benefit/Risk Assessment

3.1 Overview of Study Design

9.1.5 Long-Term Follow-Up

9.x VAC52150 Vaccine Roll-over Study

12.3.4 Pregnancy

Rationale: To allow for WHO-prequalified MenACWY vaccines, other than Menveo®, to be used in the active control group of Stage 2 of the current study VAC52150EBL3001, references to the Tradename Menveo® have been deleted from the protocol.

SYNOPSIS

1.2.1 Known Benefits

1.2.3 Known Risks

1.2.5 Unknown Risks

6 DOSAGE AND ADMINISTRATION

14.1.3 MenACWY

REFERENCES

ATTACHMENTS

Rationale: The prohibitions and restrictions criterion 4.1 was clarified, ie, subjects should not receive inactivated vaccines from 15 days before or after administration of prime and boost vaccination and 15 days before or after administration of the third vaccination in Stage 1 (previously 15 days before or after administration of any study vaccine).

4.3 Prohibitions and Restrictions

Rationale: It has been clarified that subjects who turn 18 or 7 years old while they are still active participants in the study will need to consent or give positive assent, respectively, to continue participation in the study.

16.2.3 Informed Consent

Rationale: Subjects in Stage 1 and Stage 2 may be contacted approximately 1 month after their last study visit to inquire after their general health status.

SYNOPSIS
TIME AND EVENTS SCHEDULE – Stage 1
TIME AND EVENTS SCHEDULE – Stage 1 Continued (Third Vaccination)
TIME AND EVENTS SCHEDULE – Stage 2
3.1 Overview of Study Design
9.1.5 Long-Term Follow-Up
10.1 Completion

Rationale: Minor textual changes, clarifications and corrections have been made.

1 INTRODUCTION
5 TREATMENT ALLOCATION AND BLINDING
9.1.2 Screening Phase
9.1.3 Vaccination Period
9.3.2 Evaluations
16.1 Study-Specific Design Considerations
17.9.1 Study Completion
Attachment 1: Schematic Presentation of the IDMC Schedule for the Prime and Boost Vaccination
Attachment 4: Toxicity Tables for Use in Trials Enrolling Healthy Adults and Adolescents
Attachment 5: Toxicity Tables for Use in Trials Enrolling Children Greater Than 3 Months of Age

Amendment_5 (04-May-2017)

The overall reason for the amendment: This amendment is developed in response to emerging clinical data and changes to the global clinical development plan.

Immunogenicity data from a Phase 1 study of the monovalent vaccine program (VAC52150EBL1002) demonstrated stability of the binding antibody response out to one year following prime-boost regimens with Ad26.ZEBOV/MVA-BN-Filo (or the reverse). A third vaccination using Ad26.ZEBOV at $5x10^{10}$ vp was given at the one-year time point leading to a marked and rapid increase in the binding antibody responses within 7 days (at least 10-fold). The marked and rapid rise was generally independent of the antecedent prime-boost regimen. The profile of the antibody response strongly suggests that robust anamnestic responses can be induced after re-exposure to an EBOV antigen, in this case mimicked by a third vaccination. This amendment proposes administration of a third vaccination using Ad26.ZEBOV at least 2 years post prime for subjects in Stage 1 in order to extend the findings to an African population.

This amendment also proposes to double the number of children in each age group in Stage 2 to expand the safety and immunogenicity data in each age group. The follow-up period of adults in Stage 2 will be extended to 2 years post prime to monitor for safety and persistence of the immune response.

Furthermore, the enrollment of subjects from VAC52150EBL3001 to the VAC52150EBL4001 roll-over study is limited to female subjects who became pregnant with estimated conception within 28 days after vaccination with MVA-BN-Filo (or placebo) or within 3 months after vaccination with Ad26.ZEBOV (or active control vaccine) and children born to vaccinated female subjects who became pregnant with estimated conception within 28 days after vaccination with MVA-BN-Filo or within 3 months after vaccination with Ad26.ZEBOV, unless local regulations have additional requirements for follow up.

The changes made to the clinical protocol VAC52150EBL3001 are listed below, including the rationale of each change and a list of all applicable sections.

Rationale: A third vaccination using Ad26.ZEBOV will be administered to subjects in Stage 1 at least 2 years post-prime in order to confirm and extend the immunogenicity findings from a Phase 1 study.

SYNOPSIS

TIME AND EVENTS SCHEDULE – Stage 1

TIME AND EVENTS SCHEDULE – Stage 1 Continued (Third Vaccination)

1.2.4 Potential Risks

1.2.6 Overall Benefit/Risk Assessment

1.3 Overall Rationale for the Study

2.1 Objectives

3 STUDY DESIGN AND RATIONALE

4.3 Prohibitions and Restrictions

5 TREATMENT ALLOCATION AND BLINDING

6 DOSAGE AND ADMINISTRATION

8 PRESTUDY AND CONCOMITANT THERAPY

9.1.1 Overview

9.1.2 Screening Phase

9.1.3 Vaccination Period

9.1.4 Post-Boost and Post-Third Vaccination Period

9.1.5 Long-Term Follow-Up

9.1.8 VAC52150 Vaccine Roll-over Study

9.3.1 Endpoints

9.3.2 Evaluations

10.1 Completion

10.3 Contraindications to Vaccination

11.3 Sample Size Determination
12.3.1 All Adverse Events
12.3.4 Pregnancy
15 STUDY-SPECIFIC MATERIALS
16.1 Study-Specific Design Considerations
17.4 Source Documentation

Rationale: The number of children in each age group in Stage 2 will be increased to expand the safety and immunogenicity data in each age group. To compensate for an expected higher variability in immunogenicity parameters, sample size will be doubled for each age group. Doubling the sample size will compensate for a 41% higher standard deviation (sqrt(2)=1.41).

SYNOPSIS
1.2.6 Overall Benefit/Risk Assessment
3.1 Overview of Study Design
4 SUBJECT POPULATION
6 DOSAGE AND ADMINISTRATION
11.3 Sample Size Determination

Rationale: The follow-up period of adults in Stage 2 will be expanded to 2 years post prime to monitor for safety and persistence of the immune response.

SYNOPSIS
TIME AND EVENTS SCHEDULE – Stage 2
1.2.6 Overall Benefit/Risk Assessment
3.1 Overview of Study Design
5 TREATMENT ALLOCATION AND BLINDING
9.1.1 Overview
9.1.5 Long-Term Follow-Up
9.3.2 Evaluations
10.1 Completion
12.3.1 All Adverse Events

Rationale: The original development plan (at the time of the ongoing Ebola epidemic in Africa) was an accelerated plan with the anticipation of conducting Phase 3 efficacy studies (with limited safety data collection) shortly after Phase 1 and in parallel with Phase 2. The sponsor designed the VAC52150EBL4001 study for the extended follow-up of serious adverse events to enhance the ability for signal detection of rare events. Since there is no longer an ongoing Ebola epidemic, it is currently not possible to conduct a parallel Phase 3 efficacy study as part of an accelerated development plan. The ongoing Phase 2 and 3 studies will continue long-term follow-up in a placebo-controlled manner until agreement to unblind has been achieved. Placebo-controlled blinded data during long-term follow-up will provide high quality data on long-term safety and will supplement the open-label registry data. In this study, the requirement for entrance to the VAC52150EBL4001 roll-over study is limited to female subjects who became pregnant with estimated conception within 28 days after vaccination with MVA-BN-Filo (or placebo) or within 3 months after vaccination with Ad26.ZEBOV (or active control vaccine) and children born to vaccinated female subjects who became pregnant with estimated conception within 28 days after vaccination with MVA-BN-Filo (or placebo) or within 3 months after vaccination with Ad26.ZEBOV (or active control vaccine), unless local regulations have additional requirements for follow up. Following these children for a total of five years was a commitment made to the relevant health authorities.

SYNOPSIS

3.1 Overview of Study Design

9.1.8 VAC52150 Vaccine Roll-over Study

12.3.4 Pregnancy

Rationale: The secondary objectives and endpoints were limited to immune responses measured by ELISA at 21 days post boost. Immune responses measured by ELISA at all other relevant time points were shifted to the exploratory objectives and endpoints. As the exploratory endpoints can be presented in a separate biomarker report, there will be no delay in the preparation of the final Clinical Study Report.

SYNOPSIS

2.1 Objectives

9.2.1 Endpoints

Rationale: As there is no analysis planned for the immunogenicity sample taken on Day 29 in children from Stage 2, this sample was removed.

TIME AND EVENTS SCHEDULE – Stage 2

9.1.1 Overview

9.1.3 Vaccination Period

16.1 Study-Specific Design Considerations

Rationale: The statistical methods for clinical laboratory tests, vital signs, and physical examination were revised. Since only small mean fluctuations are expected, limited interest will be on the summary statistics over time and the analyses will focus on worst abnormalities and toxicity gradings.

11.5 Safety Analyses

Rationale: As the lab values mentioned in inclusion criterion 4 (Stage 2) are not fully in line with the normal lab ranges, especially for children, this inclusion criteria was modified to keep it more general, without specifying any lab values.

4.1 Inclusion Criteria

Rationale: Exclusion criterion 13 of Stage 2, describing the weight restrictions for children, was modified to reflect the data according to the World Health Organization (WHO) and Centers for Disease Control and Prevention (CDC) growth charts.

4.2 Exclusion Criteria

Rationale: To ensure consistency regarding the completion of the TOU for children whose parent or guardian are not part of the study compared to those who are enrolled to the study, and to clarify the procedures to follow in case of recruitment of multiple children of the same family, the language regarding completion of the TOU has been changed.

TIME AND EVENTS SCHEDULE – Stage 2

9.1.2 Screening Phase

16.1 Study-Specific Design Considerations

Rationale: Minor textual changes and corrections have been made.

SYNOPSIS

TIME AND EVENTS SCHEDULE – Stage 1

TIME AND EVENTS SCHEDULE – Stage 2

ABBREVIATIONS

1.1 Background

1.2.1 Known Benefits

1.2.4 Potential Risks

1.2.6 Overall Benefit/Risk Assessment

1.3 Overall Rationale for the Study

2.2 Hypothesis

3.1 Overview of Study Design

4.1 Inclusion Criteria

6 DOSAGE AND ADMINISTRATION

8 PRESTUDY AND CONCOMITANT THERAPY

9.1.3 Vaccination Period

9.1.5 Long-Term Follow-Up

9.1.8 VAC52150 Vaccine Roll-over Study

9.3.2 Evaluations

11 STATISTICAL METHODS

11.4 Immunogenicity Analyses

11.7 Independent Data Monitoring Committee (IDMC)

11.7.1 Safety

12.3.4 Pregnancy

15 STUDY-SPECIFIC MATERIALS

16.2.3 Informed Consent

Rationale: The protocol has been updated to be in line with the current protocol template (version 1 November 2016).

TITLE PAGE

Amendment_4 (07-Sep-2016)

The overall reason for the amendment: The sponsor halted vaccinations in the clinical program following a case of Miller Fisher syndrome after receipt of MVA-BN-Filo or placebo in study VAC52150EBL2001, as well as following reports of mild transient paresthesia in the same clinical study that required further assessment to rule out a neurologic and autoimmune event. The current study was paused until the updated study-specific informed consent form had been approved to inform the study participants on the additional safety information. As a result of the pause, some subjects were outside the protocol-defined boost vaccination window. Based on the request of the Agence Nationale de Sécurité du Médicament et des produits de santé (ANSM) for study VAC52150EBL2001 in response to the Miller Fisher case, the sponsor has decided to implement the collection of neurologic and autoimmune events ("Immediate Reportable Events") throughout the entire clinical development plan.

The changes made to the clinical protocol VAC52150EBL3001 are listed below, including the rationale of each change and a list of all applicable sections.

Rationale: As requested by the ANSM, wording on the collection of "Immediate Reportable Events" was added after one subject in the study VAC52150EBL2001 experienced a serious and very rare condition called "Miller Fisher syndrome" about a month after boost vaccination with either MVA-BN-Filo or placebo. Although the event was considered to be unrelated to the vaccine, measures were implemented for the entire clinical development program.

SYNOPSIS

Time and Events Schedules

ABBREVIATIONS

1.1 Background

1.2.6 Overall Benefit/Risk Assessment

3.1 Overview of Study Design

9.1.2 Screening Phase

9.1.3 Vaccination Period

9.1.5 Long-Term Follow-Up

9.3 Safety

12.1.1 Adverse Event Definitions and Classifications

12.2 Special Reporting Situations

12.3.1 All Adverse Events

12.3.3 Immediate Reportable Events

Rationale: As a result of the pause, some subjects will be outside the protocol-defined boost vaccination window. Information was added to clarify the procedures that need to be followed for these subjects.

SYNOPSIS

1.2.4 Potential Risks

3.1 Overview of Study Design

11.1 Analysis Sets

Rationale: As a consequence of the pause, delays in unblinding are expected. Further details regarding enrollment into the VAC52150 roll-over study have been added, such as the inclusion of subjects from the control arm before unblinding of the current study.

SYNOPSIS

3.1 Overview of Study Design

9.1.8 VAC52150 Vaccine Roll-over Study

Rationale: As a result of the pause, subjects whose screening period was longer than the protocol-defined 28 days will be allowed to rescreen once.

Time and Events Schedules

9.1.2 Screening Phase

Rationale: Stage 1 of the study is extended for 24 months beyond Day 360, for long-term follow-up of safety and immunogenicity. Subjects in Stage 1 will not be approached to consent for enrollment into the VAC52150 Vaccine Roll-over Study.

SYNOPSIS

Time and Events Schedules

1.2.6 Overall Benefit/Risk Assessment

3.1 Overview of Study Design

9.1.2 Screening Phase

9.1.5 Long-Term Follow-Up

9.3 Safety

10.1 Completion

11 STATISTICAL METHODS

12.3.1 All Adverse Events

Rationale: The visits after the boost vaccination in Stage 2 have been renamed to reflect the timing relative to the boost vaccination, to clarify that the intention of the visit is to follow compared to boost rather than to prime, especially for subjects who are out of window due to the pause. This is consistent with other studies in the VAC52150 clinical program.

Time and Events Schedules

9.1.3 Vaccination Period

9.1.4 Post-Boost Vaccination Period

9.1.5 Long-Term Follow-Up

9.3.2 Evaluations

Rationale: As per the prescribing information of MenACWY (MENVEO®), 2 doses are recommended for children aged 12 to 24 months, with an interval of 3 months between the doses. Children aged less than 2 years at the time of the initial vaccination (randomization) will receive a third vaccination on the 3-month post-boost visit with either MenACWY (control arm) or placebo (Ad26.ZEBOV/MVA-BN-Filo arm).

SYNOPSIS

Time and Events Schedules

6 DOSAGE AND ADMINISTRATION

9.1.3 Vaccination Period

9.1.4 Post-Boost Vaccination Period

9.3.1 Endpoints

9.3.2 Evaluations

Rationale: The 3-month post-boost visit (previously named Day 156) in Stage 2 is only applicable for children aged less than 2 years at the time of randomization. This time point was removed for the other populations based on the availability of new data from other studies showing persistent immune responses up to 1 year post vaccination.

SYNOPSIS

Time and Events Schedules

6 DOSAGE AND ADMINISTRATION

9.1.1 Overview

9.1.4 Post-Boost Vaccination Period

Rationale: Safety assessments have been added to the Day 29 and 6-month post-boost visit for subjects in Stage 2, consistent with other studies in the VAC52150 clinical program.

Time and Events Schedules

9.1.3 Vaccination Period

9.1.5 Long-Term Follow-Up

Rationale: As requested by the Pharmacy Board of Sierra Leone (PBSL), subjects in Stage 2 who received a meningitis vaccine in the past are excluded.

4.2 Exclusion Criteria

Rationale: The use of analgesic/antipyretic medications and non-steroidal anti-inflammatory drugs (NSAIDs) is no longer limited to only 24 hours after vaccination. This measure was previously implemented to avoid missing infection with Ebolavirus while it was still circulating.

4.3 Prohibitions and Restrictions

8 PRESTUDY AND CONCOMITANT THERAPY

Rationale: As requested by the Food and Drug Administration (FDA) in response to Amendment #26 to IND16280 the analysis sets have been re-defined. Furthermore, it was clarified that the primary analysis in Stage 2 will be performed separately for adults and children.

SYNOPSIS

5 TREATMENT ALLOCATION AND BLINDING

11 STATISTICAL METHODS

Rationale: As likely all subjects will have data collected for onsite-assessment or diary data for solicited AEs, there is no need to make this distinction.

11.5 Safety Analyses

Rationale: Wording added to clarify that birth control methods should be applied by sexually active female participants.

Time and Events Schedules

1.2.4 Potential Risks

4.1 Inclusion Criteria

4.3 Prohibitions and Restrictions

Rationale: Safety information following MVA-BN-Filo vaccine administration based on the pooled safety data from studies VAC52150EBL1001 and VAC52150EBL1002 has been included.

1.1 Background

1.2.3 Known Risks

1.2.4 Potential Risks

REFERENCES

Rationale: Name change from Crucell Holland B.V. to Janssen Vaccines & Prevention B.V.

Title Page

SYNOPSIS

1 INTRODUCTION

1.3 Overall Rationale for the Study

14.1.1 Ad26.ZEBOV

14.1.2 MVA-BN-Filo

REFERENCES

Rationale: The protocol has been updated to be in line with the current protocol template (version 6 June 2016).

4 SUBJECT POPULATION

17.4 Source Documentation

Rationale: Minor textual changes have been made, in addition to modifications for clarity and updates to be in line with other current protocols and the current Investigator Brochures.

Throughout the protocol.

Amendment_3 (28-Jan-2016)

The overall reason for the amendment: This amendment is written to delete Stage 3.

The main objective of the originally planned Stage 3 of the study was to assess vaccine effectiveness in preventing cases of Ebola Virus Disease (EVD). As active human-to-human transmission is no longer occurring in Sierra Leone, the window of opportunity to evaluate the effectiveness is closed for this epidemic unless there is a resurgence. As a result, the Stage 3 component has been removed.

Rationale: Deletion of Stage 3 and minor changes

Title Page

SYNOPSIS

Time and Events Schedules

ABBREVIATIONS

1 INTRODUCTION
1.2.2 Potential Benefits
1.2.4 Potential Risks
1.2.6 Overall Benefit/Risk Assessment
1.3 Overall Rationale for the Study
2.1 Objectives
2.x Hypothesis
3 STUDY DESIGN AND RATIONALE
3.1 Overview of Study Design
3.2 Study Design Rationale
4 SUBJECT POPULATION
4.1 Inclusion Criteria
4.2 Exclusion Criteria
4.3 Prohibitions and Restrictions
5 TREATMENT ALLOCATION AND BLINDING
6 DOSAGE AND ADMINISTRATION
8 PRESTUDY AND CONCOMITANT THERAPY
9.1.1 Overview
9.1.2 Screening Phase
9.1.3 Vaccination Period
9.1.4 Post-Boost Vaccination Period
9.1.5 Long-Term Follow-Up
9.1.6 Evaluation of Fever
9.1.x Active and Passive Surveillance of Cases
9.1.8 VAC52150 Vaccine Roll-over Study
9.x Effectiveness
9.x Endpoints
9.x Evaluations
9.x Contact Sub-Study
9.2.2 Evaluations
9.3.1 Endpoints
9.3.2 Evaluations
10.1 Completion
10.4 Withdrawal from the Study
11 STATISTICAL METHODS
11.1 Analysis Sets
11.2 Subject Information
11.3 Sample Size Determination
11.x Effectiveness Analyses
11.x Primary Analysis
11.x Secondary Analysis
11.5 Safety Analyses

11.7 Independent Data Monitoring Committee (IDMC)
11.7.1 Safety
11.x Effectiveness
11.x Stage 3
12.1.1 Adverse Event Definitions and Classifications
12.3.1 All Adverse Events
14.2 Packaging and Labeling
15 STUDY-SPECIFIC MATERIALS
16.2.3 Informed Consent
16.2.5 Long-Term Retention of Samples for Additional Future Research
17.2.1 Regulatory Approval/Notification
17.4 Source Documentation
17.5 Case Report Form Completion
17.8 Monitoring
17.9.1 Study Completion
REFERENCES

Attachment 3 Fever Management Algorithm

Amendment_2 (30-Nov-2015)

The overall reason for the amendment: This amendment is written to adapt Stage 2.

With the declaration of Sierra Leone as Ebola free on 7 November 2015, and in line with the regulatory feedback from FDA and EMA, a control arm has been added to Stage 2 of the study. Stage 2 will now be an individually randomized, double-blinded, active-controlled study. As a consequence, the sample size has been increased to approximately 688 subjects, including some 288 children aged 1 to 17 years. Other key changes include an adjustment in the age limits of the 2 younger age groups (from 1-5 years and 6-11 years to 1-3 years and 4-11 years) as suggested by FDA/EMA feedback. A 3:1 randomization was chosen for the study to expose as many subjects to the vaccine as possible while still having a robust number of control subjects. Subjects in the active control group will receive the WHO-prequalified vaccine MenACWY (MENVEO®, Novartis, which is indicated for active immunization of persons at risk of exposure to *Neisseria meningitidis* groups A, C, W135 and Y) as prime on Day 1 and placebo as boost on Day 57. Children younger than 2 years of age will receive a second dose of MenACWY as boost on Day 57 instead of placebo (as per the prescribing information). Blood samples will be collected for immunogenicity and for safety laboratory assessments, and diaries will be used for collection of solicited local and systemic adverse events, including reactogenicity. No effectiveness data will be captured in Stage 2.

The main objective of the originally planned Stage 3 of the study was to assess vaccine effectiveness in preventing cases of Ebola virus disease (EVD). As active human-to-human transmission is no longer occurring in Sierra Leone, the window of opportunity to evaluate the effectiveness is closed for this epidemic unless there is a resurgence. The Stage 3 component, that was shaded in grey throughout the protocol to facilitate the review of the Sierra Leone Pharmacy Board and Ethics Committee to focus on the Stage 1 and Stage 2 component of the study, continues to be greyed out.

Rationale: Changes of Stage 2

Title Page
SYNOPSIS
Time and Events Schedules
ABBREVIATIONS
DEFINITIONS OF TERMS
1.2 Risk Benefit Section
2.1 Objectives
3 STUDY DESIGN AND RATIONALE
4 SUBJECT POPULATION
5 TREATMENT ALLOCATION AND BLINDING
6 DOSAGE AND ADMINISTRATION
9.1.1 Overview
9.1.2 Screening Phase
9.1.3 Vaccination Period
9.1.4 Post-Boost Vaccination Period
9.1.5 Long-Term Follow-Up
9.2 Effectiveness
9.2.1 Endpoints
9.2.2 Evaluations
9.3.1 Endpoints
9.3.2 Evaluations
10.1 Completion
11.3 Sample Size Determination
11.4 Immunogenicity Analyses

11.5 Safety Analyses
11.7.1 Safety
11.8.2 Effectiveness
11.9 Pausing Rules
12.1.1 Adverse Event Definitions and Classifications
12.3.1 All Adverse Events
14.1.3 MenACWY
14.1.4 Placebo
14.3 Preparation, Handling and Storage
15 STUDY-SPECIFIC MATERIALS
16.1 Study-Specific Design Considerations
17.8 Monitoring
17.9.1 Study Completion
REFERENCES

Attachment 1 Schematic Presentation of the IDMC Schedule for the Prime and Boost Vaccination

---

Rationale: The immunogenicity objective for Stage 1 has been made a secondary instead of a primary objective for alignment with Stage 2.

SYNOPSIS
2.1 Objectives
3.2 Study Design Rationale

Rationale: The protocol is broadened to allow countries other than Sierra Leona to potentially participate in the study. Including additional countries will accelerate availability of sufficient data for submission purposes and will allow for additional countries to engage in Stage 2 of the study, and in Stage 3 in case of a resurgence of the Ebola epidemic in their country.

SYNOPSIS
3.1 Overview of Study Design
4 SUBJECT POPULATION
4.3 Prohibitions and Restrictions
16.2.3 Informed Consent

Rationale: A schematic presentation of the IDMC schedule has been added to clarify the process.

SYNOPSIS
3.1 Overview of Study Design
11.7 Independent Data Monitoring Committee (IDMC)
Attachment 1 Schematic Presentation of the IDMC Schedule for the Prime and Boost Vaccination

---

Rationale: Harmonization of the body temperature designated as fever (≥38.0°C) has been done for Stage 2 and 3. The temperature in Stage 1 will not be adapted as all subjects will be vaccinated by the time this amendment will go into effect.

SYNOPSIS
4.2 Exclusion Criteria
10.3 Contraindications to Vaccination

**Rationale:** It has been clarified that immunological assays to be performed will depend on assay and/or sample availability.
SYNOPSIS<br>9.2 Immunogenicity<br>16.2.5 Long-Term Retention of Samples for Additional Future Research
**Rationale:** The blood volumes to be collected have been adapted to reflect a change in the collection tubes for immunogenicity samples in children, and RT-PRC testing for export of samples.
Time and Events Schedules<br>9.1.1 Overview<br>16.1 Study-Specific Design Considerations
**Rationale:** For Stage 2, a Day 156 (for adults only) and Day 240 time point was chosen, in addition to the already specified time points, to draw blood for an immunogenicity assay.
Time and Events Schedules<br>9.1.4 Post-Boost Vaccination Period<br>9.1.5 Long-Term Follow-Up
**Rationale:** The introduction was aligned with the Edition 3 of the Ad26.ZEBOV Investigator Brochure
1 INTRODUCTION<br>REFERENCES
**Rationale:** The VAC52150 Vaccine Development Registry has been replaced by a roll-over study
1.2.4 Potential Risks<br>9.1.8 VAC52150 Vaccine Roll-over Study<br>12.3.4 Pregnancy
**Rationale:** Malaria and other endemic parasitic diseases can lead to a decrease in white blood cell count compared to that found in subjects in non-malaria regions. Therefore, based on the laboratory normal ranges expected in West Africa, the cut-off for inclusion in Stage 2 has been adapted from 4,000 to 3,500 white blood cells per mm^3.
4.1 Inclusion Criteria
**Rationale:** Text on hemoglobin, hematocrit and red blood cell count in inclusion criterion #4 has been removed as this is already covered sufficiently by exclusion criterion #4. This is applicable to Stage 2.
4.1 Inclusion Criteria
**Rationale:** Screening failures in Stage 1 and 2 will be allowed to volunteer for participation at a later stage.
4.1 Inclusion Criteria<br>4.2 Exclusion Criteria
**Rationale:** To align with the Phase 2 protocols, subjects in Stage 2 can retake the Test of Understanding twice instead of once.
4.1 Inclusion Criteria<br>9.1.2 Screening Phase<br>16.1 Study-Specific Design Considerations

**Rationale:** Based on the laboratory normal ranges expected in West Africa, the minimum number of platelets in inclusion criterion #4 has been adapted from 150,000 to 100,000. This is applicable to Stage 2. The adult toxicity grading scale for platelets has been adapted to reflect this change.
4.1 Inclusion Criteria<br>REFERENCES<br>Attachment 4 Toxicity Tables for Use in Trials Enrolling Healthy Adults
**Rationale:** Text has been added to further clarify the community engagement process.
5 TREATMENT ALLOCATION AND BLINDING
**Rationale:** To keep all options open it is specified that interim analyses may be planned to support regulatory purposes and additional text on blinding for interim analyses in Stage 2 is added.
SYNOPSIS<br>5 TREATMENT ALLOCATION AND BLINDING<br>11 STATISTICAL METHODS<br>11.6 Interim Analysis
**Rationale:** Evaluations in case of fever have been clarified and a fever management algorithm has been added. In case a subject has fever, but does not meet the EVD definition, an unscheduled blood sample may be taken to perform a PCR test to exclude EVD. To prevent new malaria infections in the study subjects as much as possible, subjects in Stage 1 and 2 will receive insecticide-treated bed nets to reduce the incidence of malaria-induced fever.
9.1.1 Overview<br>9.1.6 Evaluation of Fever<br>Attachment 3 Fever Management Algorithm
**Rationale:** Clarification on criteria for contraindication of boost vaccination
10.3 Contraindications to Vaccination
**Rationale:** Toxicity grading tables will be used instead of laboratory normal ranges
11.5 Safety Analyses<br>Attachment 4: Toxicity Tables for Use in Trials Enrolling Healthy Adults<br>Attachment 5: Toxicity Tables for Use in Trials Enrolling Children Greater Than 3 Months of Age
**Rationale:** Small adjustments for clarification
Throughout the protocol

Amendment_1 (01-May-2015)

The overall reason for the amendment: This amendment is written in response to the Assisted Review of the Janssen Ebola Zaire Vaccine Clinical Trials Application meeting of 8-10 April 2015. It only concerns changes to the Stage 1 and Stage 2 components of the protocol. Any text related to the Stage 3 component of the protocol is shaded in grey and will be reviewed at a later stage.

Applicable Section(s)	Description of Change(s)
**Rationale:** The following changes were made on request of the Health Authorities:	
Title Page<br>SYNOPSIS<br>3 STUDY DESIGN AND RATIONALE<br>17.2.1 Regulatory Approval/Notification	Explanation is provided that the amendment only concerns changes to the Stage 1 and Stage 2 components of the protocol, and that any text related to the Stage 3 component will be reviewed at a later stage. The title of the study has also been updated to reflect the staged approach.
Title Page<br>1 INTRODUCTION<br>1.3 Overall Rationale for the Study	The role of the Ministry of Health and Sanitation (MoHS), College of Medicine and Allied Health Sciences (COMAHS), and GOAL has been acknowledged.
SYNOPSIS<br>Time and Events Schedules<br>1.2.6 Overall Benefit/Risk Assessment<br>3.1 Overview of Study Design<br>4 SUBJECT POPULATION<br>4.1 Inclusion Criteria<br>5 TREATMENT ALLOCATION AND BLINDING<br>9.1.4 Post-Boost Vaccination Period<br>9.1.5 Long-Term Follow-Up<br>9.3.2 Evaluations<br>16.2.3 Informed Consent	Active surveillance will be performed in a sentinel subsample of 5,000 subjects from the delayed-vaccination clusters who have provided individual consent for this. Subjects in both sentinel subsamples will be followed-up for 6 months.
SYNOPSIS<br>Time and Events Schedules<br>3.1 Overview of Study Design<br>9.1.1 Overview<br>9.1.4 Post-Boost Vaccination Period<br>9.1.5 Long-Term Follow-Up	Subjects from Stage 3 will be followed-up for 1 year for immunogenicity.
SYNOPSIS<br>1.2.6 Overall Benefit/Risk Assessment<br>3.1 Overview of Study Design<br>11.3 Sample Size Determination	Subjects in Stage 2 will be enrolled in 4 age groups.
SYNOPSIS<br>2.1 Objectives	Primary study objectives are defined for Stage 1 and Stage 2.

Applicable Section(s)	Description of Change(s)
SYNOPSIS<br>3.1 Overview of Study Design<br>11.7 Independent Data Monitoring Committee (IDMC)	The review of the Independent Data Monitoring Committee (IDMC) has been aligned with having 4 age groups in Stage 2. In addition, the IDMC review will include data from Phase 1 and Phase 2 studies, and will also take into consideration cardiac or hematological abnormalities.
SYNOPSIS<br>5 TREATMENT ALLOCATION AND BLINDING<br>9.2.1 Endpoints<br>9.2.2 Evaluations<br>11 STATISTICAL METHODS<br>REFERENCES	Clarifications have been added to the statistical sections/analyses. It has been described that the adjudication panel will be blinded.
SYNOPSIS<br>9.2.1 Endpoints<br>11.4.1 Primary Analysis	Clarification is added that the primary analysis will be done on the primary analysis set, which is a subset of the intent-to-treat (ITT) analysis set.
SYNOPSIS<br>9.2.2 Evaluations	The Sierra Leone case definitions for Ebola (adapted from World Health Organization [WHO]) are used.
Time and Events Schedules<br>1.2.4 Potential Risks<br>4.1 Inclusion Criteria<br>4.3 Prohibitions and Restrictions<br>9.1.2 Screening Phase	Contraception has been added to the inclusion criteria.
Time and Events Schedules<br>4.1 Inclusion Criteria<br>16.2.3 Informed Consent	The assent procedure in children/adolescents must be witnessed and documented. There will be no separate assent form.
Time and Events Schedules<br>4.1 Inclusion Criteria<br>9.1.2 Screening Phase<br>16.1 Study-Specific Design Considerations<br>16.2.3 Informed Consent<br>Attachment 2: Test of Understanding (TOU)	The test of understanding (TOU) will be administered after reading, but before signing the informed consent form (ICF).
Time and Events Schedules<br>4.3 Prohibitions and Restrictions<br>8 PRESTUDY AND CONCOMITANT THERAPY	Prestudy and concomitant therapies will be collected in the sentinel cohort from the immediate-vaccination clusters in Stage 3.
Time and Events Schedules<br>9.1.3 Vaccination Period	The observation period after each vaccine administration during which subjects will stay in the clinic may be extended if deemed necessary by the study staff.

Applicable Section(s)	Description of Change(s)
1.2.4 Potential Risks<br>9.1.8 VAC52150 Vaccine Roll-over Study<br>12.3.4 Pregnancy	Pregnancy outcomes of all women who become pregnant during the study until up to 28 days after the boost vaccination or 3 months after the prime vaccination (whichever is longer) will be collected in the VAC52150 Vaccine Development Registry.
4.1 Inclusion Criteria<br>9.3.2 Evaluations	Laboratory parameters have been added to the inclusion criteria.
4.2 Exclusion Criteria<br>4.3 Prohibitions and Restrictions<br>8 PRESTUDY AND CONCOMITANT THERAPY	Subjects should not receive live-attenuated vaccines from 30 days before the prime vaccination until 30 days after the boost vaccination, and inactivated vaccines from 15 days before or after administration of any study vaccine.
4.2 Exclusion Criteria<br>8 PRESTUDY AND CONCOMITANT THERAPY	Anemia has been added to the exclusion criteria. Subjects who develop anemia during the study will be treated with supplements.
4.2 Exclusion Criteria<br>10.3 Contraindications to Vaccination	Illness has been defined.
4.3 Prohibitions and Restrictions<br>5 TREATMENT ALLOCATION AND BLINDING	Text to identify subjects for correct application of the prime-boost regimen has been simplified.
9.1.1 Overview	Minor correction (deletion of blood volume for subjects <18 years in Stage 1).
9.1.6 Evaluation of Fever<br>15 STUDY-SPECIFIC MATERIALS	The algorithm for evaluation of fever was adapted.
9.3.2 Evaluations	Text concerning handling of deaths and autopsies was added.
12.1.3 Severity Criteria<br>Attachment 4: Toxicity Tables for Use in Trials Enrolling Healthy Adults<br>Attachment 5: Toxicity Tables for Use in Trials Enrolling Children Greater Than 3 Months of Age	Toxicity severity grading tables for adverse event, laboratory data and vital signs have been added.
12.3.1 All Adverse Events	Ebola prevention counseling will be provided on the wallet card.
16.2.4 Privacy of Personal Data	Language was added around the transfer of the data to other entities and to other countries.
**Rationale:** The following additional changes were made:	
Title Page	The protocol name was added.
Title Page<br>1 INTRODUCTION	The partners contributing to the study are now listed on the title page.

Applicable Section(s)	Description of Change(s)
SYNOPSIS<br>Time and Events Schedules<br>3.1 Overview of Study Design<br>9.1.3 Vaccination Period<br>9.1.4 Post-Boost Vaccination Period<br>9.1.5 Long-Term Follow-Up<br>9.2.2 Evaluations	Subjects from Stage 1 will participate in the immunogenicity assessments. An additional sample will be collected on Day 240 from subjects in Stage 1 and Stage 2. A pre-prime sample will be collected from subjects in Stage 3 who participate in the immunogenicity sub-study.
ABBREVIATIONS<br>12.1.1 Adverse Event Definitions and Classifications<br>12.3.1 All Adverse Events	The definition of "SUSAR" was aligned with the new protocol template text and the relevant sections of the protocol were updated accordingly.
1.1 Background<br>REFERENCES	The introduction section of the protocol was updated with interim results from the ongoing Phase 1 study that is being conducted in the United Kingdom (VAC52150EBL1001).
3.2 Study Design Rationale	Study design rationale for Stage 1 and Stage 2 was added.
5 TREATMENT ALLOCATION AND BLINDING	The approach for community engagement was clarified.
12.3.2 Serious Adverse Events	Serious adverse event reporting will be done according to local regulations.
16.2.5 Long-Term Retention of Samples for Additional Future Research	A polymerase chain reaction (PCR) test may be done on all immunology samples to ensure Ebola negativity.
17.11 Use of Information and Publication	Text on use of information and publication was updated to reflect the agreements between the sponsor and the Consortium.
Attachment 2 Test of Understanding (TOU)	The TOU language has been changed.
Throughout the protocol	Modifications were made throughout the protocol to correct minor inconsistencies and/or for clarity.

SYNOPSIS

A Staged Phase 3 Study, Including a Double-Blinded Controlled Stage to Evaluate the Safety and Immunogenicity of Ad26.ZEBOV and MVA-BN-Filo as Candidate Prophylactic Vaccines for Ebola

Janssen Vaccines & Prevention B.V. (formerly known as Crucell Holland B.V., hereafter referred to as the sponsor), in collaboration with Bavarian Nordic GmbH, Denmark, and in conjunction with the London School of Hygiene & Tropical Medicine and Sierra Leonean Ministry of Health and Sanitation (MoHS) and College of Medicine and Allied Health Sciences (COMAHS), and the Ebola vaccine Deployment, Acceptance & Compliance (EBODAC) program, is investigating the potential of a prophylactic heterologous prime-boost Ebola vaccine regimen comprising the following 2 candidate Ebola vaccines:

- **Ad26.ZEBOV** (JNJ-61210474-AAA) is a monovalent vaccine expressing the full length Ebola virus (EBOV, formerly known as *Zaire ebolavirus*) Mayinga glycoprotein (GP). The vaccine is produced in the human cell line PER.C6®.
- **MVA-mBN226B** (JNJ-63839880-AAA) (further referred to as MVA-BN-Filo®) is a multivalent vaccine expressing the EBOV GP, the Sudan virus (SUDV) GP, the Marburg virus (MARV) Musoke GP, and the Tai Forest virus (TAFV, formerly known as *Côte d'Ivoire ebolavirus*) nucleoprotein (NP). The EBOV GP expressed by MVA-BN-Filo has 100% homology to the one expressed by Ad26.ZEBOV.

OBJECTIVES AND HYPOTHESIS

OBJECTIVES

The primary objective of Stage 1 is:

- To evaluate the safety of a heterologous prime-boost regimen utilizing Ad26.ZEBOV and MVA-BN-Filo given at a 56-day interval.

The secondary objective of Stage 1 is:

- To assess humoral immune responses to a heterologous prime-boost regimen utilizing Ad26.ZEBOV and MVA-BN-Filo as measured by an enzyme-linked immunosorbent assay (ELISA) at 21 days post-boost vaccination.
- To assess the safety and tolerability of a third vaccination using Ad26.ZEBOV administered at least 2 years post prime.

The exploratory objectives of Stage 1 are:

- To assess humoral immune responses to a heterologous prime-boost regimen utilizing Ad26.ZEBOV and MVA-BN-Filo as measured by ELISA at other relevant time points.
- To assess neutralizing antibody responses directed against EBOV GP induced by the heterologous prime-boost regimen as measured by a virus neutralization assay (VNA) depending on sample and assay availability.
- To assess antibody responses directed against the Ad26 and/or MVA vector as measured by ELISA, VNA, and/or plaque reduction neutralization test (PRNT) depending on sample and assay availability.
- To assess humoral immune responses to a heterologous prime-boost regimen utilizing Ad26.ZEBOV and MVA-BN-Filo as measured by ELISA following a third vaccination using Ad26.ZEBOV at 2 years post prime.

The primary objective of Stage 2 is:

- To evaluate the safety of a heterologous prime-boost regimen utilizing Ad26.ZEBOV and MVA-BN-Filo given at a 56-day interval compared to an active control vaccine.

The secondary objective of Stage 2 is:

- To assess humoral immune responses to a heterologous prime-boost regimen utilizing Ad26.ZEBOV and MVA-BN-Filo as measured by an ELISA at 21 days post boost.

The exploratory objectives of Stage 2 are:

- To assess humoral immune responses to a heterologous prime-boost regimen utilizing Ad26.ZEBOV and MVA-BN-Filo as measured by ELISA at other relevant time points.
- To assess neutralizing antibody responses directed against EBOV GP induced by the heterologous prime-boost regimen as measured by VNA depending on sample and assay availability.
- To assess antibody responses directed against the Ad26 and/or MVA vector as measured by ELISA, VNA, and/or PRNT depending on sample and assay availability.

Note: For the definition of a stage, see section Overview of Study Design below.

HYPOTHESIS

As this study is designed to provide descriptive information regarding safety and immunogenicity without formal treatment comparisons, no formal statistical hypothesis testing is planned.

OVERVIEW OF STUDY DESIGN

This is a staged Phase 3 study with an open-label uncontrolled stage (Stage 1) and a double-blinded controlled stage (Stage 2) to evaluate the immunogenicity and safety of a heterologous prime-boost regimen where Ad26.ZEBOV will be used to prime a filovirus-specific immune response and MVA-BN-Filo will be used to boost the immune response 56 days later. In Stage 1, a third vaccination using Ad26.ZEBOV will be administered at least 2 years post prime to subjects who consent to this.

The study will be conducted as follows:

- Stage 1: The study will commence with vaccination of a group of approximately 40 adult subjects aged 18 years or older. The objective of this initial stage of the study is to evaluate the safety and immunogenicity of the prime-boost regimen in the adult Sierra Leonean population.
- Stage 2: Approximately 976 subjects aged 1 year or older will be individually randomized in a 3:1 ratio to receive the Ad26.ZEBOV/MVA-BN-Filo prime-boost regimen or an active control vaccine and placebo. The aim is to enroll approximately 400 adults (aged 18 years or older) and approximately 576 children aged ≥1 year (with about 192 children in each of the 3 age groups [ie, 12 to 17 years, 4 to 11 years, and 1 to 3 years, inclusive]). Enrollment will be staggered, starting with the eldest age group. The decision to proceed to the next age group will be based on an evaluation by an independent data monitoring committee (IDMC) (for details see Section 11.7). Randomization will be stratified by age group.

Within each age group of Stage 2, study-site personnel (except for those with primary responsibility for study vaccine preparation and dispensing), sponsor personnel (except for specifically designated sponsor personnel who are independent of the study) and subjects will be blinded to the study vaccine until all subjects in that age group have completed at least the 6 month post-boost visit or discontinued earlier and the database has been locked for that age group. Should an interim analysis be performed prior to the 6 months post-boost visit of the last subject in a given age group, only those involved in the analysis and decision making will be unblinded.

An IDMC will be commissioned for this study. The IDMC will review all available 7-day post-prime safety data (including clinical laboratory results) before the first subject of the current cohort receives the boost:

In Stage 1 to determine whether:

  - the boost vaccination in Stage 1 can be initiated, and
  - the prime vaccination in Stage 2 (subjects aged 12 to 17 years inclusive) can be initiated.

- In Stage 2 (subjects aged 12 to 17 years inclusive) to determine whether:
  - the boost vaccination of subjects aged 12 to 17 years inclusive can be initiated, and
  - the prime vaccination of subjects aged 4 to 11 years inclusive can be initiated.
- In Stage 2 (subjects aged 4 to 11 years inclusive) to determine whether:
  - the boost vaccination of subjects aged 4 to 11 years inclusive can be initiated, and
  - the prime vaccination of subjects aged 1 to 3 years inclusive can be initiated.
- In Stage 2 (subjects aged 1 to 3 years inclusive) to determine whether:
  - the boost vaccination of subjects aged 1 to 3 years inclusive can be initiated.

The IDMC will also review all cumulative safety data including the 7-day post-boost safety data (including clinical laboratory results) to determine if the boost vaccination in the next stage and/or next age group can be given. An IDMC meeting will therefore take place with all available data (also including the 7-day post-prime safety data within that age group) before boost vaccination will be administered.

A schematic presentation of the IDMC schedule is provided in Attachment 1.

The IDMC will also assess on an ongoing basis the Stage 1 and Stage 2 data as well as data emerging from Phase 1 and 2 studies and the status of the epidemic.

While Stage 2 of this study was enrolling, the sponsor halted all vaccinations in the clinical program due to the occurrence of a serious and very rare condition, Miller Fisher syndrome, reported in study VAC52150EBL2001, as well as following reports of mild transient paresthesia in the same study, until a revised informed consent form (ICF) was prepared and approval to restart the current study was granted by all relevant authorities. This interruption in dosing affected 98 consented subjects in the current study, who had received the prime vaccination. When approval was granted to restart the study under Amendment 3, a late boost vaccination was offered to those subjects who had only received the prime vaccination, unless participants had withdrawn from the study or were not eligible to receive the boost. Vaccinated subjects will follow the same post-boost vaccination schedule as those subjects unaffected by the pause. Subjects who refuse or are not eligible to receive the late boost will be encouraged to remain in the study to be followed for safety and immunogenicity.

As part of Amendment 4 for this study, Stage 1 was extended for 2 years beyond Day 360 post prime for follow-up of safety and immunogenicity. As part of Amendment 5, subjects in Stage 1 who consent to this will receive a third vaccination (Ad26.ZEBOV) at least 2 years post-prime vaccination. The follow-up period for adult subjects in Stage 2 will be extended for up to 2 years post prime, for those subjects who consent to this.

Female subjects in Stage 1 of the study who became pregnant with estimated conception within 28 days after vaccination with MVA-BN-Filo or within 3 months after vaccination with Ad26.ZEBOV, children born to vaccinated female subjects in Stage 1 who became pregnant with estimated conception within 28 days after vaccination with MVA-BN-Filo or within 3 months after vaccination with Ad26.ZEBOV (unless local regulations have additional requirements for follow up) and subjects enrolled in Stage 2 of the study (including female subjects who became pregnant and children born to vaccinated female subjects

who became pregnant as specified above for Stage 1) may be invited for enrollment into the VAC52150EBL3005 study for long-term follow-up of safety and immunogenicity for a total of up to 60 months after the prime vaccination. Roll-over of subjects in Stage 2 of the study will occur after unblinding of VAC52150EBL3001 and will only include subjects who received Ad26.ZEBOV and/or MVA-BN-Filo.

Immunogenicity Assessments

Immunogenicity assessments for humoral immune responses will be performed in all subjects (adults, adolescents, and children aged 1 year or older) from Stage 1 and Stage 2. Blood samples will be collected as specified in the Time and Events Schedules. Subjects from Stage 1 and Stage 2 will be followed-up for immunogenicity until 36 months post prime or 1-year post-third vaccination (if applicable) in Stage 1, until 1 year post prime for children and adolescents in Stage 2 and until 1 year post prime or 2 years post prime (if applicable) for adults in Stage 2.

SUBJECT POPULATION

The study population will consist of:

- Stage 1: approximately 40 healthy subjects aged 18 years or older.
- Stage 2: approximately 976 healthy subjects aged 1 year or older. The aim is to enroll approximately 400 adults and approximately 576 children aged ≥1 year (192 adolescents aged 12 to 17 years inclusive, 192 children aged 4 to 11 years inclusive, and 192 young children aged 1 to 3 years inclusive). Enrollment will be staggered, starting with the eldest age group. The decision to proceed to the next age group will be based on an evaluation by the IDMC.

HIV-positive subjects can be enrolled as long as their general condition is good, ie, they are on antiretroviral treatment or have no signs or symptoms of immune incompetence, diagnosed on the basis of physical examination, medical history, and the investigator's clinical judgment.

DOSAGE AND ADMINISTRATION

All subjects in Stage 1 and all subjects randomized to the Ad26.ZEBOV/MVA-BN-Filo arms in Stage 2 will receive the following as a 0.5 mL intramuscular (IM) injection into either deltoid in the upper arm (or, if needed, in the thigh):

- Ad26.ZEBOV: $5x10^{10}$ viral particles (vp) on Day 1
- MVA-BN-Filo: $1x10^{8}$ infectious units (Inf U) on Day 57

Subjects in Stage 1 who consent to this will receive a third vaccination using Ad26.ZEBOV at $5x10^{10}$ vp at least 2 years post prime vaccination.

Subjects in the control arm of Stage 2 will receive the World Health Organization (WHO)-prequalified Meningococcal Group A, C, W135 and Y conjugate vaccine (MenACWY) as prime on Day 1 and placebo as boost on Day 57. The MenACWY vaccine will be administered as a 0.5 mL IM injection into either deltoid in the upper arm. Children aged less than 2 years at randomization will receive a third vaccination at 3 months post boost with either placebo (Ad26.ZEBOV/MVA-BN-Filo arm) or MenACWY (control arm).

Study vaccines in Stage 2 will be prepared by an unblinded pharmacist or qualified staff member with primary responsibility for study vaccine preparation and dispensing and who is not involved in any other study-related procedures. Subjects will be administered the study vaccines in a masked syringe in a way that maintains double-blinding.

Upon completion of the study, sites may offer MenACWY to the Ad26.ZEBOV/MVA-BN-Filo arm and the Ad26.ZEBOV/MVA-BN-Filo vaccine (if licensed and/or WHO-prequalified) to the control arm upon consultation with the health authorities.

IMMUNOGENICITY EVALUATIONS

The immunogenicity endpoint of the study includes the evaluation of antibody responses.

Venous blood samples will be collected from all subjects from Stage 1 and Stage 2 to assess humoral immune responses to the vaccines.

Depending on assay and/or sample availability any of the following assays might be performed:

- EBOV GP ELISA: to determine the binding antibody levels elicited by vaccination.
- VNA: neutralizing antibody reactivity against the EBOV GP defined as the serum titer that is able to inhibit viral infection by a certain percentage (IC_{50}; IC_{80} or IC_{90}).
- ELISA, VNA, and/or PRNT: to explore the binding and/or neutralizing antibody response against the adenovirus and/or MVA vector.

SAFETY EVALUATIONS

All safety evaluations (including physical examinations, vital signs, body temperature, and laboratory evaluations in Stage 1 and Stage 2) will be performed as specified in the Time and Events Schedules.

The safety and tolerability endpoints are:

- Solicited local and systemic adverse events (reactogenicity), collected until 7 days after the prime and boost vaccination (Stage 1 and Stage 2) and the third vaccination (Stage 1) as recorded by the subjects (or, when appropriate, by a project field worker, a caregiver, surrogate, or the subject's legally acceptable representative) in a diary during daily home contacts with a project field worker.
- Solicited local and systemic adverse events (reactogenicity), collected until 60 minutes after the prime and boost vaccination and until 30 minutes after the third vaccination in all subjects from Stage 1, and until 30 minutes after the prime and boost vaccination in all subjects from Stage 2.
- Unsolicited adverse events, collected by the investigator or the clinical designee from signing of the ICF onwards until 56 days after the boost vaccination in Stage 1 and then again from the day of the third vaccination until 28 days after the third vaccination, and from signing of the ICF onwards until 28 days after the prime vaccination in Stage 2, and then again until 28 days after the boost vaccination. (Note: events that started before the third vaccination but are still present at the time of third vaccination should also be recorded).
- Serious adverse events, including deaths, and immediate reportable events (IREs) collected in all subjects from Stage 1 and 2 from signing of the ICF onwards until the subject’s last study visit.

STATISTICAL METHODS

The primary analysis of immunogenicity and safety in Stage 1 will be performed when all subjects have completed the study or have discontinued earlier. In Stage 2, primary analyses will be performed for adults (≥18 years) and children (≥1 to 17 years) when these respective groups complete the study or subjects have discontinued earlier. One primary analysis for all children is planned, presenting the data by age group (12 to 17 years, 4 to 11 years, 1 to 3 years, inclusive). Interim analyses may be performed during the study per age group (ie, ≥18 years, 12 to 17 years, 4 to 11 years, 1 to 3 years, inclusive) for the purpose of informing future program-related decisions and for regulatory purposes in a timely manner.

Sample Size

Table 1 shows the numbers of subjects planned for safety and immunogenicity by age group for Stage 1 and Stage 2.

Table 1: **Number of Subjects Planned per Stage and Population for Safety and Immunogenicity Assessments**

	Safety and Immunogenicity Data		
	Stage 1	***Stage 2***	
**Population Age (years inclusive)**	Ebola vaccine[a]	Ebola vaccine[b]	Active control[c]
Adults (≥18 years)	**40**	**300**	**100**
12 to 17 years	-	**144**	**48**
4 to 11 years	-	**144**	**48**
1 to 3 years	-	**144**	**48**
**Total**	**40**	**732**	**244**

[a] Prime-boost regimen of Ad26.ZEBOV followed by MVA-BN-Filo (boost vaccination) and Ad26.ZEBOV (third vaccination).
[b] Prime-boost regimen of Ad26.ZEBOV followed by MVA-BN-Filo.
[c] The active control will consist of the WHO-prequalified MenACWY vaccine.

For safety, the sample sizes selected indicate that the probability of observing at least 1 (serious) adverse event occurring at a rate of 1/10 or more in each group (Stage 1; adults, Stage 2; adults, 12-17 years, 4-11 years, and 1-3 years) is ≥99%. The probability of observing at least 1 (serious) adverse event occurring at a rate of 1/100 is 95% with 300 subjects. The probability of observing at least 1 (serious) adverse event occurring at a rate of 1/1,000 is 26% with 300 subjects and 52% with 732 subjects. If no events are observed during Stage 2 for a specific adverse event, then the Bayesian posterior probability that the event rate is below 1/1,000 equals 41% for 144 subjects (ie, the number of children in each age group), 56% for 300 subjects (ie, the number of adults), and 77% with 732 subjects when using Jeffreys' prior.

As part of Amendment 5, the number of children in each age group was doubled to compensate for an expected higher variability in immunogenicity parameters.

Immunogenicity Analyses

Descriptive statistics (actual values, changes from baseline, if applicable) will be calculated for continuous immunologic parameters at all time points analyzed. Geometric mean concentrations and/or titers and changes together with a corresponding 95% confidence interval will be calculated. Graphical representations of changes in immunologic parameters will be prepared, as applicable. Frequency tabulations will be calculated for discrete (qualitative) immunologic parameters at all time points and response analyses will be performed. Different definitions of immunologic response will be further detailed in the statistical analysis plan (SAP).

In addition, a longitudinal data analysis of the immunogenicity response over time will be explored. Least square means with their 95% confidence intervals will be calculated from this model.

Safety Analyses

No formal statistical testing of safety data is planned. Safety analysis will be performed on the safety analysis set. Data from Stage 1 will be summarized separately. Safety data from Stage 2 will be shown by treatment group (as treated), and by age group.

Safety data will be analyzed descriptively (including 95% confidence intervals, if applicable) for subjects receiving control or active vaccine.

TIME AND EVENTS SCHEDULES

TIME AND EVENTS SCHEDULE – STAGE 1

Study Procedures	Screening (≤28 days) [a]	Study Period								Long-Term Follow-Up[o]			
		D1	D2-7	D8	D57 ±1w	D58-63	D64	D78 ±1w	D156 ±1w	D180 ±2w	D240 ±1m	D360 ±1m	Every 6 Months until 36 Months Post Prime ±1m[n]
		Prime		+7d pp	Boost		+7d pb	+21d pb	+155d pp	+6m pp	+8m pp	+1y pp	
Informed consent [b]	X												
Inclusion/exclusion criteria	X	X [c]											
Demographics	X												
Medical history/pre-study medication [d]	X												
Full physical examination [e]	X												
Test of Understanding (TOU) [f]	X												
**Study Visits**													
Study vaccine administration		▲			▼ [c]								
Visit to the clinic [g]	X	X		X	X		X	X	X	X	X	X	X
Home visits [h]			X	X [i]		X	X [i]	X [i]	X [i]	X [i]	X [i]	X [i]	X [i]
**Diary**													
Distribution of subject diary		X			X								
Review and collection of subject diary				X			X						
**Assessments**													
Pregnancy test [j]	X	X [k]			X [k]								
Concomitant medications	X	X		X	X		X	X	X				
Brief physical examination [e]		X		X	X		X	X	X				
Vital signs incl. body temperature	X	X [l]		X	X [l]		X	X	X				
Chemistry, hematology	X	X [k]		X	X [k]		X	X					
Review and collection of solicited symptoms		X	X	X	X	X	X						
Blood sampling for humoral assays		X [k]			X [k]			X	X			X	X
Adverse event recording	Continuous until 56 days after the boost vaccination												

Serious adverse event and immediate reportable events (IREs) recording	Continuous

Study Procedures	Screening (≤28 days) a)	Study Period								Long-Term Follow-Up			
		D1	D2-7	D8	D57 ±1w	D58-63	D64	D78 ±1w	D156 ±1w	D180 ±2w	D240 ±1m	D360 ±1m	Every 6 Months until 36 Months Post Prime ±1m n)
		Prime		+7d pp	Boost		+7d pb	+21d pb	+155d pp	+6m pp	+8m pp	+1y pp	
**Approximate Blood Volumes**													
Safety: 4.5 mL per blood draw	4.5	4.5		4.5	4.5		4.5	4.5					
Immunogenicity: 10 mL per blood draw m)		10.0			10.0			10.0	11.2			11.2	11.2
**Total cumulative (safety+immunogenicity)**	**4.5**	**19.0**		**23.5**	**38.0**		**42.5**	**57.0**	**68.2**			**79.4**	**124.2**

▲ *Ad26.ZEBOV 5x10^{10} vp* ▼ *MVA-BN-Filo 1x10^{8} Inf U*

D: day; m: month; w: week(s)

d pp: days post prime; d pb: days post boost; m pp: months post prime; y pp: year post prime

NOTE: If a subject did not receive study vaccine on the planned day of vaccination, the timings of the next visits post-vaccination will be determined relative to the actual day of vaccination. Day 64 will take place 7 days after the boost vaccination.

NOTE: In case of early withdrawal due to an adverse event, the investigator or designee will collect all information relevant to the adverse event and safety of the subject, and will follow the subject until resolution of the adverse event or until reaching a clinically stable endpoint. Subjects who wish to withdraw consent will be offered an optional visit for safety follow-up (before the formal withdrawal of consent). The subject has the right to refuse.

a) Screening will take place within 28 days of the prime vaccination. Screening may be split into multiple days or visits. Retesting of values (eg, safety laboratory) that lead to exclusion is allowed once using an unscheduled visit during the screening period.
b) Informed consent must be provided before the first study-related activity. Adult subjects (aged 18 years or older at screening) must sign an informed consent form (ICF) indicating that he or she understands the purpose of and procedures required for the study and is willing to participate in the study. In case the subject cannot read or write, the procedures must be explained and informed consent must be witnessed by a literate third party not involved in the conduct of the study.
c) The investigator or designee should ensure that all study enrollment criteria have been met at the end of the screening period. If a subject’s clinical status changes (including available laboratory results or the receipt of additional medical records) after screening but before Day 1 such that the subject no longer meets all eligibility criteria, then the subject will be excluded from participation in the study. For contraindications to receive the boost vaccination, refer to Section 10.3.
d) Prestudy therapies up to 30 days prior to the start of screening must be recorded.
e) The full physical examination includes body length/height and weight for all subjects.
f) The TOU will be administered after reading but before signing the ICF.
g) Visit to a clinic where subject is seen by a study nurse or physician. Subjects in Stage 1 will be asked to stay in the clinic for 60 minutes after each vaccination. The observation time may be extended if deemed necessary by the study staff.

h) Daily home contacts by project field worker to record local and systemic side effects.
i) A home visit will be arranged in case it is not possible for the subject to visit the clinic. The diary should be completed by the end of the Day 8 and Day 64 visit, respectively.
j) For female subjects of childbearing potential, it should be confirmed that adequate birth control measures consistent with what is available and local standards regarding the use of birth control for subjects participating in clinical studies were used from at least 14 days before the prime vaccination with a negative urine β-human chorionic gonadotropin (β-hCG) pregnancy test at screening and immediately prior to each study vaccine administration.
k) Prior to study vaccine administration.
l) Prior to study vaccine administration and at the end of the observation period.
m) Polymerase chain reaction (PCR) testing for Ebola will be performed before immunogenicity samples are exported. This can be done on leftovers from the hematology blood sample. On visits where no hematology sample is taken, an additional 1.2 mL blood will be drawn for PCR testing (additional volumes counted as part of the immunogenicity sample).
n) Subjects who consent to receive the third vaccination will follow this schedule until the Day 540 visit; thereafter, they will follow the next TIME AND EVENTS SCHEDULE – Stage 1 Continued (Third Vaccination).
o) Subjects may be contacted approximately 1 month after their last visit to inquire after their general health status.

TIME AND EVENTS SCHEDULE – STAGE 1 CONTINUED (THIRD VACCINATION)

	**Study Period**					**Long-Term Follow-Up**[m)]	
	**2 Years Post Prime +3m**	**D2-D7 Post 3rd Vacc.**	**D5 Post 3rd Vacc.**	**D8 Post 3rd Vacc.**	**D22 Post 3rd Vacc. ±1w**	**6 Months Post 3rd Vacc. ±1m**	**D360 Post 3rd Vacc. ±1m**
**Study Procedures**	**Third Vaccination**		**+4d p3v**	**+7d p3v**	**+21d p3v**	**+6m p3v**	**+1y p3v**
Informed consent [a)]	X						
**Study Visits**							
Study vaccine administration	▲[b)]						
Visit to the clinic [c)]	X		X	X	X	X	X
Home visits [d)]		X	X[e)]	X[e)]	X[e)]	X[e)]	X[e)]
**Diary**							
Distribution of subject diary	X						
Review and collection of subject diary				X			
**Assessments**							
Pregnancy test [f)]	X[g)]						
Concomitant medications	X		X	X	X[j)]	X[j)]	X[j)]
Brief physical examination [i)]	X			X	X	X[j)]	X[j)]
Vital signs incl. body temperature	X[k)]						
Chemistry, hematology	X[g)]			X			
Review and collection of solicited symptoms	X		X	X			
Blood sampling for humoral assays	X		X	X	X	X	X
Adverse event recording	Continuous until 28 days after the third vaccination						
Serious adverse event and immediate reportable events (IREs) recording	Continuous						
**Approximate Blood Volumes**							
Safety: 4.5 mL per blood draw	4.5			4.5			
Immunogenicity: 10 mL per blood draw [l)]	10		11.2	10	11.2	11.2	11.2
**Total cumulative (safety+immunogenicity)**	**105.1**		**116.3**	**130.8**	**142.0**	**153.2**	**164.4**

▲ *Ad26.ZEBOV 5x10^{10} vp*

D: day; m: month; Vacc: vaccination; w: week(s)

d p3v: days post-third vaccination; m p3v: months post-third vaccination

NOTE: If a subject did not receive study vaccine on the planned day of vaccination, the timings of the next visits post-vaccination will be determined relative to the actual day of vaccination.

NOTE: In case of early withdrawal due to an adverse event, the investigator or designee will collect all information relevant to the adverse event and safety of the subject, and will follow the subject until resolution of the adverse event or until reaching a clinically stable endpoint. Subjects who wish to withdraw consent will be offered an optional visit for safety follow-up (before the formal withdrawal of consent). The subject has the right to refuse.

a) Informed consent must be provided before the first study-related activity of the third vaccination schedule. Adult subjects (aged 18 years or older at screening) must sign an ICF indicating that he or she understands the purpose of and procedures required for the study and is willing to participate in the study. In case the subject cannot read or write, the procedures must be explained and informed consent must be witnessed by a literate third party not involved in the conduct of the study.
b) For contraindications to receive the third vaccination, refer to Section 10.3.
c) Visit to a clinic where subject is seen by a study nurse or physician. Subjects will be asked to stay in the clinic for 30 minutes after the third vaccination. The observation time may be extended if deemed necessary by the study staff.
d) Daily home contacts by project field worker to record local and systemic side effects.
e) A home visit will be arranged in case it is not possible for the subject to visit the clinic. The diary should be completed by the end of the Day 8 post-third visit.
f) For female subjects of childbearing potential, it should be confirmed that adequate birth control measures consistent with what is available and local standards regarding the use of birth control for subjects participating in clinical studies were used from at least 14 days before the third vaccination with a negative urine β-hCG pregnancy test immediately prior to vaccine administration.
g) Prior to study vaccine administration.
h) Concomitant therapies should be recorded up to 28 days after the third vaccination. Thereafter, they should only be recorded if given in conjunction with serious adverse events and IREs.
i) Does not include body length/height and weight.
j) During the long-term follow-up, a brief physical examination should only be performed when related to serious adverse events and IREs.
k) Prior to study vaccine administration and at the end of the observation period.
l) PCR testing for Ebola will be performed before immunogenicity samples are exported. This can be done on leftovers from the hematology blood sample. On visits where no hematology sample is taken, an additional 1.2 mL blood will be drawn for PCR testing (additional volumes counted as part of the immunogenicity sample).
m) Subjects may be contacted approximately 1 month after their last visit to inquire after their general health status.

TIME AND EVENTS SCHEDULE – STAGE 2

Study Procedures	Screening (≤28 days)[a]	Study Period									Long-Term Follow-Up			
		**D1**	**D2-7**	**D8**	**D29 ±1 week**	**D57 ±1 week**	**D2-7 Post Boost**	**D8 Post Boost**	**D22 Post Boost ±1 week**	**3 Months Post Boost [b] ±1 week**	**6 Months Post Boost[t] ±1 month**	**D360 ±1 month**	**D540 ±1 month [c]**	**2 Years Post Prime ±1 month[c),t)]**
		**Prime**		**+7d pp**	**+28d pp**	**Boost**		**+7d pb**	**+21d pb**	**+3m pb**	**+6m pb**	**+1y pp**	**+18m pp**	**+2y pp**
Informed consent [d]	X													
Inclusion/exclusion criteria	X	X [e]												
Demographics	X													
Medical history/pre-study medication [f]	X													
Full physical examination [g]	X													
Test of Understanding (TOU) [h]	X													
**Study Visits**														
Randomization		X												
Study vaccine administration		▲				▼ [e]				♦ [e) i)]				
Visit to the clinic or vaccination post [j]	X	X		X	X	X		X	X	X	X	X	X	X
Home visits [k]			X	X[l]			X	X[l]						
**Diary**														
Distribution of subject diary		X				X								
Review and collection of subject diary				X				X						
**Assessments**														
Pregnancy test [m]	X	X[n]				X[n]								
Concomitant medications [o]	X	X		X	X	X		X	X	X	X	X	X	X
Brief physical examination [g]		X		X	X	X		X	X	X	X[p]	X[p]	X[p]	X[p]
Vital signs incl. body temperature	X	X[q]		X	X	X[q]		X	X	X	X			
Chemistry, hematology	X	X[n]		X		X [n]		X						
Review and collection of solicited symptoms		X	X	X		X	X	X						
Blood sampling for humoral assays		X[n]			X[r]	X[n]			X		X	X		X
Adverse event recording	Continuous until 28 days post prime					Continuous until 28 days post boost								
Serious adverse event and immediate reportable events (IREs) recording	Continuous													

Study Procedures	Screening (≤28 days)[a)]	Study Period									Long-Term Follow-Up			
		D1	D2-7	D8	D29 ±1 week	D57 ±1 week	D2-7 Post Boost	D8 Post Boost	D22 Post Boost ±1 week	3 Months Post Boost [b)] ±1 week	6 Months Post Boost ±1 month	D360 ±1 month	D540 ±1 month [c)]	2 Years Post Prime ±1 month [c),t)]
		Prime		+7d pp	+28d pp	Boost		+7d pb	+21d pb	+3m pb	+6m pb	+1y pp	+18m pp	+2y pp
**Approximate Blood Volumes**														
Safety: 1-<18 years (2.4 mL per blood draw)	2.4	2.4		2.4		2.4		2.4						
≥18 years (4.5 mL per blood draw)	4.5	4.5		4.5		4.5		4.5						
Immunogenicity: [s)]														
1-<6 year (2.5 mL per blood draw)		2.5				2.5			3.7		3.7	3.7		
≥6-<13 year (5.0 mL per blood draw)		5.0				5.0			6.2		6.2	6.2		
≥13-<18 year (10.0 mL per blood draw)		10.0				10.0			11.2		11.2	11.2		
≥18 year (10.0 mL per blood draw)		10.0			11.2	10.0			11.2		11.2	11.2		11.2
**Total cumulative (safety + immunogenicity)**														
**1-<6 year**	**2.4**	**7.3**		**9.7**		**14.6**		**17.0**	**20.7**		**24.4**	**28.1**		
**≥6-<13 year**	**2.4**	**9.8**		**12.2**		**19.6**		**22.0**	**28.2**		**34.4**	**40.6**		
**≥13 year <18 year**	**2.4**	**14.8**		**17.2**		**29.6**		**32.0**	**43.2**		**54.4**	**65.6**		
**≥18 year**	**4.5**	**19.0**		**23.5**	**34.7**	**49.2**		**53.7**	**64.9**		**76.1**	**87.3**		**98.5**

▲ *Ad26.ZEBOV 5x10^{10} vp or active control vaccine (MenACWY)* ▼ *MVA-BN-Filo 1x10^{8} Inf U, MenACWY or placebo* ♦ *MenACWY or placebo (only for young children aged less than 2 years)*
d pp: days post prime; d pb: days post boost; m pb: months post boost; y pp: year post prime

NOTE: If a subject did not receive study vaccine on the planned day of vaccination, the timings of the next visits post-vaccination will be determined relative to the actual day of vaccination.

NOTE: In case of early withdrawal due to an adverse event, the investigator or designee will collect all information relevant to the adverse event and safety of the subject, and will follow the subject until resolution of the adverse event or until reaching a clinically stable endpoint. Subjects who wish to withdraw consent will be offered an optional visit for safety follow-up (before the formal withdrawal of consent). The subject has the right to refuse.

a) Screening will take place within 28 days of the prime vaccination. Screening may be split into multiple days or visits. Retesting of values (eg, safety laboratory) that lead to exclusion is allowed once using an unscheduled visit during the screening period. Similarly, subjects that are rescreened due to the pause must have new safety laboratory assessments within 28 days of the new randomization date.
b) The 3-month post-boost visit is only applicable for children aged less than 2 years at randomization.
c) Only applicable for adults who consent to extend the follow-up period for up to 2 years post prime.
d) Informed consent must be provided before the first study-related activity. Adult subjects (aged 18 years or older at screening) must sign an ICF indicating that he or she understands the purpose of and procedures required for the study and is willing to participate in the study. In case the subject cannot read or write, the procedures must be

explained and informed consent must be witnessed by a literate third party not involved in the conduct of the study. If the subject's age is below the legal cut-off age for consent (according to local regulations), the parent/legal guardian will be asked to give consent, and the subject will be informed about the study. Subjects aged 7 years and older will be asked to give positive assent. The assent procedure must be witnessed by an adult literate parent/legal guardian/third party not involved in the conduct of the study, and documented.

e) The investigator or designee should ensure that all study enrollment criteria have been met at the end of the screening period. If a subject's clinical status changes (including available laboratory results or the receipt of additional medical records) after screening but before Day 1 such that the subject no longer meets all eligibility criteria, then the subject will be excluded from participation in the study. For contraindications to receive the boost vaccination, refer to Section 10.3.

f) Prestudy therapies administered up to 30 days prior to the start of screening must be recorded at screening.

g) The full physical examination includes body length/height and weight for all subjects. The brief physical examination includes body length/height and weight for children aged 12 to 24 months prior to the prime and boost vaccination. For details, refer to Section 9.3.2.

h) For children younger than 18 years, the TOU will be administered to the parent or guardian from whom consent is sought. The TOU will be administered after reading but before signing the ICF. If both the parent or guardian and the child will be enrolled in the study, the parent/guardian will need to complete the TOU twice (ie, one for the parent/guardian and one for the child). In case the parent/guardian consents for multiple children of the same family, the TOU needs to be completed only once, ie, for the first child of the family enrolled in the study. In case the TOU information would change while recruiting multiple children of the same family, the TOU should be completed again for the first child of the family that was enrolled after the TOU change had occurred.

i) A third vaccination (MenACWY or placebo) will be administered to children aged less than 2 years. Subject diaries will not be distributed. Children that experience any symptoms will be invited to come for an unscheduled visit as needed.

j) Visit to a clinic or satellite vaccination post where subject is seen by a study nurse or physician. Subjects will be asked to stay in the clinic/vaccination post for 30 minutes after the prima and boost vaccination and symptoms of unsolicited and solicited local and systemic adverse events will be documented at the end of the observation period. The observation time may be extended if deemed necessary by the study staff.

k) Daily home contacts by project field worker to record local and systemic side effects.

l) A home visit could be arranged in case it is not possible for the subject to visit the clinic to ensure the collection of local and systemic reactions. The diary should be completed and collected at the Day 8 and Day 8 post-boost visit, respectively.

m) For sexually active female subjects of childbearing potential, it should be confirmed that adequate birth control measures consistent with what is available and the local standards regarding the use of birth control for subjects participating in clinical studies were used from at least 14 days before the prime vaccination with a negative urine β-hCG pregnancy test at screening and immediately prior to each study vaccine administration.

n) Prior to study vaccine administration.

o) Concomitant therapies should be recorded from screening onwards until 28 days post prime and then again until 28 days post-boost vaccination. At the other time points, it should only be recorded if given in conjunction with serious adverse events and IREs.

p) During the long-term follow-up, a brief physical examination should only be performed when related to serious adverse events and IREs.

q) Prior to study vaccine administration and at the end of the observation period.

r) Only applicable for adults.

s) PCR testing for Ebola will be performed before immunogenicity samples are exported. This can be done on leftovers from the hematology blood sample. On visits where no hematology sample is taken, an additional 1.2 mL blood will be drawn for PCR testing (additional volumes counted as part of the immunogenicity sample).

t) Subjects may be contacted approximately 1 month after their last visit to inquire after their general health status.

ABBREVIATIONS

Ad	adenoviral vector (serotype indicated by a number, eg, Ad26)
Ad26.ENVA.01	Ad26 vector expressing the human immunodeficiency virus type 1, Clade A envelope protein
Ad26.ZEBOV	Ad26 vector expressing the glycoprotein of the Ebola virus Mayinga variant
ALT	alanine aminotransferase
AST	aspartate aminotransferase
β-hCG	β-human chorionic gonadotropin
BMI	body mass index
BN	Bavarian Nordic
CDC	Centers for Disease Control and Prevention
COMAHS	College of Medicine and Allied Health Sciences
CRF	case report form
EBODAC	Ebola vaccine Deployment, Acceptance & Compliance
EBOV	Ebola virus
eDC	electronic data capture
ELISA	enzyme-linked immunosorbent assay
Env	Envelope
EU	European Union
EVD	Ebola Virus Disease
GCP	Good Clinical Practice
GLP	Good Laboratory Practice
GP	glycoprotein
HIV	human immunodeficiency virus
ICF	informed consent form
ICH	International Council for Harmonisation
IDMC	Independent Data Monitoring Committee
IEC	Independent Ethics Committee
IM	intramuscular
IMI	Innovative Medicines Initiative
Inf U	infectious units
IRB	Institutional Review Board
IRE	immediate reportable event
ITN	insecticide treated bed net
IVRS	Interactive Voice Response System
IWRS	Interactive Web Response System
kb	kilobase
MARV	Marburg virus
MedDRA	Medical Dictionary for Regulatory Activities
MoHS	Ministry of Health and Sanitation
MVA	Modified Vaccinia Ankara
MVA-BN	Modified Vaccinia Ankara - Bavarian Nordic
MVA-BN-Filo	Modified Vaccinia Ankara - Bavarian Nordic-multivalent filovirus vector
NGO	non-governmental organization
NHP	nonhuman primate
NP	nucleoprotein
NSAID	nonsteroidal anti-inflammatory drug
PQC	Product Quality Complaint
PRNT	plaque reduction neutralization test
RBC	red blood cell
RNA	ribonucleic acid
PCR	polymerase chain reaction
SAP	Statistical Analysis Plan
SPC	Summary of Product Characteristics
SUDV	Sudan virus
SUSAR	suspected unexpected serious adverse reaction
TAFV	Tai Forest virus

$TCID_{50}$	50% tissue culture infective dose
TOU	test of understanding
UK	United Kingdom
US	United States
VISP	vaccine induced seropositivity
VNA	virus neutralization assay
vp	viral particles
WBC	white blood cell
WHO	World Health Organization

DEFINITIONS OF TERMS

Study vaccine	MVA-BN-Filo or Ad26.ZEBOV or control MenACWY/placebo
Independent study vaccine monitor	An unblinded study vaccine monitor assigned to the study who is responsible for the unblinded interface between the sponsor and the investigational site pharmacy.
Solicited adverse events (reactogenicity)	Local and systemic adverse events that are common and known to occur after vaccination and that are usually collected in a standard, systematic format in vaccine clinical studies. For the list of solicited adverse events in this study, see Section 9.3.2. For the purpose of vaccine clinical studies, all other adverse events are considered unsolicited; however, this definition should be distinguished from definitions based on pharmacovigilance guidelines.

1. INTRODUCTION

Janssen Vaccines & Prevention B.V. (formerly known as Crucell Holland B.V., hereafter referred to as the sponsor), in collaboration with Bavarian Nordic GmbH, Denmark, and in conjunction with the London School of Hygiene & Tropical Medicine and Sierra Leonean MoHS and COMAHS, and the EBODAC program, is investigating the potential of a prophylactic heterologous prime-boost Ebola vaccine regimen that is targeting the EBOV species, which is responsible for the Ebola Virus Disease (EVD) outbreak that started in December 2013 in Guinea and further spread to a number of other countries in West Africa. The vaccine regimen comprises the following 2 candidate Ebola vaccines:

Ad26.ZEBOV (JNJ-61210474-AAA) is a monovalent vaccine expressing the full length Ebola virus (EBOV, formerly known as *Zaire ebolavirus*) Mayinga GP. The vaccine is produced in the human cell line PER.C6®.

MVA-mBN226B (JNJ-63839880-AAA) (further referred to as MVA-BN-Filo®) is a multivalent vaccine expressing the EBOV GP, the SUDV GP, the MARV Musoke GP, and the TAFV NP (formerly known as *Côte d'Ivoire ebolavirus*). The EBOV GP expressed by MVA-BN-Filo has 100% homology to the one expressed by Ad26.ZEBOV.

The monovalent vaccine is part of an ongoing development program for a multivalent vaccine against multiple filoviruses that cause disease in humans, including EBOV, SUDV and MARV. For the most up-to-date nonclinical and clinical information regarding Ad26.ZEBOV and MVA-BN-Filo, refer to the latest versions of the Investigator's Brochures and Addenda (if applicable). A brief summary of the nonclinical and clinical information available at the time of the protocol writing is provided below.[8,9]

The term "sponsor" used throughout this document refers to the entities listed in the Contact Information page(s), which will be provided as a separate document.

1.1. Background

Ebola viruses belong to the *Filoviridae* family and cause EVD, which can induce severe hemorrhagic fever in humans and nonhuman primates (NHPs). Case fatality rates in EVD range from 25% to 90% (average: 50%), according to the World Health Organization (WHO).[14] These viruses are highly prioritized by the United States (US) Government, who has defined them as 'Category A' agents, due to the high mortality rate of infected individuals. Currently, no licensed vaccine, treatment or cure exists for this disease.

Filoviruses are named for their long, filamentous shape. Within this filamentous virus, a single 19-kilobase (kb) negative-sense ribonucleic acid (RNA) genome encodes 7 proteins: the GP, the polymerase, the NP, the secondary matrix protein, the transcriptional activator, the polymerase cofactor, and the matrix protein. The virion surface is covered by homotrimers of the viral GP, which is believed to be the sole host attachment factor for filoviruses. Following cell entry, the viruses replicate their genomes and viral proteins in the cytoplasm using a RNA-dependent RNA polymerase, which is carried into the cell together with the virus.[6]

In this Phase 3 study, the sponsor's adenovirus serotype 26 (Ad26) vector expressing the EBOV Mayinga GP (Ad26.ZEBOV) and the Modified Vaccinia Ankara (MVA) - Bavarian Nordic (BN) vector with EBOV, SUDV and MARV GP inserts and TAFV NP insert (MVA-BN-Filo) will be evaluated as a heterologous prime-boost regimen, in which one study vaccine (Ad26.ZEBOV) is used to prime a filovirus-specific immune response and the other study vaccine (MVA-BN-Filo) is used to boost the immune response 56 days later. The EBOV GP that is currently circulating in West Africa has 97% homology to the EBOV GP used in this vaccine regimen.[1]

Nonclinical Studies

Immunogenicity and Efficacy

Immunogenicity and efficacy of the vaccine combination Ad26.ZEBOV and MVA-BN-Filo was evaluated in an NHP model (ie, Cynomolgus macaques, *Macaca fascicularis*). The combination was assessed in a multivalent filovirus setting in a small number (2 per regimen) of animals and the study included heterologous prime-boost regimens of Ad26, serotype 35 (Ad35) and MVA-BN-Filo vectors expressing different Ebola and Marburg proteins. Full protection from EVD and death after wild-type EBOV Kikwit 1995 challenge was obtained with all heterologous regimens, including the Ad26 and MVA vaccine regimen. All heterologous prime-boost regimens induced comparable immune responses against the EBOV Mayinga GP. Independently of the vaccine regimen, a strong boost effect was seen after heterologous prime-boost immunization.

Two additional studies involving more animals have been performed, to strengthen the robustness of the nonclinical efficacy data, and also to optimize the prime-boost schedule so as to obtain induction of protective immunity as quickly as possible, to specifically respond to the EVD outbreak. Complete survival in animals was observed with 8-week heterologous regimens employing Ad26.ZEBOV as prime and MVA-BN-Filo as boost, whereas a high dose of MVA-BN-Filo in these regimens was associated with complete protection against Ebola virus disease. Partial protection was observed with both MVA-BN-Filo as a prime immunization and a shorter prime/boost interval.

Toxicology

A Good Laboratory Practice (GLP)-compliant, repeated-dose toxicity study was conducted in rabbits with prime-boost combinations of Ad26.ZEBOV and MVA-BN-Filo. The different dose regimens were well tolerated when administered twice by IM injection with a 14-day interval period. All vaccine dosing regimens resulted in detectable ZEBOV GP-specific antibody titers. No significant toxicological effects (no adverse effects) were observed. The immune response was associated with transient increases in C-reactive protein, fibrinogen, and globulin, decreases in hematocrit and hemoglobin, and microscopic findings in draining iliac lymph nodes, spleen and at the injection sites. The findings were noted to be recovering over a 2-week treatment-free period and were considered to reflect a physiological response associated with vaccination. There were no effects noted that were considered to be adverse.

In an embryofetal and pre- and postnatal development study in female rabbits, there was no maternal or developmental toxicity following maternal exposure to the vaccine regimens during

the premating and gestation period. All vaccine regimens elicited detectable EBOV GP-specific maternal antibody titers that were transferred to the fetuses.

Biodistribution

Single-dose biodistribution studies in rabbits were performed using the MVA-BN vector or the Ad26 vector in combination with another insert (Ad26.ENVA.01: an experimental, prophylactic Ad26 vector expressing the human immunodeficiency virus [HIV] type 1, Clade A envelope [Env] protein). MVA-BN distributed to the skin, muscle, blood, spleen, lung, liver, and pooled lymph nodes and was rapidly cleared (within 48 hours following vaccination). Ad26.ENVA.01 was primarily localized in the injection site muscle, the regional lymph nodes and the spleen. Three months after the single IM injection of Ad26.ENVA.01, the vaccine was cleared from most of the examined tissues. As biodistribution is dependent on the vector platform (MVA or Ad26) and not on the insert, it can be assumed that recombinant MVA-BN-Filo or Ad26.ZEBOV is distributed in the same way as the MVA-BN vector or Ad26.ENVA.01 vector, respectively.

Clinical Studies

The safety, tolerability, and immunogenicity of the Ad26.ZEBOV vaccine are being assessed in the ongoing Phase 1 (VAC52150EBL1001, VAC52150EBL1002, VAC52150EBL1003, VAC52150EBL1004 and VAC52150EBL1005), Phase 2 (VAC52150EBL2001) and Phase 3 clinical studies (VAC52150EBL3001, VAC52150EBL3002 and VAC52150EBL3003), where monovalent Ad26.ZEBOV vaccine and multivalent MVA-BN-Filo vaccine are combined in homologous or heterologous prime-boost regimens in which each vector is used to prime a filovirus-specific immune response followed by a boost immunization with the same or the other vector 2 to 12 weeks later. To date, 1,176 healthy adult subjects have been enrolled in these 9 ongoing studies. Two additional Phase 1 studies investigating MVA-BN-Filo are also ongoing (EBL01 and CVD-Mali Ebola Vaccine #1000). Refer to the latest versions of the Ad26.ZEBOV and MVA BN Filo Investigator's Brochures and Addenda (if applicable) for more details.[8,9]

Limited data from the ongoing Phase 1 studies with Ad26.ZEBOV and MVA-BN-Filo are available. To date, no deaths and no other serious adverse events at least possibly related to study vaccine were reported.

VAC52150EBL1001, a first-in-human study, is a randomized, placebo-controlled, observer-blind study in healthy adults evaluating the safety, tolerability and immunogenicity of 4 regimens using MVA BN-Filo at a dose of $1x10^8$ 50% tissue culture infective dose ($TCID_{50}$) and Ad26.ZEBOV at a dose of $5x10^{10}$ viral particles (vp): 2 regimens with MVA BN-Filo as prime and Ad26.ZEBOV as boost at a 28- or 56-day interval, and 2 regimens with Ad26.ZEBOV as prime and MVA-BN-Filo as boost at a 28- or 56-day interval. A fifth regimen, with Ad26.ZEBOV at a dose of $5x10^{10}$ vp as prime, and MVA-BN-Filo at a dose of $1x10^8$ $TCID_{50}$ at a 14-day interval has been evaluated in an open-label, uncontrolled, substudy in healthy adults.

VAC52150EBL1001 has enrolled 87 subjects and primary analysis data for safety and immunogenicity are available for 85 subjects (performed when all subjects had completed their 21-day post-boost visit or discontinued earlier). In both the main study and substudy, no deaths or

adverse events of special interest have been reported. Two subjects in the substudy who experienced grade 3 neutropenia did not receive the boost vaccination as they met criteria for contraindications to administration of the boost (specified in the protocol), but continued scheduled assessments as planned. There were no serious adverse events related to the study vaccines.

In the main study, the most frequent solicited local adverse event was injection site pain. The most common solicited systemic adverse events were fatigue, headache and myalgia.

In the main study, grade 3 solicited local adverse events occurred in 3 subjects (injection site pain and injection site swelling in 1 subject each, injection site erythema in 2 subjects). These events occurred after Ad26.ZEBOV vaccination. Grade 3 solicited systemic events were also reported in 3 subjects (nausea and headache in 2 subjects each, myalgia and fatigue in 1 subject each). All these events occurred after Ad26.ZEBOV, except fatigue which occurred after placebo. The systemic events after Ad26.ZEBOV were considered to be at least possibly related to vaccination by the investigator.

In the substudy, no subjects experienced grade 3 solicited local events. Grade 3 solicited systemic events occurred in 3 subjects after Ad26.ZEBOV (chills and fatigue in 3 subjects each, headache in 2 subjects, pyrexia and nausea in 1 subject each). All were considered to be at least possibly related to vaccination by the investigator. No grade 3 solicited systemic events were reported after MVA BN-Filo.

Overall, the reported adverse events following vaccination were mild in the majority of subjects, transient in nature, and resolved without sequelae. These findings are consistent with the safety profiles observed for similar vaccines. The safety profile of the individual vaccines when used as prime was comparable to that when used as boost.

Both vaccination sequences tested in the main study (ie, Ad26.ZEBOV prime followed by MVA-BN-Filo boost and MVA-BN-Filo prime followed by Ad26.ZEBOV boost) were highly immunogenic and induced considerable humoral and cellular immune responses. Extending the interval between the prime and boost led to increased antibody responses (magnitude at a 56-day prime-boost interval was about 1.8 times higher than at a 28 day prime-boost interval), while the effect was less pronounced or the reverse for T cell responses. The induced immune responses were functional, as demonstrated by the neutralizing activity of the antibody responses in all subjects. The composition of the induced T cell response was favorable, with a high percentage of polyfunctional T cells, which generally are thought to play a role in immunological memory and effector functions.

Study VAC52150EBL1002 completed enrollment of 92 subjects; the blinded phase of the study is ongoing. No serious adverse events related to study vaccine have been reported and no safety issues have been identified to date. Safety data generated with the 2 vaccines with different inserts are provided below.

Safety Profile of Ad26.ZEBOV

Ad26.ZEBOV is a monovalent, replication-incompetent Ad26-based vaccine. Only limited clinical data are available for Ad26.ZEBOV. However, adenovirus vaccine programs with other gene inserts have revealed no significant safety issues.

Safety Profile of MVA-BN

MVA-BN is a further attenuated version of the MVA virus, which in itself is a highly attenuated strain of the poxvirus Chorioallantois Vaccinia Virus Ankara. MVA-BN induces strong cellular activity as well as a humoral (antibody) immune response and has demonstrated an ability to stimulate a response even in individuals with pre-existing immunity against Vaccinia. One of the advantages of MVA-BN is the virus' inability to replicate in a vaccinated individual. The replication cycle is blocked at a very late stage, which ensures that new viruses are not generated and released. This means that the virus cannot spread in the vaccinated person and none of the serious side effects normally associated with replicating Vaccinia viruses have been seen with MVA-BN.

MVA-BN (MVA-BN®, trade name IMVAMUNE® outside the European Union [EU], invented name IMVANEX® in the EU) has received marketing authorization in the EU for active immunization against smallpox in adults and in Canada for persons 18 years of age and older who have a contraindication to the first or second generation smallpox vaccines including individuals with immune deficiencies and skin disorders.[7] A Phase 3 clinical study has been performed in the US (POX-MVA-013). Results of completed and ongoing clinical studies of MVA-BN-based vaccines in more than 8,100 individuals, including elderly subjects (up to age of 80 years), children, and subjects in whom traditional replicating vaccines are contraindicated (eg, individuals with HIV infection or diagnosed with atopic dermatitis), have shown that the platform displays high immunogenicity and a favorable safety profile. Across all clinical studies, no trends for unexpected or serious adverse reactions due to the product were detected.

MVA-BN-Filo is currently also being used in 2 Phase 1 studies in the UK and in Mali (EBL01 and CVD-Mali Ebola Vaccine #1000). In these studies, subjects receive prime vaccination with the monovalent chimpanzee adenovirus vectored cAd3-EBO Z vaccine, followed by MVA-BN-Filo as a booster. No safety concerns have arisen from these studies.

Safety information was pooled from the first 2 studies of MVA-BN-Filo (VAC52150EBL1001 and VAC52150EBL1002). In general, MVA-BN-Filo has been shown to be well tolerated.[9]

Three fifths of the subjects reported at least one local site reaction (injection site pain, tenderness, warmth, redness, swelling and/or itching) following administration of MVA-BN-Filo; mostly of mild severity. The most common reported local site reaction was pain at the injection site. All the local reactions resolved to normal without any lasting effects.

At least one general symptom was reported in two fifths of the subjects following MVA-BN-Filo administration. The most common general symptoms were fatigue, headache, myalgia (muscle pain) and nausea. All general symptoms were transient and resolved without lasting effects.

Changes in laboratory tests were reported following MVA-BN-Filo administration which included hypokalemia and decreased numbers of neutrophils (neutropenia). Both changes in laboratory tests were seen in similar numbers of participants following MVA-BN-Filo and the dummy (placebo) vaccine. Less frequently, events of decreased hemoglobin levels were reported. The changes in laboratory tests were not associated with any complaints or symptoms.

Extensive nonclinical studies support the safety profile of the MVA-BN strain.[11,12]

Relevant Safety Information from Ongoing VAC52150 Studies

One subject in the study VAC52150EBL2001 experienced a serious and very rare condition called “Miller Fisher syndrome”. This condition consists of double vision, pain on moving the eye, and difficulty with balance while walking. Miller Fisher syndrome most commonly occurs following a recent infection. The subject experienced these symptoms about a week after suffering from a common cold and fever. The event happened about a month after boost vaccination with either MVA-BN-Filo or placebo. This subject had to go to the hospital for treatment and has recovered. After an extensive investigation, the event has been considered to be doubtfully related to vaccine and most likely related to the previous common cold.

In the ongoing clinical studies with more than 2,000 participants, there have been a few reports of mild to moderate tingling especially in the hands and feet or a sensation of mild to moderate muscle weakness in subjects vaccinated with Ad26.ZEBOV or placebo. These symptoms have been observed to last no more than 24 to 48 hours in the majority of cases but can last for several weeks before going away on their own. These types of symptoms have also been reported following administration of other licensed vaccines and following acute viral infections of various types. One serious case of probable peripheral sensory neuropathy of moderate severity has occurred and has been ongoing for several months, interfering with some of the subject’s daily activities.

Viral Shedding

Viral shedding information is available from 6 clinical studies with adenovirus vectored vaccines against HIV type 1 (using Ad26 and Ad35: Ad26.ENVA.01 and Ad35.ENV) and *Mycobacterium tuberculosis* (using Ad35: AERAS-402). Viral shedding was not observed in any of these clinical studies. In a clinical study evaluating viral shedding of Ad26.ENVA.01 and Ad35.ENV (Study IPCAVD-004), all cultures from oropharyngeal swabs and urine were negative for adenovirus; in 5 clinical studies evaluating viral shedding of AERAS-402 (Studies C-001-402, C-003-402, C-008-402, C-009-402, C-017-402), no shedding of AERAS-402 was seen in any of the urine or throat cultures.[8]

MVA-BN-Filo is an attenuated recombinant MVA incapable of replication in human cells with a block in the late stage of virus replication. In human cells, upon infection, viral genes are expressed, but no infectious progeny virus is produced. Given the inability of virus assembly and very limited host range of the vector, no viral shedding studies have been performed.

1.2. Risk Benefit Section

1.2.1. Known Benefits

Ad26.ZEBOV and MVA-BN-Filo

The clinical benefit of prime-boost combinations of Ad26.ZEBOV and MVA-BN-Filo is to be established.

MenACWY

To offer clinical benefit to subjects in the control group in Stage 2, an active vaccine MenACWY was chosen instead of placebo. Even when meningitis is diagnosed early and adequate treatment is started, 5% to 10% of patients die, typically within 24 to 48 hours after the onset of symptoms. Left untreated, up to 50% of cases may die. Bacterial meningitis may also result in brain damage, hearing loss or a learning disability in 10% to 20% of survivors.[15,16]

MenACWY is a WHO-prequalified vaccine indicated for active immunization of persons at risk of exposure to *Neisseria meningitidis* serogroups A, C, W135 and Y, to prevent invasive disease.

1.2.2. Potential Benefits

Ad26.ZEBOV and MVA-BN-Filo

Subjects may benefit from clinical testing and physical examination; others may benefit from knowledge gained in this study that may aid in the development of an Ebola vaccine.

If the study vaccines indicate protection, then the participants may benefit from potential protection against EVD.

1.2.3. Known Risks

Ad26.ZEBOV and MVA-BN-Filo

To date, there are only limited data from the Phase 1 studies with Ad26.ZEBOV and MVA-BN-Filo available. However, Ad26- and Ad35-based vaccines with other gene inserts have been administered to a limited number of human volunteers in clinical studies. These other vaccines mainly elicited some local and systemic solicited reactions, as expected with injectable vaccines, and no serious safety concerns were recorded in study participants. Across clinical studies, MVA-BN-based vaccines have been administered to more than 8,100 individuals and no trends for unexpected or serious adverse reactions due to the product were detected. For details, see the safety data presented in Section 1.1.

MenACWY

Very common side effects (affecting ≥1 in 10 people) of MenACWY vaccination include pain, redness and swelling at the injection site, headache, feeling tired, irritable or sleepy, feeling generally unwell, and loss of appetite. For further information on the side effects of the administered vaccine by patient age group, refer to the most recent versions of the applicable vaccine prescribing information.

1.2.4. Potential Risks

The following potential risks for Ad26.ZEBOV and MVA-BN-Filo will be monitored during the study and are specified in the protocol.

Vaccine Induced Seropositivity

The potential of a study participant becoming PCR-positive after vaccination is being assessed in the ongoing Phase 1 US study VAC52150EBL1002. The risk for false positives is low and expected to decrease rapidly over time after administration of Ad26.ZEBOV/MVA-BN-Filo. An account of procedures followed when a PCR test is performed for the evaluation of fever, is given in Section 9.1.6.

In general, uninfected subjects who participate in Ebola vaccine studies may develop Ebola-specific antibodies as a result of an immune response to the candidate Ebola vaccine, referred to as vaccine induced seropositivity (VISP). These antibodies may be detected in Ebola serologic tests, causing the test to appear positive even in the absence of actual Ebola infection. VISP may become evident during the study, or after the study has been completed.

Risk of Myo/Pericarditis

While replicating smallpox vaccines have been associated with an increased risk of developing myo/pericarditis,[10] this has not been observed with MVA-BN and is not expected with this highly attenuated, non-replicating vaccine. Based on observations with first- and second-generation replication-competent smallpox vaccines, particular attention has been placed on the monitoring for cardiac signs and symptoms in all clinical studies using MVA-BN. Despite the close cardiac monitoring, no event indicating a symptomatic case of myo/pericarditis has been observed in any completed MVA-BN study. There has been 1 case of chest pain that might be indicative of pericarditis (consisting of chest pain only with no other cardiac findings suggestive of pericarditis) with previous MVA use, although this diagnosis was not finally confirmed and the patient fully recovered. In a review of prospective surveillances for cardiac adverse events in 6 different clinical studies in 382 subjects receiving MVA vaccines, only 1 subject (0.3%) met the criteria for vaccine-induced myocarditis and eventually the subject was found to suffer from exercise-induced palpitations. Self-limited mild elevations in troponin I were recorded in 3 (0.8%) subjects without evidence of myo/pericarditis.[4] Based on the current exposure data in more than 8,100 subjects vaccinated with MVA-BN and other MVA-BN recombinant products, the safety profile of MVA-BN has shown to be comparable with other licensed, live attenuated vaccines.

Risks Related to Vaccines

Subjects may exhibit general signs and symptoms associated with administration of a vaccine, including fatigue, nausea, headache, vomiting, myalgia, rash, arthralgia, general itching, fever, and chills. In addition, subjects may experience local (injection site) reactions such as redness of skin, swelling/induration, pain/tenderness, and/or itching at the injection site. These events will be monitored, but are generally short-term and do not require treatment.

Fear of injection might result in a vasovagal response, hyperventilation, and in children sometimes in vomiting, breath-holding and rarely convulsions.

Subjects may have an allergic reaction to the vaccination. An allergic reaction may cause a rash, hives or even difficulty breathing (anaphylaxis). Severe reactions are rare. Medications will be available in the clinic or vaccination post to treat severe allergic reactions for subjects in Stage 1 and Stage 2.

Risks from Blood Draws

Blood draws may cause pain, bruising, bleeding, and, rarely, infection at the site where the blood is taken.

Pregnancy and Birth Control

The effect of the vaccines on a fetus or nursing baby is unknown, as well as the effect on semen.

For sexually active female subjects of childbearing potential, it should be confirmed that adequate birth control measures consistent with what is available and the local standards regarding the use of birth control for subjects participating in clinical studies were used from at least 14 days before the prime vaccination with a negative urine β-human chorionic gonadotropin (β-hCG) pregnancy test at screening and immediately prior to each study vaccination (including a late boost vaccination where applicable for subjects affected by a study pause). All male and female subjects, except for female subjects of non-childbearing potential, will be asked to use adequate birth control for sexual intercourse until at least 3 months after the prime vaccination or up to 28 days after the boost vaccination (whichever takes longer). Subjects receiving the third vaccination in Stage 1 will be asked to use adequate birth control for sexual intercourse from at least 14 days prior and until 3 months after the third vaccination.

Pregnancy outcomes from all female subjects who became pregnant during the study until up to 28 days after the boost vaccination or 3 months after the prime vaccination (whichever is longer) as well as from those who became pregnant between at least 14 days before until up to 3 months after the third vaccination may be collected in the current study or in the VAC52150EBL3005 long-term follow-up study for subjects who consent to this.

Children born to these female subjects may be enrolled in the VAC52150EBL3005 study and followed for 5 years if the parent consents to this.

1.2.5. Unknown Risks

There are no clinical data on the use of Ad26.ZEBOV and MVA-BN-Filo in:

- Pediatric subjects (<18 years)
- Pregnant or nursing women
- Elderly subjects (>50 years)
- Immunocompromised patients (including those with HIV infection)

For more information on the unknown risks of MenACWY vaccines, refer to the most recent versions of the applicable vaccine prescribing information.

There may be other serious risks that are not known.

1.2.6. Overall Benefit/Risk Assessment

Based on the available data and proposed safety measures, the overall risk/benefit assessment for this clinical study is considered acceptable for the following reasons:

- Preliminary safety data from the ongoing Phase 1 studies have shown no significant safety concerns to date. Also, safety data generated with the 2 vaccines with different inserts have revealed no significant safety issues (see Section 1.1). Further experience from Ad26.ZEBOV or MVA-BN-Filo will be gained from currently ongoing clinical studies.
- An IDMC will be established to monitor safety data on an ongoing basis to ensure the continuing safety of the subjects enrolled in this study. Details are provided in Section 3.1 and Section 11.7.
- Safety will be closely monitored throughout Stage 1 and Stage 2 of the study. Several safety measures are included in this protocol to minimize the potential risk to subjects, including the following:
  - The safety evaluations in Stage 1 and Stage 2 described in Section 9.3.2. The neuroinflammatory disorders listed in Section 12.1.1 should be categorized as IREs and should be reported to the sponsor as described in Section 12.3.3.
  - The study will commence with vaccination of a group of approximately 40 adult subjects (≥18 years of age) in Stage 1. Approximately 976 subjects, including approximately 400 adults and approximately 576 children ≥1 year of age and adolescents, will be randomized in a 3:1 ratio to receive the Ad26.ZEBOV/MVA-BN-Filo prime-boost regimen or an active control vaccine and placebo in Stage 2. Subjects in Stage 2 will be enrolled in 4 age strata, starting with the eldest age group. A safety review by the IDMC in each of the older age groups will be required before a younger age group can be enrolled. Subjects in Stage 2 will be followed-up for safety until 12 months (children and adolescents) or 24 months (adults) after the prime vaccination. Subjects in Stage 2 will be eligible for roll-over into the Study VAC52150EBL3005 for long-term safety and immunogenicity follow-up up to 60 months post-prime. Subjects in Stage 1 will be followed-up for safety and immunogenicity until 36 months after the prime vaccination, or until 1 year after the third vaccination (if applicable).

1.3. Overall Rationale for the Study

In nonclinical studies in the Cynomolgus macaque model, heterologous prime-boost regimens of a multivalent mixture of Ad26 vectors (each expressing EBOV Mayinga, SUDV or MARV GP) and MVA-BN-Filo provided complete protection against the highly pathogenic wild-type EBOV Kikwit 1995 variant (report pending).

In humans, both Ad26- and MVA-based vaccines containing various antigenic inserts have been shown to be safe and immunogenic (see also Section 1.1). To date, more than 230 subjects have received the sponsor's Ad26-based vaccines in completed clinical studies with Ad26.ZEBOV

(based on the adenoviral vaccine safety database report [dated 20 March 2015]). Up to 28 October 2015, 227 subjects received Ad26.ZEBOV in ongoing studies. The MVA-BN platform is the basis of the non-replicating smallpox vaccine registered in Canada and Europe, and has been safely used in more than 7,600 humans.[9] Furthermore, limited safety data from the ongoing Phase 1 studies have shown no safety concerns to date.

The unprecedented size and scale of the EVD outbreak that started in December 2013 in Guinea and subsequently spread to Sierra Leone and Liberia, led to the outbreak being declared a public health emergency of international concern in August 2014 by the WHO. This study is one of a series of studies to evaluate the heterologous combination of Ad26.ZEBOV and MVA-BN-Filo as a possible vaccine regimen to prevent EVD.

In this Phase 3 study, Ad26.ZEBOV and MVA-BN-Filo will be evaluated as a heterologous prime-boost regimen, in which one study vaccine (Ad26.ZEBOV) is used to prime a filovirus-specific immune response and the other study vaccine (MVA-BN-Filo) is used to boost the immune response 56 days later, to provide long-lasting protection against infection with EVD. In Stage 1, a third vaccination (Ad26.ZEBOV) will be administered at least 2 years post prime. The EBOV GP that is currently circulating in West Africa has 97% homology to the EBOV GP used in this vaccine regimen.

The MVA-BN-Filo dose that will be used in the present study is expressed as $1x10^8$ Inf U. This dose corresponds to the dose of $1x10^8$ $TCID_{50}$ being used in the current Phase 1 studies.

The study protocol will be implemented in conjunction with the London School of Hygiene & Tropical Medicine, the MoHS, the COMAHS, and the EBODAC program. The MoHS and COMAHS will partner the London School of Hygiene & Tropical Medicine in all aspects of the implementation of the study, and particularly with regard to recruitment of clinical and para-medical staff, training, clinical management, and surveillance. The MoHS will also provide referral facilities for all stages of the study. The study surveillance staff will work closely with district and national surveillance system to monitor EVD cases and background morbidity and mortality. GOAL is supporting the study in logistics, construction, supply and recruitment of non-clinical staff.

2. OBJECTIVES AND HYPOTHESIS

2.1. Objectives

The primary objective of Stage 1 is:

- To evaluate the safety of a heterologous prime-boost regimen utilizing Ad26.ZEBOV and MVA-BN-Filo given at a 56-day interval.

The secondary objective of Stage 1 is:

- To assess humoral immune responses to a heterologous prime-boost regimen utilizing Ad26.ZEBOV and MVA-BN-Filo as measured by an enzyme-linked immunosorbent assay (ELISA) at 21 days post-boost vaccination.

- To assess the safety and tolerability of a third vaccination using Ad26.ZEBOV administered at least 2 years post prime.

The exploratory objectives of Stage 1 are:

- To assess humoral immune responses to a heterologous prime-boost regimen utilizing Ad26.ZEBOV and MVA-BN-Filo as measured by ELISA at other relevant time points.
- To assess neutralizing antibody responses directed against EBOV GP induced by the heterologous prime-boost regimen as measured by a virus neutralization assay (VNA) depending on sample and assay availability.
- To assess antibody responses directed against the Ad26 and/or MVA vector as measured by ELISA, VNA, and/or plaque reduction neutralization test (PRNT) depending on sample and assay availability.
- To assess humoral immune responses to a heterologous prime-boost regimen utilizing Ad26.ZEBOV and MVA-BN-Filo as measured by ELISA following a third vaccination using Ad26.ZEBOV at 2 years post prime.

The primary objective of Stage 2 is:

- To evaluate the safety of a heterologous prime-boost regimen utilizing Ad26.ZEBOV and MVA-BN-Filo given at a 56-day interval compared to an active control vaccine.

The secondary objective of Stage 2 is:

- To assess humoral immune responses to a heterologous prime-boost regimen utilizing Ad26.ZEBOV and MVA-BN-Filo as measured by an ELISA at 21 days post-boost vaccination.

The exploratory objectives of Stage 2 are:

- To assess humoral immune responses to a heterologous prime-boost regimen utilizing Ad26.ZEBOV and MVA-BN-Filo as measured by ELISA at other relevant time points.
- To assess neutralizing antibody responses directed against EBOV GP induced by the heterologous prime-boost regimen as measured by VNA depending on sample and assay availability.
- To assess antibody responses directed against the Ad26 and/or MVA vector as measured by ELISA, VNA, and/or PRNT depending on sample and assay availability.

2.2. Hypothesis

As this study is designed to provide descriptive information regarding safety and immunogenicity without formal treatment comparisons, no formal statistical hypothesis testing is planned.

3. STUDY DESIGN AND RATIONALE

3.1. Overview of Study Design

This is a staged Phase 3 study with an open-label uncontrolled stage (Stage 1) and a double-blinded controlled stage (Stage 2) to evaluate the immunogenicity and safety of a heterologous prime-boost regimen where Ad26.ZEBOV at a dose of $5x10^{10}$ vp will be used to prime a filovirus-specific

immune response and MVA-BN-Filo at a dose of $1x10^8$ Inf U will be used to boost the immune response 56 days later. In Stage 1, a third vaccination using Ad26.ZEBOV will be administered at least 2 years post prime to subjects who consent to this.

The study will be conducted as follows:

- Stage 1: The study will commence with vaccination of a group of approximately 40 adult subjects aged 18 years or older. The objective of this initial stage of the study is to evaluate the safety and immunogenicity of the prime-boost regimen in the adult Sierra Leonean population.
- Stage 2: Approximately 976 subjects aged 1 year or older will be individually randomized in a 3:1 ratio to receive the Ad26.ZEBOV/MVA-BN-Filo prime-boost regimen or an active control vaccine and placebo. The aim is to enroll approximately 400 adults (aged 18 years or older) and approximately 576 children aged ≥1 year (with about 192 children in each of the 3 age groups [ie, 12 to 17 years, 4 to 11 years, and 1 to 3 years, inclusive]). Enrollment will be staggered, starting with the eldest age group. The decision to proceed to the next age group will be based on an evaluation by the IDMC (for details see Section 11.7). Randomization will be stratified by age group.

Within each age group of Stage 2, study-site personnel (except for those with primary responsibility for study vaccine preparation and dispensing), sponsor personnel and subjects will be blinded to the study vaccine until all subjects in that age group have completed at least the 6 month post-boost visit or discontinued earlier and the database has been locked for that age group. Should an interim analysis be performed prior to the 6 months post-boost visit of the last subject in a given age group, only those involved in the analysis and decision making will be unblinded.

An IDMC will be commissioned for this study. The IDMC will review all available 7-day post-prime safety data (including clinical laboratory results) before the first subject of the current cohort receives the boost:

In Stage 1 to determine whether:

  - the boost vaccination in Stage 1 can be initiated, and
  - the prime vaccination in Stage 2 (subjects aged 12 to 17 years inclusive) can be initiated.

- In Stage 2 (subjects aged 12 to 17 years inclusive) to determine whether:
  - the boost vaccination of subjects aged 12 to 17 years inclusive can be initiated, and
  - the prime vaccination of subjects aged 4 to 11 years inclusive can be initiated.
- In Stage 2 (subjects aged 4 to 11 years inclusive) to determine whether:
  - the boost vaccination of subjects aged 4 to 11 years inclusive can be initiated, and
  - the prime vaccination of subjects aged 1 to 3 years inclusive can be initiated.
- In Stage 2 (subjects aged 1 to 3 years inclusive) to determine whether:
  - the boost vaccination of subjects aged 1 to 3 years inclusive can be initiated.

The IDMC will also review all cumulative safety data including the 7-day post-boost safety data (including clinical laboratory results) to determine if the boost vaccination in the next stage and/or next age group can be given. An IDMC meeting will therefore take place with all available data (also including the 7-day post-prime safety data within that age group) before boost vaccination will be administered.

A schematic presentation of the IDMC schedule is provided in Attachment 1.

The IDMC will also assess on an ongoing basis the Stage 1 and Stage 2 data as well as data emerging from Phase 1 and 2 studies and the status of the epidemic.

While Stage 2 of this study was enrolling, the sponsor halted all vaccinations in the clinical program due to the occurrence of a serious and very rare condition, Miller Fisher syndrome, reported in study VAC52150EBL2001, as well as following reports of mild transient paresthesia in the same study, until a revised ICF was prepared and approval to restart the current study was granted by all relevant authorities. This interruption in dosing affected 98 consented subjects in the current study, who had only received the prime vaccination. When approval was granted to restart the study under Amendment 3, a late boost vaccination was offered to those subjects who had only received the prime vaccination, unless participants had withdrawn from the study or were not eligible to receive the boost. Vaccinated subjects will follow the same post-boost vaccination schedule as those subjects unaffected by the pause. Subjects who refuse or are not eligible to receive the late boost will be encouraged to remain in the study to be followed for safety and immunogenicity.

As part of Amendment 4 for this study, Stage 1 was extended for 2 years beyond Day 360 post prime for follow-up of safety and immunogenicity. As part of Amendment 5, subjects in Stage 1 who consent to this will receive a third vaccination (Ad26.ZEBOV) at least 2 years post prime vaccination. The follow-up period for adult subjects in Stage 2 will be extended for up to 2 years post prime, for those subjects who consent to this.

Female subjects in Stage 1 of the study who became pregnant with estimated conception within 28 days after vaccination with MVA-BN-Filo or within 3 months after vaccination with Ad26.ZEBOV, children born to vaccinated female subjects in Stage 1 who became pregnant with estimated conception within 28 days after vaccination with MVA-BN-Filo or within 3 months after vaccination with Ad26.ZEBOV (unless local regulations have additional requirements for follow up) and subjects enrolled in Stage 2 of the study (including female subjects who became pregnant and children born to vaccinated female subjects who became pregnant as specified above for Stage 1) may be invited for enrollment into the VAC52150EBL3005 study for long-term follow-up of safety and immunogenicity for a total of up to 60 months after the prime vaccination. Roll-over of subjects in Stage 2 of the study will occur after unblinding of VAC52150EBL3001 and will only include subjects who received Ad26.ZEBOV and/or MVA-BN-Filo.

Immunogenicity Assessments

Immunogenicity assessments for humoral immune responses will be performed in all subjects (adults, adolescents, and children aged 1 year or older) from Stage 1 and Stage 2. Blood samples

will be collected as specified in the Time and Events Schedules. The amount of blood taken from children will be limited (see Section 9.1.1). Subjects from Stage 1 and Stage 2 will be followed-up for immunogenicity until 36 months post prime or 1-year post-third vaccination (if applicable) in Stage 1, until 1 year post prime for children and adolescents in Stage 2 and until 1 year post prime or 2 years post prime (if applicable) for adults in Stage 2.

3.2. Study Design Rationale

The primary endpoint of Stage 1 and Stage 2 is safety and the secondary endpoint of Stage 1 and Stage 2 is immunogenicity.

Stage 1

Stage 1 of the study is designed to evaluate the prime-boost regimen (Ad26.ZEBOV/MVA-BN-Filo/Ad26.ZEBOV) in the adult Sierra Leonean population.

Immunogenicity data from a Phase 1 study of the monovalent vaccine program (VAC52150EBL1002) demonstrated stability of the binding antibody response out to one year following prime-boost regimens with Ad26.ZEBOV/MVA-BN-Filo (or the reverse). A third vaccination using Ad26.ZEBOV at $5x10^{10}$ vp was given at the one-year time point leading to a marked and rapid increase in the binding antibody responses within 7 days (at least 10-fold). The marked and rapid rise was generally independent of the antecedent prime-boost regimen. The profile of the antibody response strongly suggests that robust anamnestic responses can be induced after re-exposure to an EBOV antigen, in this case mimicked by a third vaccination. Administration of a third vaccination using Ad26.ZEBOV at least 2 year post prime for subjects in Stage 1 will extend the findings to an African population.

Stage 2

Stage 2 will evaluate the prime-boost regimen across different age groups including children, using age de-escalation. Stage 2 will be an individually randomized, double-blinded, active-controlled study. Randomization will be used in Stage 2 to minimize bias in the assignment of subjects to study arms (Ad26.ZEBOV/MVA-BN-Filo prime-boost regimen or control), to increase the likelihood that known and unknown subject attributes (eg, demographic and baseline characteristics) are evenly balanced across arms, and to enhance the validity of possible statistical comparisons across arms.

Control recipients are included for blinding purposes and safety analyses, and will provide control specimens for immunogenicity assays. The nature of the study vaccine (Ad26.ZEBOV/MVA-BN-Filo prime-boost regimen versus control) will be blinded to reduce potential bias during data collection and evaluation of clinical safety endpoints. Blinding will be guaranteed by preparation of study vaccine by unblinded qualified study-site personnel not involved in any other study-related procedures, and by the administration of vaccine in a masked syringe in a way that maintains double-blinding.

4. SUBJECT POPULATION

The study population will consist of:

- Stage 1: approximately 40 healthy subjects aged 18 years or older.
- Stage 2: approximately 976 healthy subjects aged 1 year or older. The aim is to enroll approximately 400 adults (aged 18 years or older) and approximately 576 children aged ≥1 year (192 adolescents aged 12 to 17 years inclusive, 192 children aged 4 to 11 years inclusive, and 192 young children aged 1 to 3 years inclusive). Enrollment will be staggered, starting with the eldest age group. The decision to proceed to the next age group will be based on an evaluation by the IDMC.

HIV-positive subjects can be enrolled as long as their general condition is good, ie, they are on antiretroviral treatment or have no signs or symptoms of immune incompetence, diagnosed on the basis of physical examination, medical history, and the investigator's clinical judgment.

Community engagement will be sought in all stages.

Subsequently, subjects will be asked to provide individual consent to receive the vaccine regimen and for collecting safety data under the provisions of this protocol. The in- and exclusion criteria are described in the following 2 subsections.

If there is a question about the inclusion or exclusion criteria below, the investigator or designee must consult with the appropriate sponsor representative and resolve any issues before enrolling a subject in the study. Waivers are not allowed.

For a discussion of the statistical considerations of subject selection, refer to Section 11.3.

4.1. Inclusion Criteria

Each potential subject must satisfy all of the following criteria to be enrolled in the study.

Stage 1

1. Documented community engagement by a community leader must be available. At the individual level, each subject must sign an ICF indicating that he or she understands the purpose of and procedures required for the study and is willing to participate in the study. In case the subject cannot read or write, the procedures must be explained and informed consent must be witnessed by a literate third party not involved in the conduct of the study.
2. Must be a male or female subject aged 18 years or older at screening.
3. Must be resident in the selected study community, with no intention to move from the study area within the next 5 months.
4. Must be healthy on the basis of physical examination and clinical laboratory tests performed at screening.
   Subjects must meet the following laboratory criteria within 28 days before Day 1:
   - Hemoglobin: women: ≥12.5 g/dL; men ≥13.0 g/dL
   - Hematocrit: ≥36%

– Red blood cell (RBC) count: ≥4,500,000/mm^3
– White blood cell (WBC) count: ≥4,000/mm^3
– Platelets: ≥150,000/mm^3
– Alanine aminotransferase (ALT): ≤69 U/L
– Aspartate aminotransferase (AST): ≤46 U/L
– Creatinine: <109 µmol/L

Note: If laboratory screening tests are out of range, repeat of screening tests is permitted once using an unscheduled visit during the screening period to assess eligibility.

5. Female subjects of childbearing potential must use adequate birth control measures consistent with local regulations regarding the use of birth control for subjects participating in clinical studies from at least 14 days before the prime vaccination, with a negative urine β-hCG pregnancy test at screening and immediately prior to the prime vaccination, which shall occur no earlier than 14 days after the screening visit.
6. Male subjects who are sexually active with a woman of childbearing potential must be willing to use condoms for sexual intercourse beginning prior to enrollment.
7. Must pass the test of understanding (TOU).

   Note: The TOU will be administered after reading but before signing the ICF. If a subject fails the TOU on the first attempt, the subject must be retrained on the purpose of the clinical study and must take the test again. A subject who fails the TOU cannot participate in the study.

Subjects who fail screening in Stage 1 are allowed to be assessed again for eligibility to receive study vaccine in Stage 2.

Stage 2

8. Criterion modified per Amendment 5

   1.1 Documented community engagement by a community leader must be available. At the individual level, adult subjects (aged 18 years and older at screening) must sign an ICF indicating that he or she understands the purpose of the study and the procedures required for the study and is willing to participate in the study. In case the subject cannot read or write, the procedures must be explained and informed consent must be witnessed by a literate third party not involved in the conduct of the study. In case the subject’s age is below the legal cut-off age for consent (according to local regulations), the parent/legal guardian will be asked to give consent, and the subject will be informed about the study. Subjects aged 7 years and older will be asked to give positive assent. The assent procedure must be witnessed by an adult literate parent/legal guardian/third party not involved in the conduct of the study, and documented.
9. Must be a male or female subject aged 1 year or older at screening.

   Note: Children of parents enrolled in this stage are eligible.
10. Must be resident in the selected study community, with no intention to move from the study area during the subject's participation in the study.
11. Criterion modified per Amendment 5

4.1 Must be healthy on the basis of physical examination and clinical laboratory tests performed at screening. If the results of the laboratory screening tests are outside the institutional normal reference ranges, the subject may be included only if the investigator judges the abnormalities or deviations from normal to be not clinically significant or to be appropriate and reasonable for the population under study. This determination must be recorded in the subject's source documents and initialed by the investigator.

Note: *The safety laboratory assessments at screening are to be performed within 28 days prior to the prime vaccination on Day 1 (including Day 1 before vaccination) and may be repeated if they fall outside this time window.*

Note: *If laboratory screening tests are out of range and deemed clinically significant, repeat of screening tests is permitted once using an unscheduled visit during the screening period to assess eligibility.*

12. Criterion modified per Amendment 4.

5.1 Sexually active female subjects of childbearing potential must use adequate birth control measures consistent with the local standards regarding the use of birth control for subjects participating in clinical studies and what is available from at least 14 days before the prime vaccination, with a negative urine β-hCG pregnancy test at screening and immediately prior to the prime vaccination, which shall occur no earlier than 14 days after the screening visit.

13. Male subjects who are sexually active with a woman of childbearing potential must be willing to use condoms for sexual intercourse beginning prior to enrollment.

14. Subjects or the parent/legal guardian (for children) must pass the TOU.

Note: *The TOU will be administered after reading but before signing the ICF. If a subject or the parent/legal guardian (for children) fails the TOU on the first attempt, the subject or parent/legal guardian must be retrained on the purpose of the clinical study and must take the test again (2 re-takes are allowed). A subject who fails the TOU cannot participate in the study.*

4.2. Exclusion Criteria

Any potential subject who meets any of the following criteria will be excluded from participating in the study.

Stage 1

1. Women who are breast-feeding or known to be pregnant. A pregnancy test will be performed at screening in women of childbearing potential.

2. Subjects who have an illness prior to vaccination. This does not include acute illnesses such as diarrhea or mild upper respiratory tract infection unless accompanied by body temperature >37.5°C.

3. Subjects with any clinically significant acute or chronic medical condition that in the opinion of the investigator would preclude participation (eg, history of seizure disorders, bleeding/clotting disorder, cardiac disease, autoimmune disease, active malignancy, poorly controlled asthma, active tuberculosis, or other systemic infections). Subjects who identify

themselves as being HIV-positive can be included as long as their general condition is good, ie, they are on antiretroviral treatment or have no signs or symptoms of immune incompetence.

4. Subjects with anemia (defined as hemoglobin <10 g/dL).
5. Subjects who have had a previous adverse reaction to vaccination excluding mild non-systemic adverse reactions.
6. Subjects who are being treated with an immunosuppressive drug at the time of screening.
7. Subjects who have received a blood transfusion or other blood products within 8 weeks of vaccination.
8. Subjects who have been vaccinated with a candidate Ebola vaccine or have participated in another Ebola preventative or therapeutic biomedical intervention study (participation in observational or behavioral studies is allowed).
9. Subjects who have been vaccinated with live-attenuated vaccines within 30 days before the prime vaccination, and with inactivated vaccines within 15 days before the prime vaccination.
10. Subjects who are known to have received any Ad26- or MVA-based candidate vaccine in the past.
11. Subjects who have been diagnosed with EVD or are under quarantine/have been exposed to Ebola.
12. Subjects who, in the opinion of the investigator, are unlikely to adhere to the requirements of the study or are unlikely to complete the full course of vaccination and observation.
13. Fever (body temperature ≥38.0°C). Temperature will be taken according to standardized temperature monitoring as per Ebola response regulations. Subjects with a fever of ≥38.0°C will be referred to the EVD assessment services.

Subjects who fail screening in Stage 1 are allowed to be assessed again for eligibility to receive study vaccine in Stage 2.

Stage 2

1. Women who are breast-feeding or known to be pregnant. A pregnancy test will be performed at screening in women of childbearing potential.
2. Subjects who have an illness prior to vaccination. This does not include acute illnesses such as diarrhea or mild upper respiratory tract infection unless accompanied by body temperature ≥38.0°C.
3. Subjects with any clinically significant acute or chronic medical condition that in the opinion of the investigator would preclude participation (eg, history of seizure disorders, bleeding/clotting disorder, cardiac disease, autoimmune disease, active malignancy, poorly controlled asthma, active tuberculosis, or other systemic infections). Subjects who identify themselves (or are identified by a parent/legal guardian in case of children) as being HIV-positive can be included as long as their general condition is good, ie, they are on antiretroviral treatment or have no signs or symptoms of immune incompetence, diagnosed on the basis of physical examination, medical history, and the investigator’s clinical judgment.
4. Subjects with anemia (defined as hemoglobin <8 g/dL for children aged ≥1 to <5 years, <9 g/dL for children aged ≥5 to <15 years, and <10 g/dL for subjects aged ≥15 years).

5. Subjects who have had a previous adverse reaction to vaccination excluding mild non-systemic adverse reactions.[a]
6. Subjects who are being treated with an immunosuppressive drug at the time of screening.
7. Subjects who have received a blood transfusion or other blood products within 8 weeks of vaccination.
8. Subjects who have been vaccinated with a candidate Ebola vaccine or have participated in another Ebola preventative or therapeutic biomedical intervention study (participation in observational or behavioral studies is allowed).
9. Subjects who have been vaccinated with live-attenuated vaccines within 30 days before the prime vaccination, and with inactivated vaccines within 15 days before the prime vaccination.
10. Subjects who are known to have received any Ad26- or MVA-based candidate vaccine in the past.
11. Subjects who have been diagnosed with EVD or are under quarantine/have been exposed to Ebola.
12. Subjects who, in the opinion of the investigator, are unlikely to adhere to the requirements of the study or are unlikely to complete the full course of vaccination and observation.
13. Criterion modified per Amendment 5

    13.1 Children with weight-per-height below 10th percentile according to the WHO (1- and 2-year-olds) and Centers for Disease Control and Prevention (CDC) growth charts (3- to 11-year-olds).[2,13]

14. Fever (body temperature ≥38.0°C). Temperature will be taken according to standardized temperature monitoring as per Ebola local response regulations. Subjects with a fever of ≥38.0°C will be referred to routine medical follow-up (including the EVD assessment services, if applicable).
15. Subjects who have received a meningitis vaccine in the past.

NOTE: Investigators should ensure that all study enrollment criteria have been met prior to the prime vaccination on Day 1. If a subject's clinical status changes (including receipt of additional medical records or available laboratory results in Stages 1 and 2) after screening but before the prime vaccination is given such that he or she no longer meets all eligibility criteria, then the subject will be excluded from participation in the study. Section 17.4 describes the required documentation to support meeting the enrollment criteria.

[a] Known allergy or history of anaphylaxis or other serious adverse reactions to vaccines or vaccine products (including any of the constituents of the study vaccines [eg, polysorbate 80, ethylenediaminetetraacetic acid (EDTA) or L-histidine for Ad26.ZEBOV vaccine; tris (hydroxymethyl)-amino methane (THAM) for MVA-BN-Filo vaccine], including known allergy to egg, egg products and aminoglycosides.

4.3. Prohibitions and Restrictions

Potential subjects must be willing and able to adhere to the following prohibitions and restrictions during the course of the study to be eligible for participation:

1. Criterion modified per Amendment 5

   1.1 Subjects enrolled in Stage 1 should be willing to reside in the study area for 5 months after the prime vaccination. Subjects in Stage 1 who consent to the third vaccination must be willing to reside in the study area for at least 6 months post-third vaccination. Subjects enrolled in Stage 2 should be willing to reside in the study area throughout the subject's participation to the study. Appropriate measures will be installed to uniquely identify subjects in order to ensure correct application of the prime-boost regimen.

2 Criterion modified per Amendment 5

   2.1 For sexually active female subjects of childbearing potential, it should be confirmed that adequate birth control measures consistent with what is available and the local standards regarding the use of birth control for subjects participating in clinical studies were used from at least 14 days before the prime vaccination with a negative urine β-hCG pregnancy test at screening and a negative urine β-hCG pregnancy test immediately prior to each study vaccination. All male and female subjects, except for female subjects of non-childbearing potential, will be asked to use adequate birth control for sexual intercourse until at least 3 months after the prime vaccination or up to 28 days after the boost vaccination (whichever takes longer). Subjects receiving the third vaccination in Stage 1 will be asked to use adequate birth control for sexual intercourse from at least 14 days prior and until 3 months after the third vaccination.

3. Analgesic/antipyretic medications and nonsteroidal anti-inflammatory drugs (NSAIDs) may be used post-vaccination only in case of medical need (eg, body temperature ≥38.0°C or pain) and for subjects in Stage 1 and Stage 2, their use must be documented. If the temperature does not abate or is not controlled after 24 hours, the person will be instructed to contact the study personnel and/or consult the nearest health care unit to her/his household/section. Use of these medications as routine prophylaxis prior to study vaccine administration is discouraged. For details, refer to Section 9.1.6.

4. Criterion modified per Amendment 5

   4.1 Criterion modified per Amendment 6

   4.2 Subjects should not receive live-attenuated vaccines from 30 days before the prime vaccination until 30 days after the boost vaccination and from 30 days before until 30 days after the third vaccination in Stage 1, and inactivated vaccines from 15 days before or after administration of prime and boost vaccination and 15 days before or after third vaccination in Stage 1. The possibility exists that a vaccine preventable disease such as measles can start circulating and causing outbreaks or an epidemic during the course of the study. If this happens, receipt of live-attenuated vaccines such as measles vaccine will be allowed but will be documented.

5. Vaccination posts and clinics will implement standard Ebola prevention methods such as provision of handwashing stations and temperature checks and all vaccinees and staff will follow these recommendations during scheduled and unscheduled visits.

5. TREATMENT ALLOCATION AND BLINDING

Community Engagement

This study will build on the community engagement and community capacity established in collaboration/coordination with non-governmental organizations (NGOs) and institutions performing social mobilization and contact tracing for Ebola control.

Direct community engagement for this study will begin as early as possible and MoHS, UNICEF, UNMEER, WHO and other government authorities will be consulted for their input. Guidance will also be sought from groups currently conducting social mobilization for the Ebola response.

A key aspect of the initial, community fact-finding, research work during the period prior to the study active phase of implementation (vaccination) will be a stakeholder analysis, results of which will be used to identify relevant stakeholders to be considered in community engagement such as paramount chiefs, sub-chiefs, mesquites (imams), churches, schools, law enforcement, government structures at community level, health-related committees, and development-related committees. One of the main tasks of the study staff will be to keep dialogue open and ongoing between the study researchers, the MoHS and the community at large.

Community engagement will be an ongoing process through regular contacts with community groups and designated study team members. A combination of mechanisms will be utilized, such as community meetings, workshops with key stakeholders, participant meetings, and others. Community advisory boards, comprising key influential figures with the community, together with members of the local population, will be established and continually engaged to provide a dialogue between the community and study team and allow the community to provide feedback on any concerns that they may have. Community engagement will also allow researchers to receive feedback from the community on social harms, individual and community level risks, perceptions about the study, and vaccination implementation challenges. All study staff and stakeholders will receive training in Good Clinical Practice (GCP)/research ethics before commencement of the

intervention (Ebola vaccination). The paramount chief for the relevant study area will be asked to sign an agreement form to agree the study to take place within his community.

Community engagement will also be a component of other study processes such as the communication plan, especially the dissemination of study results to the community. Overall, strong community engagement will allow the establishment of a partnership between communities, participants and researchers to ensure the latter discharge their responsibilities ethically in the study communities.

The selected study area has a population of several thousand people. To be able to handle demand and expectations, the following process will take place as a part of community engagement:

- Numbering of all the households in the community (this has already been done by the national authorities).
- Conducting an open lottery, presided over by community leaders, at which these numbered households are drawn by lot and listed in sequential order on a formal document which will be signed off by the community leaders.
- Households will then be visited in order and asked if they would like to join the study; if the household declines, the next one on the list will be approached and asked to join, the process being continued until a sufficient number have been invited for the study.

Community members who became aware of the study through other community engagement activities but were not part of the invitation process, and decide to volunteer for participation at the clinic, will not be rejected. However, priority will be given to those responding to the invitation process.

Sampling/Recruitment of Population

Appropriate measures will be installed to uniquely identify subjects in order to ensure correct application of the prime-boost regimen.

Randomization

There will be no randomization in Stage 1. Assignment of the third vaccination in Stage 1 will be done using Interactive Voice/Web Response System (IVRS/IWRS)

The randomization through IVRS/IWRS in Stage 2 will be 3:1 (Ad26.ZEBOV/MVA-BN-Filo:active control) and will be stratified by age group.

Blinding

Blinding procedures are not applicable for Stage 1.

Within each age group of Stage 2, study-site personnel (except for those with primary responsibility for study vaccine preparation and dispensing), sponsor personnel (except for specifically designated sponsor personnel who are independent of the study) and subjects will be blinded to the study vaccine allocation until all subjects have completed at least the 6 month post-boost visit or discontinued earlier and the database has been locked for that age group.

The investigator will not be provided with randomization codes. The codes will be maintained within the IVRS/IWRS, which has the functionality to allow the investigator to break the blind for an individual subject.

Data that may potentially unblind the study vaccine allocation (ie, study vaccine preparation/accountability data, or other specific laboratory data) will be handled with special care to ensure that the integrity of the blind is maintained and the potential for bias is minimized. This will include making special provisions, such as segregating the data in question from view by the investigators, clinical team, or others as appropriate, until the time of database lock and unblinding. The pharmacy and preparation of study vaccines will be monitored by an independent study vaccine monitor (see Section 17.8).

Under normal circumstances, the blind of each age group in Stage 2 should not be broken until the last subject in the age group has completed at least the 6 month post-boost visit or discontinued earlier and the database for the respective age group has been locked. Otherwise, the blind should be broken only if a specific emergency treatment/course of action would be dictated by knowing the treatment status of the subject. In such cases, the investigator may, in an emergency, determine the identity of the treatment by contacting the IVRS/IWRS. It is recommended that the investigator contact the sponsor or its designee if possible to discuss the particular situation, before breaking the blind. Telephone contact with the sponsor or its designee will be available 24 hours per day, 7 days per week. In the event the blind is broken, the sponsor must be informed as soon as possible. The date and reason for the unblinding must be documented by the IVRS/IWRS, in the appropriate section of the Case Report Form (CRF), and in the source document. The documentation received from the IVRS/IWRS indicating the code break must be retained with the subject's source documents in a secure manner.

When the last subject in a given age group has completed at least the 6-month post-boost visit or discontinued earlier and the database for that age group has been locked, randomization codes may be disclosed for that age group. Should an interim analysis be performed prior to the 6 months post-boost visit of the last subject in a given age group, only those involved in the analysis and decision making will be unblinded.

If the randomization code is broken by the investigator or the study-site personnel, the subject must discontinue further study vaccine administration and must be followed as appropriate (see Section 10.2 for details). If the randomization code is broken by the sponsor for safety reporting purposes, the subject should not discontinue further study vaccine administration and may remain in the study (if the randomization code is still blinded to the study-site personnel and the subject).

6. DOSAGE AND ADMINISTRATION

All subjects in Stage 1 and all subjects randomized to the Ad26.ZEBOV/MVA-BN-Filo arms in Stage 2 will receive the following as a 0.5 mL IM injection into either deltoid in the upper arm (or, if needed, in the thigh):

- Ad26.ZEBOV: $5x10^{10}$ vp on Day 1
- MVA-BN-Filo: $1x10^{8}$ Inf U on Day 57

Subjects in Stage 1 who consent to this will receive a third vaccination using Ad26.ZEBOV at $5x10^{10}$ vp at least 2 years post-prime vaccination.

Subjects in the control arm of Stage 2 will receive the WHO-prequalified MenACWY vaccine as prime on Day 1 and placebo as boost on Day 57. The MenACWY vaccine will be administered as an IM injection into either deltoid in the upper arm. For details, refer to Section 14.1.3.

Children aged less than 2 years at randomization will receive a third vaccination at 3 months post boost with either placebo (Ad26.ZEBOV/MVA-BN-Filo arm) or MenACWY (control arm).

The vaccination schedule is presented in Table 2.

Table 2: Study Vaccination Schedules

N (per group)	Prime (D1)	Boost (D57)	Third vaccination (2 years post prime [Stage 1] 3 months post boost [Stage 2]) [a)]
**Stage 1**			
Adults (≥18 years)			
40	Ad26.ZEBOV $5x10^{10}$ vp	MVA-BN-Filo $1x10^{8}$ Inf U	Ad26.ZEBOV $5x10^{10}$ vp
**Stage 2**			
Adults (≥18 years)			
300	Ad26.ZEBOV $5x10^{10}$ vp	MVA-BN-Filo $1x10^{8}$ Inf U	NA
100	MenACWY	placebo	NA
Adolescents (12-17 years inclusive)			
144	Ad26.ZEBOV $5x10^{10}$ vp	MVA-BN-Filo $1x10^{8}$ Inf U	NA
48	MenACWY	placebo	NA
Children (4-11 years inclusive)			
144	Ad26.ZEBOV $5x10^{10}$ vp	MVA-BN-Filo $1x10^{8}$ Inf U	NA
48	MenACWY	placebo	NA
Young children (1-3 years inclusive)			
144	Ad26.ZEBOV $5x10^{10}$ vp	MVA-BN-Filo $1x10^{8}$ Inf U	Placebo (<2 years)
48	MenACWY	placebo	MenACWY (<2 years)

D: day; Inf U: infectious units; N: number of subjects per group to receive study vaccine; NA: not applicable; vp: viral particles.
[a)] The third vaccination in Stage 2 is only applicable for children aged less than 2 years at randomization.

Study vaccines in Stage 2 will be prepared by an unblinded pharmacist or qualified staff member with primary responsibility for study vaccine preparation and dispensing and who is not involved in any other study-related procedures. Subjects will be administered the study vaccines in a masked syringe in a way that maintains double-blinding.

Upon completion of the study, sites may offer MenACWY to the Ad26.ZEBOV/MVA-BN-Filo arm and the Ad26.ZEBOV/MVA-BN-Filo vaccine (if licensed and/or WHO-prequalified) to the control arm upon consultation with the health authorities.

When choosing an arm for the injection, it should be considered whether there is an arm injury, local skin problem, and/or significant tattoo that preclude administering the injection or will interfere with evaluating the arm after injection.

For subjects in Stage 1 and Stage 2, it is recommended that the boost vaccination will be administered in the opposite deltoid to that used for the prime vaccination. For subjects in Stage 1, the third vaccination can be administered in either deltoid. Study vaccines in Stage 2 will be administered in a way that maintains double-blinding. It should be recorded in the CRF in which arm the vaccination has been administered.

If needed, the vaccination might also be administered in the thigh (especially for infants).

No local or topical anesthetic will be used prior to the injection.

The Site Investigational Product Procedures Manual specifies the maximum time that will be allowed between preparation and administration of the study vaccine.

The date and time of each study vaccine administration will be recorded.

7. TREATMENT COMPLIANCE

All study vaccine will be administered at study clinics or satellite vaccination posts. The date and time of each study vaccine administration will be recorded.

In order to support vaccination compliance, a separate Innovative Medicines Initiative (IMI) Ebola+ initiative, EBODAC, with focus on “Deployment and Compliance” was established. The overall objective of EBODAC is to develop a communication and engagement strategy including the development of appropriate technology and tools in order to maximize Ebola vaccination impact.

EBODAC will provide:

1. A communication strategy and tools, including a mobile technology platform, to increase acceptance and compliance with Ebola vaccination and building confidence with the broader population.
2. Tools, services, and mobile technologies to allow an effective recall of the vaccinee for the boost vaccination.
3. An identification tool of vaccinees to ensure correct application of the prime-boost regimen.

8. PRESTUDY AND CONCOMITANT THERAPY

A subject should not receive a live-attenuated vaccine from 30 days before the prime vaccination until 30 days after the boost vaccination unless a vaccine preventable disease such as measles emerges. Immunizations with inactivated vaccines should be administered at least 15 days before

or after administration of any study vaccine in order to avoid any potential interference in efficacy of the routine immunizations or the interpretation of immune responses to study vaccines, as well as to avoid potential confusion with regard to attribution of adverse reactions. Otherwise, a subject will not postpone, forego or delay the receipt of any recommended vaccine according to local schedules (eg, EPI schedule according to the WHO regional office for West Africa). Prior to vaccination of young children in Stage 2, parents will be asked to show the child's EPI vaccination card to check that the child has not received a live-attenuated vaccine within the last 30 days. In case the parent does not have a card or cannot show it, the parent will be asked whether the child has received a measles vaccination within the last 30 days.

Chronic or recurrent use of medications that modify the host immune response (eg, cancer chemotherapeutic agents, systemic corticosteroids) are discouraged.

Analgesic/antipyretic medications (paracetamol) and NSAIDs may be used post-vaccination only in case of medical need (eg, body temperature ≥38.0°C or pain). The use of these medications in Stage 1 and Stage 2 must be documented. Use of these medications as routine prophylaxis prior to study vaccine administration is discouraged.

Subjects who develop anemia during the study (defined as hemoglobin <8 g/dL for children aged ≥1 to <5 years, <9 g/dL for children aged ≥5 to <15 years, and <10 g/dL for subjects aged ≥15 years) will be provided with appropriate supplements (iron, Vitamin C) and followed-up accordingly.

Prestudy therapies administered up to 30 days prior to the start of screening must be recorded at screening. Concomitant therapies must be recorded from the screening onwards until 28 days after the prime vaccination and then again until 28 days post-boost vaccination (Stage 1 and 2) and until 28 days post-third vaccination (Stage 1). At the other time points, concomitant therapies are only to be recorded if given in conjunction with serious adverse events and IREs.

9. STUDY EVALUATIONS

9.1. Study Procedures

9.1.1. Overview

The Time and Events Schedules summarizes the frequency and timing of immunogenicity and safety measurements applicable to this study. Additional study visits may be required if in the investigator's opinion, further clinical or laboratory evaluation is needed.

Additional urine pregnancy tests may be performed, as determined necessary by the investigator or required by local regulation, to establish the absence of pregnancy at any time during the subject's participation in the study.

Approximately 124.2 or 164.4 mL (Stage 1, without or with third vaccination, respectively) or 87.3 or 98.5 mL (Stage 2, long-term follow-up period until 1 year or 2 years post-prime, respectively) of blood will be drawn from adults (≥18 years of age) and approximately 65.6 mL of

blood will be drawn from adolescents (≥13 and <18 years of age) during the study. This volume remains well below the limits of standard blood donation.

For children (<13 years), the study-related blood volumes obtained (including any losses during phlebotomy) will not exceed 3% of the total blood volume during a period of 4 weeks and will not exceed 1% at any single time. The total volume of blood is estimated at 80 to 90 mL/kg body weight; 3% is 2.4 mL blood per kg body weight.[5] The allowable blood volume calculations are based on the 10th percentile for growth charts for 1- to 2-year-old children[13] and 3- to 11-year-old children.[2]

For details on the approximate blood sampling volumes collected by visit and the cumulative blood volumes, refer to the Time and Events Schedules.

Repeat or unscheduled samples may be taken for safety reasons or for technical issues with the samples which could increase the total blood volume indicated above. In case a subject has fever, but does not meet the EVD definition, an unscheduled blood sample may be taken to perform a PCR test to exclude EVD.

Visit windows are provided in the Time and Events Schedules for the different stages in the study. If a subject did not receive a study vaccine on the planned day of vaccination, the timings of the next visits post-vaccination (see Time and Events Schedules) will be determined relative to the actual day of vaccination.

9.1.2. Screening Phase

Up to 28 days before the baseline visit (Day 1; day of prime vaccination), screening assessments as indicated in the Time and Events Schedules will occur. Screening may be split into multiple days or visits. Subjects whose screening period was longer than the protocol-defined 28 days as a result of a study pause or factors outside the control of the study will be allowed to rescreen once.

For male subjects and female subjects of non-childbearing potential, there will be no minimum duration of the screening period and it will last only for the time required to verify eligibility criteria.

For female subjects of childbearing potential, it should be confirmed that adequate birth control measures consistent with what is available and the local standards regarding the use of birth control for subjects participating in clinical studies were used from at least 14 days before the prime vaccination with a negative urine β-hCG pregnancy test at screening, a negative urine β-hCG pregnancy test immediately prior to the prime vaccination (which shall occur no earlier than 14 days after the screening visit) and a negative urine β-hCG pregnancy test immediately prior to the boost vaccination and third vaccination (Stage 1).

All male and female subjects, except for female subjects of non-childbearing potential, will be asked to use adequate birth control for sexual intercourse until at least 3 months after the prime vaccination or up to 28 days after the boost vaccination (whichever takes longer). See Section 4.3 for the requirements of the third vaccination in Stage 1.

Only subjects complying with the criteria specified in Section 4 will be included in the study. The investigator will provide detailed information on the study to the subject and will obtain informed consent/assent (see Section 16.2.3 for details).

After reading but before signing the ICF, subjects (for children, this would be the parent or guardian) must pass the TOU. Subjects in Stage 2 who fail may repeat the test twice. If both the parent or guardian and the child will be enrolled in the study, the parent/guardian will need to complete the TOU twice (ie, one for the parent/guardian and one for the child). In case the parent/guardian consents for multiple children of the same family, the TOU needs to be completed only once, ie, for the first child of the family enrolled in the study. In case the TOU information would change while recruiting multiple children of the same family, the TOU should be completed again for the first child of the family that was enrolled after the TOU change had occurred. The TOU is a questionnaire to document the subject's understanding of the study (for details, see Section 16.1 and Attachment 2).

The following is performed to determine the eligibility requirements:

- Review of all inclusion and exclusion criteria
- Review of prohibitions and restrictions
- Review of medical history and demographics
- Review of prestudy therapies
- Urine pregnancy test (for sexually active women of childbearing potential)
- Blood sampling for hematology and chemistry (fasting or non-fasting)
- Full physical examination (including body length/height and weight)
- Measurement of vital signs (heart rate and blood pressure [supine], body temperature)

The overall eligibility of the subject to participate in the study will be assessed once all screening values and results of any other required evaluations are available. Retesting of values (eg, safety laboratory) that lead to exclusion is allowed once using an unscheduled visit during the screening period to assess eligibility. Study subjects who qualify for inclusion will be contacted and scheduled for enrollment and prime vaccination within 28 days.

All adverse events will be collected from signing of the ICF onwards until 56 days after the boost vaccination in Stage 1 and then again from the day of the third vaccination until 28 days after the third vaccination, and from signing of the ICF onwards until 28 days after the prime vaccination in Stage 2, and then again until 28 days after the boost vaccination. Serious adverse events and IREs will be collected from signing of the ICF onwards until subjects have completed the study.

9.1.3. Vaccination Period

The investigator should ensure that all enrollment criteria have been met during screening. If a subject's clinical status (including available laboratory results or the receipt of additional medical records) after screening but before the prime vaccination on Day 1 changes such that the subject no longer meets all enrollment criteria, then the subject will be excluded from participation in the study.

Following a re-check of the inclusion and exclusion criteria (only for the prime vaccination), check of the contraindications (only for the boost [Stage 1 and Stage 2] and third vaccination [Stage 1]), a urine pregnancy test (for sexually active women of childbearing potential), a brief physical examination (including body length/height and weight in children aged 12 months up to and including 24 months), measurements of vital signs, and collection of a blood sample for hematology and chemistry (for details, see Section 9.3.2), subjects will receive the study vaccine on Day 1, Day 57 and at least 2 years post prime (Stage 1 only) as described in Section 6. Subjects will have a blood draw prior to each vaccination for immunologic assays.

After the prime, boost and third (Stage 1 only) vaccinations, subjects will be asked to remain at the clinic or vaccination post and will be closely observed by the study staff for 60 minutes (Stage 1 prime and boost vaccination) or 30 minutes (Stage 1 third vaccination and Stage 2 prime and boost vaccination) after vaccination, and symptoms of unsolicited and solicited local and systemic adverse events will be evaluated during this period, and documented at the end of the observation period. Vital signs including body temperature will also be obtained at the end of the observation period. The observation time may be extended if deemed necessary by the study staff.

Upon discharge from the clinic or vaccination post after the prime and boost vaccination and after the third vaccination (Stage 1 only), subjects will also receive a diary, a thermometer, and a ruler to measure local solicited reactions and body temperature and will be trained on how to collect this information. Subjects will record symptoms of solicited local and systemic adverse events in the diary in the evening after each vaccination and then daily for the next 7 days. Diaries will be completed at home by either a project field worker who will contact the subject at home or by the subject (for children: the parent/legal guardian) and checked by a project field worker.

Subjects will visit the clinic or vaccination post for assessments 7 days post prime and 7 days post boost as described in the Time and Events Schedules. The investigator or clinical designee will review information from the subject's diary and examine the injection site for occurrences of redness of skin, swelling/induration, pain/tenderness, and/or itching in order to complete the relevant parts of the CRF. The diary should be completed by the end of the Day 8 and Day 64 visit (Stage 1) and the Day 8 and Day 8 post-boost visit (Stage 2). A brief physical examination (including body length/height and weight in children aged 12 months up to and including 24 months at the time of the prime and the boost vaccination) and measurements of vital signs including body temperature will be performed, and a blood sample for hematology and chemistry will be collected.

Subjects in Stage 2 should also visit the clinic on Day 29 for safety assessments (adults and children) and sampling for immunogenicity assessments (adults only).

Subjects in Stage 1 receiving the third vaccination will visit the clinic or vaccination post 4 days post-third vaccination for sampling for immunogenicity assessments. A next visit is scheduled 7 days post-third vaccination. At this time point, they will follow the same assessments and procedures as described above for the 7-day post-prime and 7-day post-boost vaccination visits, except for measurements of vital signs (will not be performed). The diary should be completed by the end of the Day 8 post-third vaccination visit.

Adverse events and serious adverse events will be collected and documented on the CRFs, together with the information on any concomitant medications. For reporting of immediate reportable events (IREs), refer to Section 12.3.3.

9.1.4. Post-Boost and Post-Third Vaccination Period

Stage 1

Subjects will come to the clinic or vaccination post on Day 78 and Day 156 after the prime vaccination for safety evaluations. A brief physical examination and measurements of vital signs including body temperature will be performed, and a blood sample for hematology and chemistry will be collected (Day 78 visit only).

Subjects will have a blood draw for immunologic assays on Day 78 and Day 156 after the prime vaccination.

Subjects in Stage 1 receiving the third vaccination will come to the clinic or vaccination post on the Day 22 post-third vaccination visit for safety evaluations. A brief physical examination will be performed. In addition, a blood draw for immunologic assays will be performed.

Stage 2

Subjects will come to the clinic or vaccination post on the Day 22 post-boost visit (adults and children) and the 3-month post-boost visit (only for young children aged less than 2 years at randomization) for safety evaluations. A brief physical examination and measurements of vital signs including body temperature will be performed.

Subjects will also have a blood draw for immunologic assays on Day 22 post boost.

Children aged less than 2 years at the time of randomization will receive a third vaccination at 3 months post boost, with MenACWY (for children in the control arm) or placebo (Ad26.ZEBOV/MVA-BN-Filo arm). Subject diaries will not be distributed. Children that experience any symptoms will be invited to come for an unscheduled visit as needed.

9.1.5. Long-Term Follow-Up

In both Stage 1 and Stage 2, subjects will be contacted by telephone on a periodic basis between visits during long-term follow-up to assess safety. In addition, subjects may be contacted approximately 1 month after their last visit to inquire after their general health status. These calls will not be included in the clinical database.

Stage 1

Subjects will enter the long-term follow-up, and visit the clinic or vaccination post on Days 180, 240 and 360 post prime for collection of serious adverse event information and IREs.

Blood will be drawn for immunologic assays at the Day 360 post-prime visit.

Thereafter, subjects in Stage 1 who do not consent to receive the third vaccination will visit the clinic or vaccination post every 6 months until 36 months post prime for collection of serious adverse event information and IREs and immunogenicity assessments.

Subjects who consent to receive the third vaccination, will visit the clinic at the Day 540 post-prime visit and will enter the third vaccination schedule 6 months later (at least 2 years post prime). During the long-term follow-up period of the third vaccination schedule, subjects will visit the clinic or vaccination post at the 6-month post-third vaccination visit and on the Day 360 post-third vaccination visit. At these visits, serious adverse event information and IREs will be collected and blood will be drawn for immunologic assays. A brief physical examination will only be performed when related to serious adverse events and IREs.

Stage 2

Subjects will enter the long-term follow-up, and visit the clinic or vaccination post on the 6-month post-boost visit and the Day 360 post-prime visit. Subjects in Stage 2 will be approached for enrollment into the VAC52150EBL3005 study for further follow-up of safety and immunogenicity for up to a total of 60 months after the prime vaccination. Adult subjects who consent to extend the long-term follow-up period for up to 2 years post prime, will also visit the clinic or vaccination post on the Day 540 post-prime visit and the 2-year post-prime visit. At all visits, serious adverse event information and IREs will be collected. Vital signs will be measured on the 6-month post-boost visit only. Brief physical examinations at the long-term follow-up visits will only be performed when related to serious adverse events and IREs.

Blood will be drawn for immunologic assays at the 6-month post-boost visit and the Day 360 post-prime visit (all subjects) and at the 2-year post-prime visit (adults only, if applicable).

9.1.6. Evaluation of Fever

Subjects may exhibit general signs and symptoms associated with the administration of the study vaccines, including fever and chills (see Section 1.2.4). Subjects will be counseled to immediately call or contact the study personnel if they are feeling ill following vaccination and study personnel will investigate any fever ≥38.0°C following the fever management algorithm (see Attachment 3) and national guidelines.

If the subject experiencing fever ≥38.0°C does not meet the modified case definition for EVD (available in Attachment 3), study personnel will refer the subject to the hospital if the subject needs medical care and, if the subject has mild symptoms and does not need immediate medical care, or the fever is <38.0°C and study personnel considers that it merits attention, investigate any cause of fever (malaria, respiratory infections, etc.) and treat it appropriately. The fever will be

classified as “vaccine-related” if other causes of fever are ruled out. An unscheduled blood sample may be taken to perform a PCR test to exclude EVD.

If fever is not deemed to be a reaction to the vaccine and another cause needing medical care is detected, the subject will be referred for further investigation/confirmatory diagnosis and eventual treatment at the corresponding hospital.

In case a subject has fever, and meets the modified EVD case definition, study personnel will call the Ebola alert line (if still in place) and coordinate with the corresponding authorities the transport and evacuation of the patient to the Ebola holding or treatment center for evaluation, diagnosis and management. Note that Sierra Leone was declared Ebola-free on 7 November 2015. A 90-day period of enhanced surveillance will end 5 February 2016. The evaluation of fever pertaining to Ebola disease will be carried out until instructions to alter management are received from the relevant authorities in the country.

In order to reduce the incidence of fever induced by malaria, subjects in Stage 2 will receive insecticide treated bed nets (ITNs) at the time of their prime vaccination. Subjects currently enrolled in Stage 1 will receive these at their next convenient booked visit. To further minimize the event of a vaccine-associated fever being misclassified as an EVD suspect case, the study will keep close contact/collaboration with the national Ebola surveillance center to monitor any possible EVD that has been detected in the past 21 days or under investigation in the community so that the fever investigation can have up-to-date information to rule out an epidemiological link.

9.1.7. Early Withdrawal

Subjects withdrawn from study vaccine administration must not receive any further study vaccine, but should continue to be monitored for safety. Subjects may be followed-up for immunogenicity but only if this does not result in an additional risk (see Section 10.2).

In case of early withdrawal due to an adverse event, the investigator or clinical designee will collect all information relevant to the adverse event and safety of the subject, and will follow the subject until resolution of the adverse event or until reaching a clinically stable endpoint. If feasible, blood will be drawn for immunologic assays.

Subjects who wish to withdraw consent will be offered an optional visit for safety follow-up (before the formal withdrawal of consent). The subject has the right to refuse. For more information, see Section 10.4.

9.2. Immunogenicity

9.2.1. Endpoints

The immunogenicity endpoint of the study includes the evaluation of antibody responses. Depending on assay and/or sample availability any of the following assays might be performed:

- EBOV GP ELISA: to determine the binding antibody levels elicited by vaccination.

- VNA: neutralizing antibody reactivity against the EBOV GP defined as the serum titer that is able to inhibit viral infection by a certain percentage (IC_{50}, IC_{80} or IC_{90}).
- ELISA, VNA, and/or PRNT: to explore the binding and/or neutralizing antibody responses against the adenovirus and/or MVA vector.

9.2.2. Evaluations

Venous blood samples will be collected from all subjects from Stage 1 and Stage 2 to assess humoral immune responses to the vaccines.

The samples will be collected at the time points and in volumes as indicated in the Time and Events Schedules.

The immunologic assays and purposes are summarized in Table 3 below. Sample collection and processing will be performed by the site staff according to current versions of approved standard operating procedures. The Laboratory Manual contains further details regarding the collection, handling, labeling, and shipment of blood samples to the respective laboratories.

The analysis of the immunogenicity samples will be done at a central laboratory.

Table 3: Summary of Potential Immunologic Assays (Serology)

Assay	Purpose
ELISA	Analysis of antibodies binding to EBOV GP
Virus neutralization assay	Analysis of neutralizing antibodies to EBOV GP
ELISA and/or neutralization assay	Analysis of binding and/or neutralizing antibodies against Ad26 and/or MVA vectors

EBOV: Ebola virus; ELISA: enzyme-linked immunosorbent assay; GP: glycoprotein

9.3. Safety

9.3.1. Endpoints

The safety and tolerability endpoints are:

- Solicited local and systemic adverse events (reactogenicity), collected until 7 days after the prime and boost vaccination (Stage 1 and Stage 2) and the third vaccination (Stage 1) as recorded by the subjects (or, when appropriate, by a project field worker, a caregiver, surrogate, or the subject's legally acceptable representative) in a diary during daily home contacts with a project field worker.
- Solicited local and systemic adverse events (reactogenicity), collected until 60 minutes after the prime and boost vaccination and until 30 minutes after the third vaccination in all subjects from Stage 1, and until 30 minutes after each vaccine administration in all subjects from Stage 2.
- Unsolicited adverse events, collected by the investigator or the clinical designee from provision of informed consent onwards until 56 days after the boost vaccination in Stage 1 and then again from the day of the third vaccination until 28 days after the third vaccination, and from provision of informed consent onwards until 28 days after the prime vaccination in Stage 2 and then again until 28 days after the boost vaccination. (Note: events that started

before the third vaccination but are still present at the time of third vaccination should also be recorded).

- Serious adverse events, including deaths, and IREs collected in all subjects from Stage 1 and Stage 2 from provision of informed consent onwards until the subject's last study visit.

9.3.2. Evaluations

Details regarding the IDMC are provided in the IDMC Charter. See also Section 11.7.

Serious adverse events, including deaths, and IREs will be collected from all subjects and during all stages of the study from signing of the ICF onwards until the subject's last study visit.

Deaths in Stage 1 and Stage 2 will be followed-up and investigated actively to determine if the death is related to the vaccine. If, according to the WHO case definition and algorithm, the death is suspected to be due to EVD, the case will be referred to the corresponding local Ebola response authorities for investigation per WHO protocol/guidance.[a]

Any death will be immediately followed-up, recorded, reported per standard operating procedures, investigated and referred to the IDMC. If the death is deemed to be vaccine-related, the study will pause until IDMC has reviewed all pertinent data and allocates causality. If, according to the WHO case definition and algorithm, the death is suspected to be due to EVD, the case will be referred to the corresponding local authorities for investigation (verbal autopsy) per WHO protocol/guidance.

The study will further include the following evaluations of safety and tolerability according to the time points in the Time and Events Schedules:[b]

Adverse Events

All adverse events will be reported by the subject (or, when appropriate, by a caregiver, surrogate, or the subject's legally acceptable representative) from signing of the ICF onwards until 56 days post boost in Stage 1 and then again from the day of the third vaccination until 28 days after the third vaccination, and from signing of the ICF onwards until 28 days after the prime vaccination in Stage 2 and then again until 28 days after the boost vaccination.

Solicited local and systemic adverse events (reactogenicity; see below) will be reported by the subject (or, when appropriate, by a project field worker, a caregiver, surrogate, or the subject's legally acceptable representative) in the diary each day until 7 days after the prime and boost vaccination (Stage 1 and Stage 2) and the third vaccination (Stage 1). If a solicited local or systemic adverse event is not resolved at Day 8, Day 64 or Day 8 post-third vaccination (Stage 1), or at Day

---

[a] Available at: http://www.who.int/csr/resources/publications/ebola/Corrected%20CoverEboladoc1.pdf?ua=1, accessed 12 August 2016.

[b] Note that diaries are only applicable for the prime and boost vaccination in Stage 1 and Stage 2, and for the third vaccination in Stage 1. Solicited local and systemic adverse events (reactogenicity) will be reported until 60 minutes after the prime and boost vaccination in all subjects from Stage 1 and until 30 minutes after the third vaccination in all subjects from Stage 1 and after the prime and boost vaccination in all subjects from Stage 2.

8 or Day 8 post boost (Stage 2), the follow-up information will be captured on the diary and the date of last symptoms and maximum severity will be recorded in the diary after resolution.

Any clinically relevant changes occurring during the study must be recorded on the Adverse Event section of the CRF.

Any clinically significant abnormalities persisting at the end of the study/early withdrawal will be followed by the investigator or clinical designee until resolution or until a clinically stable endpoint is reached.

Adverse events will be followed by the investigator or clinical designee as specified in Section 12.

Solicited Adverse Events

Solicited adverse events are precisely defined events that subjects are specifically asked about and which are noted in the diary. The investigator or clinical designee should discuss the information from the diary with the subject or the subject's legally acceptable representative, document the relevant information in the clinic chart, and complete the relevant parts of the CRF as described in the CRF Completion Guidelines.

Solicited Local (Injection Site) Adverse Events

Instructions will be provided on how to note the following occurrences at the injection site daily until 7 days after the prime and boost vaccination (Stage 1 and Stage 2) and the third vaccination (Stage 1).

- redness of skin
- pain/tenderness
- swelling/induration
- itching at the injection site

These occurrences will be recorded in the diary.

Solicited Systemic Adverse Events

Instructions will be provided on how to record daily body temperature using a thermometer provided for home use. Body temperature should be recorded in the evening after the prime and boost vaccination (Stage 1 and Stage 2) and the third vaccination (Stage 1), and then daily for the next 7 days in the diary. Body temperature should be measured at approximately the same time each day. If more than one measurement is made on any given day, the highest value will be recorded in the CRF. Any fever will be investigated by the investigator or clinical designee for the possibility of Ebola. A body temperature equal to or higher than 38.0°C will be recorded by the investigator or clinical designee as fever. For details, refer to Section 9.1.6.

Instructions will also be provided on how to note the following symptoms/signs in the diary until 7 days after the prime and boost vaccination (Stage 1 and Stage 2) and the third vaccination (Stage 1):

- for preverbal children/infants:
  - body temperature
  - reduced activity, somnolence, fatigue
  - loss of appetite
  - vomiting
  - irritability/fussiness/crying/screaming

- for young children, adolescents, and adults:
  - body temperature
  - fatigue/malaise
  - chills
  - headache
  - nausea/vomiting
  - muscle pain
  - joint pain

Clinical Laboratory Tests

Samples will be collected for hematology and serum chemistry. The investigator must review the laboratory report, document this review, and record any clinically relevant changes on the adverse event page of the CRF. Laboratory reports must be filed with the source documents.

The following tests will be performed by the local laboratory at the time points indicated in the Time and Events Schedules unless otherwise specified.

- Hematology Panel
  - hemoglobin
  - hematocrit
  - RBC count
  - platelet count
  - WBC count with differential

- Serum Chemistry Panel
  - AST
  - ALT
  - creatinine

Additional clinical laboratory assessments to be performed are as follows:

- Urine pregnancy test for sexually active women of childbearing potential before each study vaccination (Stage 1 and Stage 2)

Laboratory values will be determined according to the Toxicity Tables for Use in Trials Enrolling Healthy Adults (Attachment 4) or the Toxicity Tables for Use in Trials Enrolling Children Greater Than 3 Months of Age (Attachment 5).

Any severe (grade 3) non-serious abnormal laboratory values occurring from signing of the ICF onwards until 56 days after the boost vaccination in Stage 1 and then again from the day of the third vaccination until 28 days after the third vaccination, and from signing of the ICF onwards

until 28 days after the prime vaccination in Stage 2 and then again until 28 days after the boost vaccination must be recorded on the adverse event page of the CRF. Thereafter, recording is limited to serious adverse events and IREs. All events should be followed to resolution, or until reaching a clinically stable endpoint.

Vital Signs

Vital sign measurements (body temperature, heart rate, systolic and diastolic blood pressure) will be performed at the time points indicated in the Time and Events Schedules. Blood pressure and heart rate measurements should be preceded by at least 5 minutes of rest in supine position. Confirmatory measurements can be performed if inconsistent with a prior measurement. Heart rate will be measured manually. Manual blood pressure measurement techniques will be used only if an automated device is not available.

Any clinically relevant abnormalities occurring from signing of the ICF onwards until 56 days after the boost vaccination in Stage 1 and then again from the day of the third vaccination until 28 days after the third vaccination, and from signing of the ICF onwards until 28 days after the prime vaccination in Stage 2 and then again until 28 days after the boost vaccination must be recorded on the adverse event page of the CRF. Thereafter, recording will be limited to serious adverse events, including deaths and IREs. All events should be followed to resolution, or until reaching a clinically stable endpoint.

Physical Examination

A full physical examination (including body length/height and weight) will be carried out at screening. At other visits, a brief, symptom-directed examination (including body length/height and weight in children aged 12 months up to and including 24 months at the time of the prime and the boost vaccination) will be performed based on any clinically relevant issues, clinically relevant symptoms and medical history. The symptom-directed physical examination may be repeated if deemed necessary by the investigator. Physical examinations will be performed by the investigator or by designated medically-trained clinician.

Any clinically relevant abnormalities occurring from signing of the ICF onwards until 56 days after the boost vaccination in Stage 1 and then again from the day of the third vaccination until 28 days after the third vaccination, and from signing of the ICF onwards until 28 days after the prime vaccination in Stage 2 and then again until 28 days after the boost vaccination must be recorded on the adverse event page of the CRF. Thereafter, recording will be limited to serious adverse events, including deaths, and IREs. All events should be followed to resolution, or until reaching a clinically stable endpoint.

9.4. Sample Collection and Handling

The actual dates of sample collection must be recorded in the CRF or laboratory requisition form. See the Time and Events Schedules for the timing and frequency of all sample collections.

Instructions for the collection, handling, storage, and shipment of safety samples will be provided by the local laboratories.

Instructions for the collection, handling, storage, and shipment of immunogenicity samples will be provided in the Laboratory Manual. Collection, handling, storage, and shipment of samples must be under the specified, and where applicable, controlled temperature conditions as indicated in the Laboratory Manual.

10. SUBJECT COMPLETION/WITHDRAWAL

10.1. Completion

A subject will be considered to have completed the study if he or she:

- Has completed all study assessments on the Day 360 visit (children and adolescents) in Stage 2, the Day 360 visit or the 2-year post-prime visit (if applicable) (adults) in Stage 2 or on the 36-month post-prime visit or the 1-year post-third vaccination visit (if applicable) in Stage 1.

AND (if applicable)

- Has been contacted 1 month post last visit to inquire after their general health status.

Upon completion of the study, sites may offer MenACWY to the Ad26.ZEBOV/MVA-BN-Filo arm and the Ad26.ZEBOV/MVA-BN-Filo vaccine (if licensed and/or WHO-prequalified) to the control arm upon consultation with the health authorities.

10.2. Discontinuation of Study Vaccine

If a subject's study vaccine must be discontinued before the end of the vaccination schedule, this will not result in automatic withdrawal of the subject from the study.

Subjects will be withdrawn from study vaccine administration for the reasons listed below. These subjects must not receive any further study vaccine, but should continue to be monitored for safety and for immunogenicity if this does not result in a safety risk for the subject.

- Pregnancy
- Any adverse event considered at least possibly related to study vaccine, worsening of clinical status or intercurrent illnesses that, in the opinion of the investigator, requires discontinuation from study vaccine
- Confirmed EVD
- If the randomization code is broken or the treatment allocation has been inadvertently revealed at the study site.
  Note: Unless the label warrants a second vaccination of MenACWY in the control group.

10.3. Contraindications to Vaccination

The following events constitute a contraindication to prime or boost vaccination (Stage 1 and Stage 2) or third vaccination (Stage 1). If any of these events occur at the scheduled time for vaccination, the subject may be vaccinated within the time window specified in the Time and Events Schedules, or be withdrawn from that vaccination at the discretion of the investigator and after consultation with the sponsor:

- Illness prior to vaccination (this does not include acute illnesses such as diarrhea or mild upper respiratory tract infection unless accompanied by body temperature ≥37.5°C in Stage 1 and 38.0°C in Stage 2).
- Fever (body temperature ≥38.0°C) prior to vaccination.

Any fever will be promptly investigated according to national guidelines for the possibility of Ebola. For further details, refer to Section 9.1.6.

Note: *In case the boost vaccination (Stage 1 and Stage 2) or third vaccination (Stage 1) is postponed, the timing of the safety/immunogenicity visits post boost or post-third vaccination will be planned relative to the actual vaccination day.*

A subject will not be given the boost vaccination (Stage 1 and Stage 2) or third vaccination (Stage 1) if he or she experiences any of the following events at any time after the prior vaccinations:

1. Anaphylaxis clearly attributable to vaccination with study vaccine; *OR*
2. Generalized urticaria within 72 hours of vaccination considered to be at least possibly related to study vaccine; *OR*
3. A serious adverse event considered to be at least possibly related to study vaccine; *OR*
4. Injection site ulceration, abscess or necrosis considered to be at least possibly related to the study vaccine; *OR*
5. Any other safety concern threatening the subject's safety or persisting clinically significant abnormality considered to be related to prior vaccinations.

Subjects experiencing any of the events described above must not receive any further study vaccine, but should be monitored for safety and for immunogenicity according to the protocol.

10.4. Withdrawal From the Study

Each subject has the right to withdraw at any time for whatever reason without affecting the right to treatment by the investigator. The investigator should make an attempt to contact subjects who did not return for scheduled visits or follow-up. Although the subject is not obliged to give reason(s) for withdrawing early, the investigator should make a reasonable effort to ascertain the reason(s) while fully respecting the subject's rights.

A subject will be withdrawn from the study for any of the following reasons:

- Repeated failure to comply with protocol requirements
- Decision by the sponsor to stop or cancel the study
- Decision by the investigator to withdraw subjects
- Decision by local regulatory authorities and Institutional Review Board/Independent Ethics Committee (IRB/IEC) to stop or cancel the study
- Lost to follow-up
- Withdrawal of consent
- Death

If a subject in Stage 1 or Stage 2 is lost to follow-up, every reasonable effort must be made by the study site personnel to contact the subject and to determine the reason for discontinuation/withdrawal. The measures taken for at least 3 efforts to follow-up must be documented. No additional subjects will be enrolled in case a subject withdraws after the prime vaccination.

If a subject withdraws prematurely from the study, early withdrawal assessments should be obtained (see Section 9.1.7). When a subject withdraws before completing the study, the reason for withdrawal should be documented in the CRF and in the source document.

A subject who wishes to withdraw consent from participation in the study will be offered an optional visit for safety follow-up (before formal withdrawal of consent). The subject has the right to refuse.

Withdrawal From the Use of Samples in Future Research

Subjects in Stage 1 and 2 may withdraw consent for use of samples for research (refer to Section 16.2.5). In such a case, samples will be destroyed after they are no longer needed for the clinical study. Details of the sample retention for research are presented in the ICF.

11. STATISTICAL METHODS

Statistical analysis will be done by the sponsor or under the authority of the sponsor. A general description of the statistical methods to be used to analyze the safety and immunogenicity data is outlined below. Full details will be provided in the Statistical Analysis Plan (SAP) which will be developed by the sponsor in collaboration with the London School of Hygiene & Tropical Medicine. The SAP will be finalized and signed off and submitted for review to the appropriate regulatory authorities before the first subject will be vaccinated in Stage 1.

The primary analysis of immunogenicity and safety in Stage 1 will be performed when all subjects have completed the study or have discontinued earlier. In Stage 2, primary analyses will be performed for adults (≥18 years) and children (≥1 to 17 years) when these respective groups complete the study or subjects have discontinued earlier. One primary analysis for all children is planned, presenting the data by age group (12 to 17 years, 4 to 11 years, 1 to 3 years, inclusive). Interim analyses may be performed during the study per age group (ie, ≥18 years, 12 to 17 years,

4 to 11 years, 1 to 3 years, inclusive) for the purpose of informing future program-related decisions or for regulatory purposes in a timely manner (see Section 11.6).

The analysis of immunogenicity and safety are further discussed in Sections 11.4 and 11.5, respectively.

11.1. Analysis Sets

The Safety analysis set will include all subjects who received at least one study vaccination. Data will be shown by treatment group (as treated).

The Per Protocol Immunogenicity analysis set includes all randomized (for Stage 2) and vaccinated subjects from any stage who fulfill the following criteria:

- who received the prime and boost vaccination,
- who have data from baseline and at least one post-vaccination immunogenicity blood draw,
- who have no major protocol violations or did not receive a delayed boost vaccination due to a pause that could affect the immune response.

The Per Protocol Immunogenicity analysis set is the primary analysis population for immunogenicity analyses.

11.2. Subject Information

For each analysis set, demographic characteristics (eg, age and sex) and screening/baseline characteristics (eg, physical examination, medical history) will be tabulated and summarized with descriptive statistics for the vaccinated group and the control group (Stage 2 only).

11.3. Sample Size Determination

Safety

Table 4 shows the number of subjects planned for safety and immunogenicity by age group for Stage 1 and Stage 2.

Table 4: **Number of Subjects Planned per Stage and Population for Safety and Immunogenicity Assessments**

**Population Age (years inclusive)**	**Safety and Immunogenicity Data**		
	Stage 1	***Stage 2***	
	Ebola vaccine[a]	Ebola vaccine[b]	Active control[c]
Adults (≥18 years)	**40**	**300**	**100**
12 to 17 years	-	**144**	**48**
4 to 11 years	-	**144**	**48**
1 to 3 years	-	**144**	**48**
**Total**	**40**	**732**	**244**

[a] Prime-boost regimen of Ad26.ZEBOV followed by MVA-BN-Filo (boost vaccination) and Ad26.ZEBOV (third vaccination).
[b] Prime-boost regimen of Ad26.ZEBOV followed by MVA-BN-Filo.

[c] The active control will consist of the WHO-prequalified MenACWY vaccine.

As shown in Table 5 below, the sample sizes selected indicate that the probability of observing at least 1 (serious) adverse event occurring at a rate of 1/10 or more in each group (Stage 1; adults, Stage 2; adults, 12-17 years, 4-11 years, and 1-3 years) is ≥99%. The probability of observing at least 1 (serious) adverse event occurring at a rate of 1/100 is 95% with 300 subjects. The probability of observing at least 1 (serious) adverse event occurring at a rate of 1/1,000 is 26% with 300 subjects and 52% with 732 subjects. If no events are observed during Stage 2 for a specific adverse event, then the Bayesian posterior probability that the event rate is below 1/1,000 equals 41% for 144 subjects (ie, the number of children in each age group), 56% for 300 subjects (ie, the number of adults), and 77% with 732 subjects when using Jeffreys' prior.

Table 5: Probability of Observing at Least One (Serious) Adverse Event

N	Probability of observing at least one (serious) adverse event when incidence equals		
	**1/10**	**1/100**	**1/1,000**
40	99%	33%	4%
144	100%	76%	13%
300	100%	95%	26%
732	100%	100%	52%

Table 6 represents the 95% upper limit for the true proportion for various sample sizes associated with no reported (serious) adverse events. For example when 40 subjects are vaccinated, the observation of 0 reactions would be associated with a 95% confidence that the true rate is less than 7.2%.

Table 6: Sample Size Justification Safety Evaluation in Stage 1 and 2

N	95% upper limit for the true proportion of a (serious) adverse reaction when no events are reported
40	7.2%
144	2.1%
300	1.0%
732	0.4%

Immunogenicity

As part of Amendment 5, the number of children in each age group was doubled to compensate for an expected higher variability in immunogenicity parameters.

11.4. Immunogenicity Analyses

All subjects in the Per Protocol Immunogenicity analysis set as defined in Section 11.1 will be included in the analysis.

Descriptive statistics (actual values, changes from baseline, if applicable) will be calculated for continuous immunologic parameters at all time points analyzed. Geometric mean concentrations and/or titers and changes together with a corresponding 95% confidence interval will be calculated. Graphical representations of changes in immunologic parameters will be prepared, as applicable. Frequency tabulations will be calculated for discrete (qualitative) immunologic parameters at all

time points and response analyses will be performed. Different definitions of immunologic response will be further detailed in the SAP.

In addition, a longitudinal data analysis of the immunogenicity response over time will be explored. Least square means with their 95% confidence intervals will be calculated from this model.

11.5. Safety Analyses

No formal statistical testing of safety data is planned. Safety analysis will be performed on the safety analysis set. Data from Stage 1 will be summarized separately. Safety data from Stage 2 will be shown by treatment group (as treated), and by age group.

Safety data will be analyzed descriptively (including 95% confidence intervals, if applicable) for subjects receiving control or active vaccine.

Baseline for all safety parameters will be defined as the last value before the prime vaccination.

For Stage 1 and 2, the following summaries are planned:

Adverse Events (Including Reactogenicity)

The verbatim terms used in the CRF by investigators to report adverse events will be coded using the Medical Dictionary for Regulatory Activities (MedDRA). All reported adverse events (solicited local, solicited systemic, and unsolicited) during the treatment period (ie, treatment-emergent adverse events, and adverse events that have worsened since baseline) will be included in the analysis. For each adverse event, the number and percentage of subjects who experience at least one occurrence of the given event will be summarized. Summaries, listings, datasets and/or subject narratives may be provided as appropriate, for those subjects who die, discontinue study vaccinations due to an adverse event, or experience a severe or serious adverse event. The analysis of solicited and unsolicited adverse events will be done based on the safety analysis set.

Clinical Laboratory Tests

Laboratory abnormalities will be determined according to the toxicity grading tables in Attachment 4 and Attachment 5, and in accordance with the normal ranges of the clinical laboratory.

The worst laboratory abnormalities following vaccination will be tabulated.

Vital Signs

Vital signs abnormalities following vaccination will be tabulated by worst abnormality grade.

Physical Examination

Physical examination abnormalities following vaccination will be tabulated by worst abnormality grade. BMI will be calculated using the recording of height at screening. Body weight and BMI results will be tabulated descriptively.

For children, weight and height for adequacy of growth milestones percentiles will be calculated using the recording of those measurements at screening (baseline) compared to study progression/visits. Percentile results (according to local growth charts) will be tabulated descriptively.

11.6. Interim Analysis

Interim analyses may be performed during the study for the purpose of informing future program-related decisions and for regulatory purposes in a timely manner.

11.7. Independent Data Monitoring Committee (IDMC)

An IDMC will be established to monitor safety data on an ongoing basis to ensure the continuing safety of the subjects enrolled in this study.

The IDMC will consist of at least one medical expert in the relevant therapeutic area and at least one statistician.

The IDMC responsibilities, authorities, frequency and timing of the evaluations and procedures will be documented in its charter. All analyses that are planned to support the IDMC evaluations will be included in the SAP. Additional reviews can be requested by the IDMC.

A schematic presentation of the IDMC schedule is provided in Attachment 1.

11.7.1. Safety

The IDMC will review all available 7-day post-prime safety data (including clinical laboratory results) before the first subject of the current cohort will receive the boost:

- In Stage 1 to determine whether:
  - the boost vaccination in Stage 1 can be initiated, and
  - the prime vaccination in Stage 2 (subjects aged 12 to 17 years inclusive) can be initiated.
- In Stage 2 (subjects aged 12 to 17 years inclusive) to determine whether:
  - the boost vaccination in subjects aged 12 to 17 years inclusive can be initiated, and
  - the prime vaccination of subjects aged 4 to 11 years inclusive can be initiated.
- In Stage 2 (subjects aged 4 to 11 years inclusive) to determine whether:
  - the boost vaccination in subjects aged 4 to 11 years inclusive can be initiated, and
  - the prime vaccination of subjects aged 1 to 3 years inclusive can be initiated.
- In Stage 2 (subjects aged 1 to 3 years inclusive) to determine whether:
  - the boost vaccination in subjects aged 1 to 3 years inclusive can be initiated.

The IDMC will also review all cumulative safety data including the 7-day post-boost safety data (including clinical laboratory results) to determine if the boost vaccination in the next stage and/or next age group can be given. An IDMC meeting will therefore take place with all available data

(also including the 7-day post-prime safety data within that age group) before boost vaccination will be administered.

A schematic presentation of the IDMC schedule is provided in Attachment 1.

11.8. Clinical Steering Committee and Management Committee

Oversight of the study will be conducted by the Trial Management Team which will consist of members from Janssen (the sponsor), as well as key collaborators from the participating countries. This Trial Management Team will be responsible for protocol development and ensuring proper study execution, subject to the approval of the Clinical Steering Committee.

The Clinical Steering Committee will have the final say on all matters relating to the study, to the extent that a decision could not be taken at a lower level.

11.9. Pausing Rules

The investigators and the sponsor’s medical monitor will review the safety of enrolled subjects on an ongoing basis. The sponsor’s medical monitor will be involved in all discussions and decisions (see Section 12.4).

If any of the events described in Section 11.9.1 occur in any subject who received at least one dose of study vaccine in the study (at any site), that site investigator will halt the vaccination of further subjects and the sponsor’s medical monitor will be notified immediately. The sponsor’s medical monitor will then also inform all the other investigators to halt further vaccination as well.

For the events described below, the sponsor’s medical monitor notifies the IDMC immediately. Within 3 business days, the IDMC will convene to review the available safety data as outlined in the charter and to discuss study suspension or discontinuation of further vaccination or to decide that vaccination may resume. The sites will be allowed to resume activities upon receipt of a written notification from the sponsor. The pausing rules will be re-set each time and the same criteria have to be met to halt further vaccination again. The communications from the IDMC will be forwarded by the investigator to the IEC/IRB according to local standards and regulations and by the sponsor to the relevant health authorities.

11.9.1. Stage 1

1. Death in any subject considered to be at least possibly related to the study vaccine; *OR*
2. An anaphylactic reaction within 24 hours of vaccination or the presence of generalized urticaria within 72 hours of vaccination in any subject considered to be at least possibly related to the study vaccine; *OR*
3. A life-threatening or other serious adverse event in any subject considered to be at least possibly related to the study vaccine.

If any of the following events occur in subjects who received at least one dose of study vaccine in the study (across all sites), the sponsor's medical monitor will notify all investigators to halt vaccination of further subjects.

4. Three or more subjects experience a severe (grade 3) (non-serious) unsolicited adverse event (of the same type) considered to be related to any of the study vaccines that persists for 3 or more days; OR
5. Three or more subjects experience a persistent (upon repeat testing) severe (grade 3) (non-serious) abnormality (including unexplained hematuria) related to the same laboratory parameter and considered to be related to any of the study vaccines; OR
6. Three or more subjects experience the same severe (grade 3) (non-serious) solicited systemic reaction considered to be related to any of the study vaccines that persists for 3 or more days (subjective systemic reaction corroborated by study personnel).

11.9.2. Stage 2

11.9.2.1. Adults and Adolescents (Aged ≥12 Years)

1. Death in any subject considered to be at least possibly related to the study vaccine; *OR*
2. An anaphylactic reaction within 24 hours of vaccination or the presence of generalized urticaria within 72 hours of vaccination in any subject considered to be at least possibly related to the study vaccine; *OR*
3. A life-threatening or other serious adverse event in any subject considered to be at least possibly related to the study vaccine.

11.9.2.2. Children (Aged ≥1 to 11 Years, Inclusive)

1. Death in any subject considered to be at least possibly related to the study vaccine; *OR*
2. An anaphylactic reaction within 24 hours of vaccination or the presence of generalized urticaria within 72 hours of vaccination in any subject considered to be at least possibly related to the study vaccine; *OR*
3. A life-threatening or other serious adverse event in any subject considered to be at least possibly related to the study vaccine.

If any of the following events occur in subjects **within the same age group (≥1-3 years OR ≥4-11 years)** who received at least one dose of study vaccine in the study (across all sites), the sponsor's medical monitor will notify all investigators to halt vaccination of further subjects.

4. Three or more subjects experience a severe (grade 3) (non-serious) unsolicited adverse event (of the same type) considered to be related to any of the study vaccines that persists for 3 or more days.
5. Three or more subjects experience a persistent (upon repeat testing) severe (grade 3) (non-serious) abnormality (including unexplained hematuria) related to the same laboratory parameter and considered to be related to any of the study vaccines; OR

6. Three or more subjects experience the same severe (grade 3) (non-serious) solicited systemic reaction considered to be related to any of the study vaccines that persists for 3 or more days (subjective systemic reaction corroborated by study personnel.)

12. ADVERSE EVENT REPORTING

Timely, accurate, and complete reporting and analysis of safety information from clinical studies are crucial for the protection of subjects, investigators, and the sponsor, and are mandated by regulatory agencies worldwide. The sponsor has established standard operating procedures in conformity with regulatory requirements worldwide to ensure appropriate reporting of safety information; all clinical studies conducted by the sponsor or its affiliates will be conducted in accordance with those procedures.

12.1. Definitions

12.1.1. Adverse Event Definitions and Classifications

Adverse Event

An adverse event is any untoward medical occurrence in a clinical study subject administered a medicinal (investigational or non-investigational) product. An adverse event does not necessarily have a causal relationship with the treatment. An adverse event can therefore be any unfavorable and unintended sign (including an abnormal finding), symptom, or disease temporally associated with the use of a medicinal (investigational or non-investigational) product, whether or not related to that medicinal (investigational or non-investigational) product. (Definition per International Council for Harmonisation [ICH].)

This includes any occurrence that is new in onset or aggravated in severity or frequency from the baseline condition, or abnormal results of diagnostic procedures, including laboratory test abnormalities.

Note: The sponsor collects adverse events starting with the signing of the ICF (refer to Section 12.3.1 for time of last adverse event recording).

Serious Adverse Event

A serious adverse event based on ICH and EU Guidelines on Pharmacovigilance for Medicinal Products for Human Use is any untoward medical occurrence that at any dose:

- Results in death
- Is life-threatening
  (The subject was at risk of death at the time of the event. It does not refer to an event that hypothetically might have caused death if it were more severe.)
- Requires inpatient hospitalization or prolongation of existing hospitalization
- Results in persistent or significant disability/incapacity
- Is a congenital anomaly/birth defect

- Is a suspected transmission of any infectious agent (except the vaccine virus vectors themselves) via a medicinal product
- Is Medically Important*

*Medical and scientific judgment should be exercised in deciding whether expedited reporting is also appropriate in other situations, such as important medical events that may not be immediately life threatening or result in death or hospitalization but may jeopardize the subject or may require intervention to prevent one of the other outcomes listed in the definition above. These should usually be considered serious.

If a serious and unexpected adverse event occurs for which there is evidence suggesting a causal relationship between the study vaccine and the event (eg, death from anaphylaxis), the event must be reported as a suspected unexpected serious adverse reaction (SUSAR) (even after the study is over, if the sponsor, IDMC or investigator becomes aware of them).

Should any study subject develop EVD during the study period, this event will be captured as a serious adverse event.

Unlisted (Unexpected) Adverse Event/Reference Safety Information

An adverse event is considered unlisted if the nature or severity is not consistent with the applicable product reference safety information. For Ad26.ZEBOV and for MVA-BN-Filo, the expectedness of an adverse event will be determined by whether or not it is listed in the respective Investigator's Brochures and Addenda, if applicable.[8,9] For MenACWY, refer to the applicable vaccine prescribing information.

Adverse Event Associated With the Use of the Vaccine

An adverse event is considered associated with the use of the study vaccine if the attribution is possible, probable, or very likely by the definitions listed in Section 12.1.2.

An adverse event is considered not associated with the use of the study vaccine if the attribution is not related or doubtful by the definitions listed in Section 12.1.2.

Immediate Reportable Events

The following list of neuroinflammatory disorders are categorized as IREs, and should be reported to the sponsor within 24 hours of becoming aware of the event using the IRE Form. Relevant data from the IRE Form will be captured in the clinical database.

- Cranial nerve disorders, including paralyses/paresis (eg, Bell's palsy)
- Optic neuritis
- Multiple sclerosis
- Transverse myelitis
- Guillain-Barré syndrome, including Miller Fisher syndrome, Bickerstaff's encephalitis and other variants

- Acute disseminated encephalomyelitis, including site-specific variants: eg, non-infectious encephalitis, encephalomyelitis, myelitis, myeloradiculomyelitis
- Myasthenia gravis and Lambert-Eaton myasthenic syndrome
- Immune-mediated peripheral neuropathies and plexopathies (including chronic inflammatory demyelinating polyneuropathy, multifocal motor neuropathy and polyneuropathies associated with monoclonal gammopathy)
- Narcolepsy
- Isolated paresthesia of >7 days duration

Symptoms, signs or conditions which might (or might not) represent the above diagnoses, should be recorded and reported as IREs even if the final or definitive diagnosis has not yet been determined, and alternative diagnoses have not yet been eliminated or shown to be less likely. Follow up information and final diagnoses, if applicable, should be submitted as soon as they become available.

If the IRE is also serious (serious adverse event), it will be reported using the same process as for other serious adverse events.

12.1.2. Attribution Definitions

Every effort should be made by the investigator to explain any adverse event and to assess its potential causal relationship, ie, to administration of the study vaccine or to alternative causes (eg, natural history of an underlying diseases, concomitant therapies). This applies to all adverse events, whether serious or non-serious.

Symptoms of local adverse events (injection site reactions) will be considered as related to the study vaccine. For all other adverse events, the investigator will use the following guidelines to assess the causal relationship to study vaccine:

Not Related

An adverse event that is not related to the use of the vaccine.

Doubtful

An adverse event for which an alternative explanation is more likely, eg, concomitant drug(s), concomitant disease(s), or the relationship in time suggests that a causal relationship is unlikely.

Possible

An adverse event that might be due to the use of the vaccine. An alternative explanation, eg, concomitant drug(s), concomitant disease(s), is inconclusive. The relationship in time is reasonable; therefore, the causal relationship cannot be excluded.

Probable

An adverse event that might be due to the use of the vaccine. The relationship in time is suggestive (eg, confirmed by dechallenge). An alternative explanation is less likely, eg, concomitant drug(s), concomitant disease(s).

Very Likely

An adverse event that is listed as a possible adverse reaction and cannot be reasonably explained by an alternative explanation, eg, concomitant drug(s), concomitant disease(s). The relationship in time is very suggestive.

12.1.3. Severity Criteria

Adverse events and laboratory data will be coded for severity using the toxicity grading tables in Attachment 4 and Attachment 5. For adverse events not identified in the table, the following guidelines will apply:

**Mild**	Grade 1	Symptoms causing no or minimal interference with usual social and functional activities
**Moderate**	Grade 2	Symptoms causing greater than minimal interference with usual social and functional activities
**Severe**	Grade 3	Symptoms causing inability to perform usual social and functional activities

Note: Only clinically significant abnormalities in laboratory data occurring from signing of the ICF onwards will be reported as adverse events and graded using the table above.

12.2. Special Reporting Situations

Safety events of interest on a sponsor study drug or vaccine that may require expedited reporting and/or safety evaluation include, but are not limited to:

- Overdose of a sponsor study vaccine
- Inadvertent or accidental exposure to a sponsor study vaccine
- Medication error involving a sponsor product (with or without subject/patient exposure to the sponsor study vaccine, eg, name confusion)
- IREs

Special reporting situations should be recorded in the CRF. For reporting of IREs, refer to Section 12.3.3. Any special reporting situation that meets the criteria of a serious adverse event should be recorded on the serious adverse event page of the CRF.

12.3. Procedures

12.3.1. All Adverse Events

All adverse events and special reporting situations, whether serious or non-serious, will be reported from the time a signed and dated ICF is obtained until 56 days after the boost vaccination in Stage 1 and then again from the day of the third vaccination until 28 days after the third vaccination, and until 28 days after the prime vaccination in Stage 2 and then again until 28 days after the boost vaccination. Subjects in Stage 1 and Stage 2 will also record symptoms of solicited local and systemic adverse events in the diary in the evening after the prime and boost vaccination and after the third vaccination (Stage 1 only) and then daily for the next 7 days.

Serious adverse events, including deaths, and IREs will be followed-up until the subject's last study visit.

Serious adverse events must be reported using the Serious Adverse Event Form. SUSARs will be reported even after the study is over, if the sponsor, the IDMC or the investigator becomes aware of them.

The sponsor will evaluate any safety information that is spontaneously reported by an investigator beyond the time frame specified in the protocol.

The investigator will monitor and analyze the study data including all adverse event and clinical laboratory data as they become available and will make determinations regarding the severity of the adverse experiences and their relation to study vaccine. All adverse events will be deemed related to study vaccine or not related to study vaccine, according to Section 12.1.2.

The investigator or clinical designee must review both post-injection reactogenicity and other adverse event CRFs to insure the prompt and complete identification of all events that require expedited reporting as serious adverse events, invoke pausing rules or are other serious and unexpected events.

All adverse events, regardless of seriousness, severity, or presumed relationship to study vaccine, must be recorded using medical terminology in the source document and the CRF. Whenever possible, diagnoses should be given when signs and symptoms are due to a common etiology (eg, cough, runny nose, sneezing, sore throat, and head congestion should be reported as "upper respiratory infection"). Investigators must record in the CRF their opinion concerning the relationship of the adverse event to study therapy. All measures required for adverse event management must be recorded in the source document and reported according to sponsor instructions. For reporting of IREs, refer to Section 12.3.3.

The sponsor assumes responsibility for appropriate reporting of adverse events to the regulatory authorities. The sponsor will also report to the investigator (and the head of the investigational institute where required) all SUSARs. The investigator (or sponsor where required) must report SUSARs to the appropriate IEC/IRB that approved the protocol unless otherwise required and documented by the IEC/IRB. A SUSAR will be reported to regulatory authorities unblinded

(Stage 2 only). Participating investigators and IEC/IRB will receive a blinded SUSAR summary, unless otherwise specified.

Subjects will be provided with a wallet card and instructed to carry this card with them for the duration of the study indicating the following:

- Study number
- Statement, in the local language(s), that the subject is participating in a clinical study
- Investigator's name and 24-hour contact telephone number
- Sponsor's name and 24-hour contact telephone number (for medical staff only)
- Subject number
- Information about who should be contacted in case of emergency
- Any other information that is required to do an emergency breaking of the blind (Stage 2)
- Ebola prevention counseling

12.3.2. Serious Adverse Events

All serious adverse events occurring during the study must be reported to the appropriate sponsor contact person by study-site personnel within 24 hours of their knowledge of the event. All serious adverse events will be reported in accordance with local regulations.

Information regarding serious adverse events will be transmitted to the sponsor using the Serious Adverse Event Form, which must be completed and signed by a physician from the study site, and transmitted to the sponsor within 24 hours. The initial and follow-up reports of a serious adverse event should be provided according to the sponsor’s instructions.

All serious adverse events that have not resolved by the end of the study, or that have not resolved upon discontinuation of the subject's participation in the study, must be followed until any of the following occurs:

- The event resolves
- The event stabilizes
- The event returns to baseline, if a baseline value/status is available
- The event can be attributed to agents other than the study vaccine or to factors unrelated to study conduct
- It becomes unlikely that any additional information can be obtained (subject or health care practitioner refusal to provide additional information, lost to follow-up after demonstration of due diligence with follow-up efforts)

Suspected transmission of an infectious agent by a medicinal product will be reported as a serious adverse event. Any event requiring hospitalization (or prolongation of hospitalization) that occurs during the course of a subject's participation in a study must be reported as a serious adverse event, except hospitalizations for the following:

- Hospitalizations not intended to treat an acute illness or adverse event (eg, social reasons such as pending placement in long-term care facility)
- Surgery or procedure planned before entry into the study (must be documented in the CRF). *Note: Hospitalizations that were planned before the signing of the ICF, and where the underlying condition for which the hospitalization was planned has not worsened, will not be considered serious adverse events. Any adverse event that results in a prolongation of the originally planned hospitalization is to be reported as a new serious adverse event.*

During the entire study, the cause of death of a subject, whether or not the event is expected or associated with the study vaccine, is considered a serious adverse event.

12.3.3. Immediate Reportable Events

Immediate Reportable Events

One subject in the study VAC52150EBL2001 experienced a serious and very rare condition called "Miller Fisher syndrome". This condition consists of double vision, pain on moving the eye, and difficulty with balance while walking. Miller Fisher syndrome most commonly occurs following a recent infection. The subject experienced these symptoms about a week after suffering from a common cold and fever. The event happened about a month after boost vaccination with either MVA-BN-Filo or placebo. This subject had to go to the hospital for treatment and has recovered. After an extensive investigation, the event has been considered to be doubtfully related to vaccine and most likely related to the previous common cold.

Any events of neuroimmunologic significance (listed below) should be categorized as IREs and should be reported throughout the study using the IRE Form provided **within 24 hours to the sponsor**. Events suggestive of the disorders considered IREs should be reported even if the final diagnosis has not been yet determined, and follow-up information and final diagnosis should be submitted to the sponsor as soon as they become available.

If an event meets criteria for serious adverse events (see above), it should be documented as such using the Serious Adverse Event form, as well as the relevant CRF Adverse Event page and the complete IRE Form per the instructions to be included as part of the Serious Adverse Event report.

12.3.4. Pregnancy

All initial reports of pregnancy must be reported to the sponsor by the study-site personnel within 24 hours of their knowledge of the event using the appropriate pregnancy notification form. Abnormal pregnancy outcomes (eg, spontaneous abortion, fetal death, stillbirth, congenital anomalies, ectopic pregnancy) are considered serious adverse events and must be reported using the Serious Adverse Event Form. Any subject who becomes pregnant during the study must be promptly withdrawn from further study vaccination but should continue participation in the study for follow-up of safety and immunogenicity if this does not result in a safety risk for the subject.

Follow-up information regarding the outcome of the pregnancy and any postnatal sequelae in the infant will be required.

Pregnancy outcomes from all female subjects who became pregnant during the study until up to 28 days after the boost vaccination or 3 months after the prime vaccination (whichever is longer) as well as from those who became pregnant between at least 14 days before until up to 3 months after the third vaccination may be collected in the current study or in the VAC52150EBL3005 long-term follow-up study for subjects who consent to this. Children born to these female subjects may be enrolled in VAC52150EBL3005 study and followed for 5 years if the parent consents to this.

12.4. Contacting Sponsor Regarding Safety

The names (and corresponding telephone numbers) of the individuals who should be contacted regarding safety issues or questions regarding the study are listed on the Contact Information page(s), which will be provided as a separate document.

13. PRODUCT QUALITY COMPLAINT HANDLING

A product quality complaint (PQC) is defined as any suspicion of a product defect related to manufacturing, labeling, or packaging, ie, any dissatisfaction relative to the identity, quality, durability, or reliability of a product, including its labeling or package integrity. A PQC may have an impact on the safety and efficacy of the product. Timely, accurate, and complete reporting and analysis of PQC information from studies are crucial for the protection of subjects, investigators, and the sponsor, and are mandated by regulatory agencies worldwide. The sponsor has established procedures in conformity with regulatory requirements worldwide to ensure appropriate reporting of PQC information; all studies conducted by the sponsor or its affiliates will be conducted in accordance with those procedures.

13.1. Procedures

All initial PQCs must be reported to the sponsor by the study-site personnel within 24 hours after being made aware of the event.

If the defect is combined with a serious adverse event, the study-site personnel must report the PQC to the sponsor according to the serious adverse event reporting timelines (refer to Section 12.3.2). A sample of the suspected product should be maintained for further investigation if requested by the sponsor.

13.2. Contacting Sponsor Regarding Product Quality

The names (and corresponding telephone numbers) of the individuals who should be contacted regarding product quality issues are listed on the Contact Information page(s), which will be provided as a separate document.

14. STUDY VACCINE INFORMATION

14.1. Description of Study Vaccines

14.1.1. Ad26.ZEBOV

Ad26.ZEBOV is a monovalent, replication-incompetent Ad26-based vector that expresses the full length EBOV Mayinga GP and is produced in the human cell line PER.C6®.

The Ad26.ZEBOV vaccine will be supplied at a concentration of $1x10^{11}$ vp/mL in 2-mL single-use glass vials as a frozen liquid to be thawed before use. Each vial contains an extractable volume of 0.5 mL. Refer to the Investigator's Brochure for a list of excipients.[8]

The Ad26.ZEBOV vaccine is manufactured by IDT Biologika GmbH for Janssen Vaccines & Prevention B.V., The Netherlands.

14.1.2. MVA-BN-Filo

MVA-BN-Filo is a recombinant multivalent vaccine intended for active immunization against Ebola and Marburg virus infection. MVA-BN-Filo is strongly attenuated; the vaccine is propagated in primary chicken embryo fibroblast (CEF) cells and does not replicate in human cells.

The MVA-BN-Filo vaccine is supplied at a concentration of 2×10^8 Inf U/mL in 2-mL single-use glass vials as a frozen liquid suspension to be thawed before use. Each vial contains an extractable volume of 0.5 mL. Refer to the Investigator's Brochure for a list of excipients.[9]

The MVA-BN-Filo vaccine is manufactured by IDT Biologika GmbH for Janssen Vaccines & Prevention B.V., The Netherlands.

14.1.3. MenACWY

MenACWY is a WHO-prequalified Meningococcal Group A, C, W135 and Y conjugate vaccine.

The MenACWY vaccines will be supplied as commercially available vaccines. Refer to the SPC for a list of excipients.

14.1.4. Placebo

The placebo supplied for this study will be formulated as a sterile 0.9% saline for injection (as commercially available).

14.2. Packaging and Labeling

All study vaccines will be manufactured and packaged in accordance with Good Manufacturing Practice (GMP). All study vaccines will be packaged and labeled under the responsibility of the sponsor. No study vaccine can be repacked or relabeled without prior approval from the sponsor.

Further details for study vaccine packaging and labeling can be found in the Site Investigational Product Procedures Manual.

14.3. Preparation, Handling and Storage

Study vaccine must be stored at controlled temperatures. Guidance on storage temperature is provided in the Site Investigational Product Procedures Manual.

Vials must be stored in a secured location with no access for unauthorized personnel. All study product storage equipment (including refrigerators, freezers) must be equipped with a continuous temperature monitor and alarm, and with back-up power systems. In the event that study vaccine is exposed to temperatures outside the specified temperature ranges, all relevant data will be sent to the sponsor to determine if the affected study vaccine can be used or will be replaced. The affected study vaccine must be quarantined and not used until further instruction from the sponsor is received.

A pharmacist/qualified staff member will prepare all doses for vaccine administration and provide it for dispensing. Blinding in Stage 2 will be achieved by preparation of study vaccine by unblinded qualified study-site personnel not involved in any other study-related procedures, and by the administration of vaccine in a masked syringe in a way that maintains double-blinding.

Full details on the preparation, the holding time and storage conditions from the time of preparation to delivery of Ad26.ZEBOV and MVA-BN-Filo and active control are provided in the Site Investigational Product Procedures Manual and Site Blinding Plan (for Stage 2 only).

14.4. Vaccine Accountability

The investigator is responsible for ensuring that all study vaccine received at the site is inventoried and accounted for throughout the study. The study vaccine administered to the subject must be documented on the study vaccine accountability form. All study vaccine will be stored and disposed of according to the sponsor’s instructions. Study-site personnel must not combine contents of the study vaccine containers.

Study vaccine must be handled in strict accordance with the protocol and the container label, and must be stored at the study site in a limited-access area or in a locked cabinet under appropriate environmental conditions. Unused study vaccine must be available for verification by the sponsor’s study site monitor during on-site monitoring visits. The return to the sponsor of unused study vaccine will be documented on the Investigational Product Destruction Form. When the study site is an authorized destruction unit and study vaccine supplies are destroyed on-site, this must also be documented on the Investigational Product Destruction Form.

Potentially hazardous materials, such as used needles, syringes, empty vials, and vials containing hazardous liquids, should be disposed of immediately in a safe manner and therefore will not be retained for study vaccine accountability purposes.

Study vaccine will be dispensed under the supervision of the investigator or qualified member of the study-site personnel, or by the pharmacist/qualified staff member. Study vaccine will be supplied only to subjects participating in the study. Study vaccine may not be reassigned for use by other subjects. Applying additional labels can only be done on the authority of the sponsor. The

investigator agrees neither to dispense the study vaccine from, nor store it at, any site other than the study sites agreed upon with the sponsor.

15. STUDY-SPECIFIC MATERIALS

The investigator will be provided with the following supplies:

- Investigator Brochures and Addendum (if applicable) for Ad26.ZEBOV and MVA-BN-Filo
- Site Investigational Product Procedures Manual and Site Blinding Plan (for Stage 2 only)
- Laboratory Manual
- Algorithm for evaluation of fever
- Interactive Voice/Web Response System (IVRS/IWRS) Manual (Stage 1 third vaccination and Stage 2)
- Electronic Data Capture (eDC) Manual/electronic CRF Completion Guidelines
- Deployment and Compliance manual
- Sample ICF
- Subject diaries
- TOU
- Rulers, thermometers
- Recruitment tools, as applicable
- Subject wallet card

16. ETHICAL ASPECTS

16.1. Study-Specific Design Considerations

Potential subjects will be fully informed of the risks and requirements of the study and, during the study, subjects will be given any new information that may affect their decision to continue participation. They will be told that their consent to participate in the study is voluntary and may be withdrawn at any time with no reason given and without penalty or loss of benefits to which they would otherwise be entitled. Only subjects who are fully able to understand the risks, benefits, and potential adverse events of the study, and provide their consent voluntarily will be enrolled.

When referring to the signing of the ICF, the terms legal guardian and legally acceptable representative refer to the traditionally or legally appointed guardian of the child with authority to authorize participation in research. For each subject, his or her parent(s) (preferably both parents, if available) or a legally acceptable representative(s), as required by local regulations, must give written consent (permission) according to local requirements after the nature of the study has been fully explained and before the performance of any study-related assessments. Assent must be obtained from children (minors) capable of understanding the nature of the study, typically subjects 7 years of age and older, depending on the institutional policies. For the purposes of this study, all references to subjects who have provided consent (and assent as applicable) refers to the

subjects and his or her parent(s) or the subject's legal guardian(s) or legally acceptable representative(s) who have provided consent according to this process. Minors who assent to a study and later withdraw that assent should not be maintained in the study against their will, even if their parents still want them to participate. For details of the consent procedures in this study, refer to Section 16.2.3.

The total blood volume to be collected is expected to be approximately between 28.1 and 164.4 mL, depending on the stage of the study, and the age of the subject. Details are provided in the Time and Events Schedules.

Test of Understanding

The TOU is a short assessment of the subject's understanding of key aspects of the study. The test will help the study staff to determine how well the subject understands the study and the requirements for participation.

The TOU must be completed by all subjects participating in Stage 1 and Stage 2, prior to enrollment into the study and after reading but before signing the ICF. For children, the TOU will be administered to the parent or guardian from whom consent is sought. If both the parent or guardian and the child will be enrolled in the study, the parent/guardian will need to complete the TOU twice (ie, one for the parent/guardian and one for the child). In case the parent/guardian consents for multiple children of the same family, the TOU needs to be completed only once, ie, for the first child of the family enrolled in the study. In case the TOU information would change while recruiting multiple children of the same family, the TOU should be completed again for the first child of the family that was enrolled after the TOU change had occurred. The TOU is reviewed one-on-one with the subject and a member of the study team. Subjects in Stage 2 are allowed to retake the test twice to achieve the passing score (≥90%) required for participation in the study. If a subject fails to achieve the passing score, further information and counseling will be provided by the study team member.

Any subject not capable of understanding the key aspects of the study, and their requirements for participation, should not be enrolled.

16.2. Regulatory Ethics Compliance

16.2.1. Investigator Responsibilities

The investigator is responsible for ensuring that the study is performed in accordance with the protocol, current ICH guidelines on GCP, and applicable regulatory and country-specific requirements.

Good Clinical Practice is an international ethical and scientific quality standard for designing, conducting, recording, and reporting studies that involve the participation of human subjects. Compliance with this standard provides public assurance that the rights, safety, and well-being of study subjects are protected, consistent with the principles that originated in the Declaration of Helsinki, and that the study data are credible.

16.2.2. Independent Ethics Committee or Institutional Review Board

Before the start of the study, the investigator (or sponsor where required) will provide the IEC/IRB with current and complete copies of the following documents (as required by local regulations):

- Final protocol and, if applicable, amendments
- Sponsor-approved ICF (and any other written materials to be provided to the subjects)
- Investigator's Brochure (or equivalent information) and amendments/addenda
- Subject diaries
- TOU
- Sponsor-approved subject recruiting materials
- Information on compensation for study-related injuries or payment to subjects for participation in the study, if applicable
- Investigator's curriculum vitae or equivalent information (unless not required, as documented by the IEC/IRB)
- Information regarding funding, name of the sponsor, institutional affiliations, other potential conflicts of interest, and incentives for subjects
- Any other documents that the IEC/IRB requests to fulfill its obligation

This study will be undertaken only after the IEC/IRB has given full approval of the final protocol, amendments (if any, excluding the ones that are purely administrative, with no consequences for subjects, data or study conduct), the ICF, applicable recruiting materials, and subject compensation programs, and the sponsor has received a copy of this approval. This approval letter must be dated and must clearly identify the IEC/IRB and the documents being approved.

During the study the investigator (or sponsor where required) will send the following documents and updates to the IEC/IRB for their review and approval, where appropriate:

- Protocol amendments (excluding the ones that are purely administrative, with no consequences for subjects, data or study conduct)
- Revision(s) to ICF and any other written materials to be provided to subjects
- If applicable, new or revised subject recruiting materials approved by the sponsor
- Revisions to compensation for study-related injuries or payment to subjects for participation in the study, if applicable
- New edition(s) of the Investigator's Brochure and amendments/addenda
- Summaries of the status of the study at intervals stipulated in guidelines of the IEC/IRB (at least annually)
- Reports of adverse events that are serious, unlisted/unexpected, and associated with the study vaccine
- New information that may adversely affect the safety of the subjects or the conduct of the study

- Deviations from or changes to the protocol to eliminate immediate hazards to the subjects
- Report of deaths of subjects under the investigator's care
- Notification if a new investigator is responsible for the study at the site
- Development Safety Update Report and Line Listings, where applicable
- Any other requirements of the IEC/IRB

For all protocol amendments (excluding the ones that are purely administrative, with no consequences for subjects, data or study conduct), the amendment and applicable ICF revisions must be submitted promptly to the IEC/IRB for review and approval before implementation of the change(s).

At least once a year, the IEC/IRB will be asked to review and reapprove this study. The reapproval should be documented in writing (excluding the ones that are purely administrative, with no consequences for subjects, data, or study conduct).

At the end of the study, the investigator (or sponsor where required) will notify the IEC/IRB about the study completion.

16.2.3. Informed Consent

In this community-based, study, informed consent will take place at several levels ranging from approval from the government authorities, followed by community engagement, and finally, individual consent.

Approval from Respective Authorities

Approval for this project will be obtained from the respective healthcare authorities in the country where the study is conducted.

Additionally, during the planning process of the study approval will be sought from other authorities such as district or local councilors, political leaders, and traditional leaders.

Engagement at the Community Level

Documented community engagement by a community leader must be available.

Consent at the Individual Level

Consent at the individual level will be sought from all subjects who participate in Stage 1 and Stage 2.

Each subject (or a legally acceptable representative) must give written consent according to local requirements after the nature of the study has been fully explained. The ICF(s) must be signed before performance of any study-related activity. The ICF(s) that is/are used must be approved by both the sponsor and by the reviewing IEC/IRB and be in a language that the subject can understand. The informed consent should be in accordance with principles that originated in the

Declaration of Helsinki, current ICH and GCP guidelines, applicable regulatory requirements, and sponsor policy.

Before enrollment in the study, the investigator or an authorized member of the study-site personnel must explain to potential subjects or their legally acceptable representatives the aims, methods, reasonably anticipated benefits, and potential hazards of the study, and any discomfort participation in the study may entail. Subjects will be informed that their participation is voluntary and that they may withdraw consent to participate at any time. They will be informed that choosing not to participate will not affect the care the subject will receive for the treatment of his or her disease. Finally, they will be told that the investigator will maintain a subject identification register for the purposes of long-term follow up if needed and that their records may be accessed by health authorities and authorized sponsor personnel without violating the confidentiality of the subject, to the extent permitted by the applicable law(s) or regulations. By signing the ICF the subject or legally acceptable representative is authorizing such access, including permission to obtain information about his or her survival status, and agrees to allow his or her study physician to recontact the subject for the purpose of obtaining consent for additional safety evaluations, if needed.

The subject or legally acceptable representative will be given sufficient time to read the ICF and the opportunity to ask questions. After this explanation and before entry into the study, consent should be appropriately recorded by means of either the subject's or his or her legally acceptable representative's personally dated signature. After having obtained the consent, a copy of the ICF must be given to the subject.

For subjects in Stage 1 and Stage 2, a TOU will be administered after reading but before signing the ICF, to assess the subject’s understanding of key aspects of the study.

Children (minors) or subjects who are unable to comprehend the information provided can be enrolled only after obtaining consent of a legally acceptable representative. Assent must be obtained from children (minors) capable of understanding the nature of the study, typically subjects 7 years of age and older, depending on the institutional policies. The assent procedure must be witnessed by an adult literate parent/legal guardian/third party not involved in the conduct of the study, and documented.

Subjects enrolled in this study as minors and who are still actively participating in the study when they turn 18 years old will need to consent as adults for further participation in the study. Consenting will occur at the next available opportunity (ie, next study visit, contact point, intervention, etc) upon reaching majority. Similarly, subjects who turn 7 years old and who are still actively participating in the study will be requested to give a positive assent.

16.2.4. Privacy of Personal Data

The collection and processing of personal data from subjects enrolled in this study will be limited to those data that are necessary to fulfill the objectives of the study.

These data must be collected and processed with adequate precautions to ensure confidentiality and compliance with applicable data privacy protection laws and regulations. Appropriate technical and organizational measures to protect the personal data against unauthorized disclosures or access, accidental or unlawful destruction, or accidental loss or alteration must be put in place. Sponsor personnel whose responsibilities require access to personal data agree to keep the identity of subjects confidential.

The informed consent obtained from the subject (or his or her legally acceptable representative) includes explicit consent for the processing of personal data and for the investigator/institution to allow direct access to his or her original medical records (source data/documents) for study-related monitoring, audit, IEC/IRB review, and regulatory inspection. This consent also addresses the transfer of the data to other entities and to other countries with similar privacy protection security.

The subject has the right to request through the investigator access to his or her personal data and the right to request rectification of any data that are not correct or complete. Reasonable steps will be taken to respond to such a request, taking into consideration the nature of the request, the conditions of the study, and the applicable laws and regulations.

Exploratory research is not conducted under standards appropriate for the return of data to subjects. In addition, the sponsor cannot make decisions as to the significance of any findings resulting from exploratory research. Therefore, exploratory research data will not be returned to subjects or investigators, unless required by law or local regulations. Privacy and confidentiality of data generated in the future on stored samples will be protected by the same standards applicable to all other clinical data.

16.2.5. Long-Term Retention of Samples for Additional Future Research

Each subject in Stage 1 and 2 will be asked to consent voluntarily for their blood samples to be stored for other research studies that may be done after this study is completed. Subjects unwilling to have their blood samples stored for future use, can participate in the immunogenicity assessments without having their blood samples stored for future testing (see also Section 10.4). In such case, their blood samples will be destroyed after all the immunogenicity tests have been concluded (as agreed by the sponsor).

All samples, for which consent has been obtained and for which additional material is available after study-specified testing is complete, will be stored for future testing. A PCR test may be performed to test for presence of Ebola virus in the samples if samples need to be exported. Applicable approvals will be sought before any such samples are used for analysis not specified in the protocol or a protocol amendment approved by the IEC/IRB.

Stored samples will be coded throughout the sample storage and analysis process and will not be labeled with personal identifiers.

17. ADMINISTRATIVE REQUIREMENTS

17.1. Protocol Amendments

Neither the investigator nor the sponsor will modify this protocol without a formal amendment by the sponsor. All protocol amendments must be issued by the sponsor, and signed and dated by the investigator. Protocol amendments must not be implemented without prior IEC/IRB approval, or when the relevant competent authority has raised any grounds for non-acceptance, except when necessary to eliminate immediate hazards to the subjects, in which case the amendment must be promptly submitted to the IEC/IRB and relevant competent authority. Documentation of amendment approval by the investigator and IEC/IRB must be provided to the sponsor. When the change(s) involves only logistic or administrative aspects of the study, the IRB (and IEC where required) only needs to be notified.

During the course of the study, in situations where a departure from the protocol is unavoidable, the investigator or other physician in attendance will contact the appropriate sponsor representative (see Contact Information page(s) provided separately). Except in emergency situations, this contact should be made before implementing any departure from the protocol. In all cases, contact with the sponsor must be made as soon as possible to discuss the situation and agree on an appropriate course of action. The data recorded in the CRF and source documents will reflect any departure from the protocol, and the source documents will describe this departure and the circumstances requiring it.

17.2. Regulatory Documentation

17.2.1. Regulatory Approval/Notification

This protocol and any amendment(s) must be submitted to the appropriate regulatory authorities in each respective country, if applicable. A study may not be initiated until all local regulatory requirements are met.

17.2.2. Required Prestudy Documentation

The following documents must be provided to the sponsor before shipment of study vaccine to the study site:

- Protocol and amendment(s), if any, signed and dated by the principal investigator
- A copy of the dated and signed (or sealed, where appropriate per local regulations), written IEC/IRB approval of the protocol, amendments, ICF, any recruiting materials, and if applicable, subject compensation programs. This approval must clearly identify the specific protocol by title and number and must be signed (or sealed, where appropriate per local regulations) by the chairman or authorized designee.
- Name and address of the IEC/IRB, including a current list of the IEC/IRB members and their function, with a statement that it is organized and operates according to GCP and the applicable laws and regulations. If accompanied by a letter of explanation, or equivalent, from the IEC/IRB, a general statement may be substituted for this list. If an investigator or a member of the study-site personnel is a member of the IEC/IRB, documentation must be

obtained to state that this person did not participate in the deliberations or in the vote/opinion of the study.

- Regulatory authority approval or notification, if applicable
- Signed and dated statement of investigator (eg, Form FDA 1572), if applicable
- Documentation of investigator qualifications (eg, curriculum vitae)
- Completed investigator financial disclosure form from the principal investigator, where required
- Signed and dated clinical trial agreement, which includes the financial agreement
- Any other documentation required by local regulations

The following documents must be provided to the sponsor before enrollment of the first subject:

- Completed investigator financial disclosure forms from all subinvestigators
- Documentation of subinvestigator qualifications (eg, curriculum vitae)
- Name and address of any local laboratory conducting tests for the study, and a dated copy of current laboratory normal ranges for these tests, if applicable
- Local laboratory documentation demonstrating competence and test reliability (eg, accreditation/license), if applicable

17.3. Subject Identification, Enrollment, and Screening Logs

The investigator agrees to complete a subject identification and enrollment log to permit easy identification of each subject during and after the study. This document will be reviewed by the sponsor study-site contact for completeness.

The subject identification and enrollment log will be treated as confidential and will be filed by the investigator in the study file. To ensure subject confidentiality, no copy will be made. All reports and communications relating to the study will identify subjects by subject identification and date of birth.

The investigator must also complete a subject screening log, which reports on all subjects who were seen to determine eligibility for inclusion in the study.

17.4. Source Documentation

At a minimum, source documentation must be available for the following to confirm data collected in the CRF: subject identification, eligibility, and study identification; study discussion and date of signed informed consent; dates of visits; date of vaccination, results of safety parameters as required by the protocol; record of all solicited and unsolicited adverse events and follow-up of adverse events; concomitant medication; vaccine receipt/dispensing/return records; study vaccine administration information; and date of study completion and reason for early discontinuation of study vaccine or withdrawal from the study, if applicable.

In addition, the author of an entry in the source documents should be identifiable.

At a minimum, the type and level of detail of source data available for a subject should be consistent with that commonly recorded at the study site as a basis for standard medical care. Specific details required as source data for the study will be reviewed with the investigator before the study and will be described in the monitoring guidelines (or other equivalent document).

The subject diary and TOU for subjects in Stage 1 and Stage 2 will be considered source documents.

Information from the diary provided to subjects to record symptoms of solicited local and systemic adverse events until 7 days after the prime and boost vaccination (Stage 1 and Stage 2) and after the third vaccination (Stage 1), will be reviewed by the investigator or clinical designee to transcribe into the relevant parts of the CRF as described in the CRF Completion Guidelines.

Inclusion and exclusion criteria not requiring documented medical history must be verified at a minimum by subject interview or other protocol required assessment (eg, physical examination, laboratory assessment) and documented in the source documents.

17.5. Case Report Form Completion

Case report forms are planned to be provided for each subject in electronic format.

Electronic Data Capture (eDC) will be used for this study. The study data will be transcribed by study-site personnel from the source documents onto an electronic CRF or entered directly into the electronic CRF which then serves as source data, and transmitted in a secure manner to the sponsor within the timeframe agreed upon between the sponsor and the study site. The electronic file will be considered to be the CRF.

Worksheets may be used for the capture of some data to facilitate completion of the CRF. Any such worksheets will become part of the subject's source documentation. All data relating to the study must be recorded in CRFs prepared by the sponsor. Data must be entered into CRFs in English. Study site personnel must complete the CRF as soon as possible after a subject visit, and the forms should be available for review at the next scheduled monitoring visit.

All subjective measurements (eg, pain scale information or other questionnaires) will be completed by the same individual who made the initial baseline determinations whenever possible. The investigator must verify that all data entries in the CRFs are accurate and correct.

All CRF entries, corrections, and alterations must be made by the investigator or other authorized study-site personnel. If necessary, queries will be generated in the eDC tool.

If corrections to a CRF are needed after the initial entry into the CRF, this can be done in 3 different ways:

- Study site personnel can make corrections in the eDC tool at their own initiative or as a response to an auto query (generated by the eDC tool).
- Study site manager can generate a query for resolution by the study-site personnel.

- Clinical data manager can generate a query for resolution by the study-site personnel.

17.6. Data Quality Assurance/Quality Control

Steps to be taken to ensure the accuracy and reliability of data include the selection of qualified investigators and appropriate study sites, review of protocol procedures with the investigator and study-site personnel before the study, and periodic monitoring visits by the sponsor. Written instructions will be provided for collection, handling, storage, and shipment of samples.

Guidelines for CRF completion will be provided and reviewed with study-site personnel before the start of the study.

The sponsor will review CRFs for accuracy and completeness during on-site monitoring visits and after transmission to the sponsor; any discrepancies will be resolved with the investigator or designee, as appropriate. After upload of the data into the study database they will be verified for accuracy and consistency with the data sources.

17.7. Record Retention

In compliance with the ICH/GCP guidelines, the investigator/institution will maintain all CRFs and all source documents that support the data collected from each subject, as well as all study documents as specified in ICH/GCP Section 8, Essential Documents for the Conduct of a Clinical Trial, and all study documents as specified by the applicable regulatory requirement(s). The investigator/institution will take measures to prevent accidental or premature destruction of these documents.

Essential documents must be retained until at least 2 years after the last approval of a marketing application in an ICH region and until there are no pending or contemplated marketing applications in an ICH region or until at least 2 years have elapsed since the formal discontinuation of clinical development of the investigational product. These documents will be retained for a longer period if required by the applicable regulatory requirements or by an agreement with the sponsor. It is the responsibility of the sponsor to inform the investigator/institution as to when these documents no longer need to be retained.

If the responsible investigator retires, relocates, or for other reasons withdraws from the responsibility of keeping the study records, custody must be transferred to a person who will accept the responsibility. The sponsor must be notified in writing of the name and address of the new custodian. Under no circumstance shall the investigator relocate or dispose of any study documents before having obtained written approval from the sponsor.

If it becomes necessary for the sponsor or the appropriate regulatory authority to review any documentation relating to this study, the investigator/institution must permit access to such reports.

17.8. Monitoring

The sponsor will perform study site visits to monitor this study.

The sponsor will perform on-site monitoring once a month or as frequently as necessary. The monitor will record dates of the visits in a study site visit log that will be kept at the study site. The first post-initiation visit will be made as soon as possible after enrollment for Stage 1 and Stage 2 has begun. At these visits, the monitor will compare the data entered into the CRFs with the vaccination unit and/or clinic records (source documents). The nature and location of all source documents will be identified to ensure that all sources of original data required to complete the CRF are known to the sponsor and study-site personnel and are accessible for verification by the sponsor study-site contact. If electronic records are maintained at the study site, the method of verification must be discussed with the study-site personnel.

Direct access to source documentation (medical records) must be allowed for the purpose of verifying that the data recorded in the CRF are consistent with the original source data. Findings from this review of CRFs and source documents will be discussed with the study-site personnel. The sponsor expects that, during monitoring visits, the relevant study-site personnel will be available, the source documentation will be accessible, and a suitable environment will be provided for review of study-related documents. The monitor will meet with the investigator on a regular basis during the study to provide feedback on the study conduct.

In Stage 2, there will be independent monitoring of the pharmacy and preparation of study vaccines by an unblinded monitor (independent study vaccine monitor); regular monitors will be blinded.

In addition to on-site monitoring visits, remote contacts can occur. It is expected that during these remote contacts, study-site personnel will be available to provide an update on the progress of the study at the site.

17.9. Study Completion/Termination

17.9.1. Study Completion

The study is considered completed with the last visit for the last subject participating in either Stage 1 or Stage 2 of the study. The final data from the study site will be sent to the sponsor (or designee) after completion of the final subject assessment at that study site, in the time frame specified in the Clinical Trial Agreement.

17.9.2. Study Termination

The sponsor reserves the right to close the study site or terminate the study at any time for any reason at the sole discretion of the sponsor. Study sites will be closed upon study completion. A study site is considered closed when all required documents and study supplies have been collected and a study-site closure visit has been performed.

The investigator may initiate study-site closure at any time, provided there is reasonable cause and sufficient notice is given in advance of the intended termination.

Reasons for the early closure of a study site by the sponsor or investigator may include but are not limited to:

- Failure of the investigator to comply with the protocol, the requirements of the IEC/IRB or local health authorities, the sponsor's procedures, or GCP guidelines
- Inadequate recruitment of subjects by the investigator
- Discontinuation of further study vaccine development

17.10. On-Site Audits

Representatives of the sponsor's clinical quality assurance department may visit the study site at any time during or after completion of the study to conduct an audit of the study in compliance with regulatory guidelines and company policy. These audits will require access to all study records, including source documents, for inspection and comparison with the CRFs. Subject privacy must, however, be respected. The investigator and study-site personnel are responsible for being present and available for consultation during routinely scheduled study-site audit visits conducted by the sponsor or its designees.

Similar auditing procedures may also be conducted by agents of any regulatory body, either as part of a national GCP compliance program or to review the results of this study in support of a regulatory submission. The investigator should immediately notify the sponsor if he or she has been contacted by a regulatory agency concerning an upcoming inspection.

17.11. Use of Information and Publication

All information, including but not limited to information regarding Ad26.ZEBOV and MVA-BN-Filo or the sponsor's operations (eg, patent application, formulas, manufacturing processes, basic scientific data, prior clinical data, formulation information) supplied by the sponsor to the investigator and not previously published, and any data, including exploratory research data, generated as a result of this study, are considered confidential and remain the sole property of the sponsor and may be apportioned between the consortium members, if contemplated and as detailed in the Consortium Agreement. The investigator agrees to maintain this information in confidence and use this information only to accomplish this study, and will not use it for other purposes without the sponsor's prior written consent.

The investigator understands that the information developed in the study will be used by the sponsor in connection with the continued development of Ad26.ZEBOV and MVA-BN-Filo, and thus may be disclosed as required to other clinical investigators or regulatory agencies. To permit the information derived from the clinical studies to be used, the investigator is obligated to provide the sponsor with all data obtained in the study.

The results of the study will be reported in a Clinical Study Report generated by the sponsor and will contain CRF data from all study sites that participated in the study. Recruitment performance or specific expertise related to the nature and the key assessment parameters of the study will be used to determine a coordinating investigator. Results of exploratory analyses performed after the Clinical Study Report has been issued will be reported in a separate report and will not require a revision of the Clinical Study Report. Study subject identifiers will not be used in publication of results. Any work created in connection with performance of the study and contained in the data that can benefit from copyright protection (except any publication by the investigator as provided

for below) shall be the property of the sponsor as author and owner of copyright in such work. Further details regarding ownership and access rights by consortium members to the data and results of the study are detailed in the Consortium Agreement.

Consistent with Good Publication Practices and International Committee of Medical Journal Editors guidelines, the sponsor and consortium members shall have the right to publish such primary (multicenter) data and information as detailed in the Consortium Agreement without approval from the individual investigators. The individual investigators have the right to publish study site-specific data after the primary data are published. Further details regarding publications by consortium members and individual investigators are detailed in the Clinical Trial Agreement and Consortium Agreement. The relevant publication sections of the Consortium Agreement will be shared with the investigator once executed by all the consortium partners. For multicenter study designs and sub-study approaches, secondary results generally should not be published before the primary endpoints of a study have been published. Similarly, consortium members and investigators will recognize the integrity of a multicenter study by not submitting for publication data derived from the individual study site until the combined results from the completed study have been submitted for publication, within 12 months of the availability of the final data (tables, listings, graphs), or the sponsor confirms there will be no multicenter study publication. Authorship of publications resulting from this study will be based on the guidelines on authorship, such as those described in the Consortium Agreement and the Uniform Requirements for Manuscripts Submitted to Biomedical Journals, which state that the named authors must have made a significant contribution to the design of the study or analysis and interpretation of the data, provided critical review of the paper, and given final approval of the final version.

Registration of Clinical Studies and Disclosure of Results

The sponsor will register and/or disclose the existence of and the results of clinical studies as required by law.

REFERENCES

1. Baize S, Pannetier D, Oestereich L, et al. Emergence of Zaire Ebola virus disease in Guinea. N Engl J Med. 2014;371(15):1418-1425.
2. Centers for Disease Control and Prevention. Clinical Growth Chart. Available at: htttp://www.cdc.gov/growthcharts/clinical_charts.htm. Accessed 10 March 2015.
3. Dosoo DK, Kayan K, Adu-Gyasi D et al. Haematological and biochemical reference values for healthy adults in the middle belt of Ghana. PLoS One. 2012;7(4):e36308.
4. Elizaga ML, Vasan S, Marovich MA, et al.; MVA Cardiac Safety Working Group. Prospective surveillance for cardiac adverse events in healthy adults receiving modified vaccinia Ankara vaccines: a systematic review. PLoS One. 2013;8(1):e54407.
5. Ethical Considerations for Clinical Trials on Medicinal Products Conducted With the Pediatric Population: Recommendations of the Ad Hoc Group for the Development of Implementing Guidelines for Directive 2001/20/EC Relating to Good Clinical Practice in the Conduct of Clinical Trials on Medicinal Products for Human Use. 2008.
6. Friedrich B, Trefry J, Biggins J, et al. Potential vaccines and post-exposure treatments for filovirus infections. Viruses. 2012;4(9):1619-1650.
7. IMVANEX suspension for injection. UK Summary of Product Characteristics. Available at: http://www.ema.europa.eu/docs/en_GB/document_library/EPAR_-_Product_Information/human/002596/WC500147896.pdf. Accessed 9 October 2014.
8. Investigator's Brochure: JNJ-61210474 (Ad26.ZEBOV) Edition 3. Crucell Holland B.V. (Oct 2015). For updated information, refer to Edition 4 (Janssen Vaccines & Prevention, 23 June 2016).
9. Investigator's Brochure: MVA-BN-Filo (MVA-mBN226B) Edition 6. Bavarian Nordic A/S (Nov 2015). For updated information, refer to Edition 10 (11 July 2016).
10. Neff J, Modlin J, Birkhead GS, et al. Monitoring the safety of a smallpox vaccination program in the United States: report of the joint Smallpox Vaccine Safety Working Group of the advisory committee on immunization practices and the Armed Forces Epidemiological Board. Clin Infect Dis. 2008;46 Suppl.3:S258-S270.
11. Stittelaar KJ, Kuiken T, de Swart RL et al. Safety of Modified Vaccinia Virus Ankara (MVA) in immune-suppressed macaques. Vaccine. 2001;19(27):3700-3709.
12. Verheust C, Goossens M, Pauwels K, Breyer D. Biosafety aspects of Modified Vaccinia Virus Ankara (MVA)-based vectors used for gene therapy or vaccination. Vaccine. 2012 30;30(16):2623-2632.
13. WHO Child Growth Standards (birth to 24 months). Available at: http://www.who.int/childgrowth/en. Accessed 10 March 2015.
14. WHO Fact Sheet Nº103 Ebola Virus Disease 2014. Available at: http://www.who.int/mediacentre/factsheets/fs103/en/. Accessed 9 October 2014.
15. WHO Global Health Observatory data – meningitis 2010. Available at: http://www.who.int/gho/epidemic_diseases/meningitis/suspected_cases_deaths_text/en/. Accessed 27 February 2017.
16. WHO Health Statistics and Information Systems: Disease and Injury Country Estimates. Available at: http://www.who.int/healthinfo/global_burden_disease/estimates_country/en/. Accessed 27 February 2017

ATTACHMENTS

Attachment 1: Schematic Presentation of the IDMC Schedule for the Prime and Boost Vaccination

The IDMC schedule applies to both the prime and the boost vaccination.

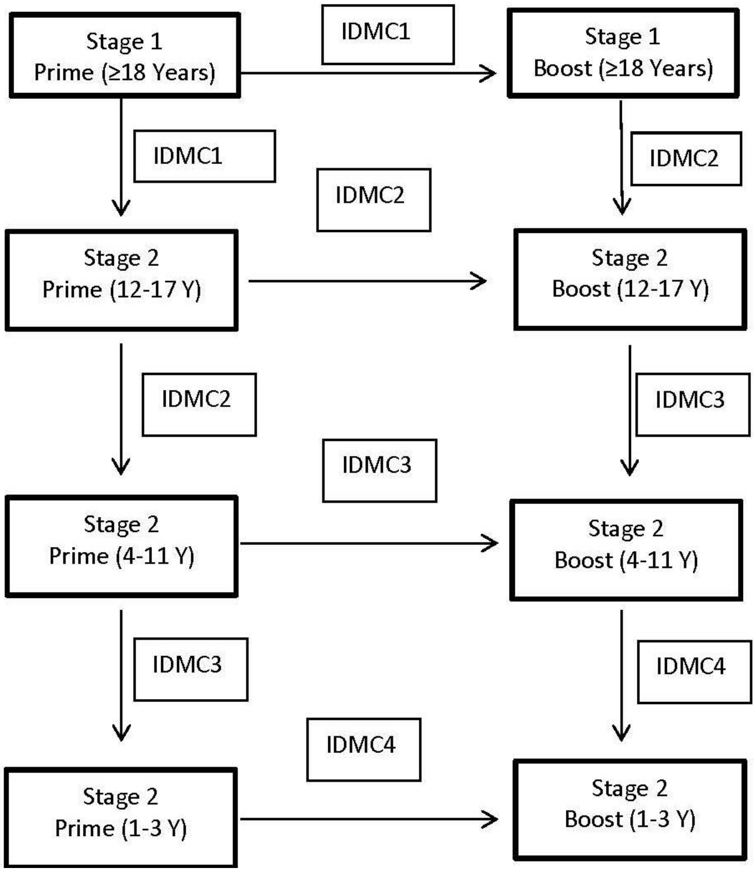

Attachment 2: Test of Understanding (TOU)

The TOU will be administered after reading but before signing the ICF.

Note: *a culturally appropriate translation will be made available to participating subjects.*

Please read each question and answer whether the statement is **True** or **False**.

True	False	
☐	☐	1. You will receive only one marklate as part of this study.
☐	☐	2. If you or your child are given the first marklate, you will be asked to come back 56 days later for the second marklate.
☐	☐	3. The marklates that you receive as part of this study definitely protect you against Ebola.
☐	☐	4. You will be asked to come back to the clinic twenty times as part of this study.
☐	☐	5. You may experience some side-effects after receiving the marklates.
☐	☐	6. If you feel unwell after receiving the marklates, you should call the emergency number on your wallet card.
☐	☐	7. Women participating in this study are permitted to become pregnant during the study.
☐	☐	8. You can change your mind at any time about taking part in the study.
☐	☐	9. The study staff are allowed to share information about your participation in the study with people who are not involved in the study.
☐	☐	10. If you agree to take part in this study, you must continue to protect yourself from Ebola by following Ebola prevention measures.

Attachment 3: Fever Management Algorithm

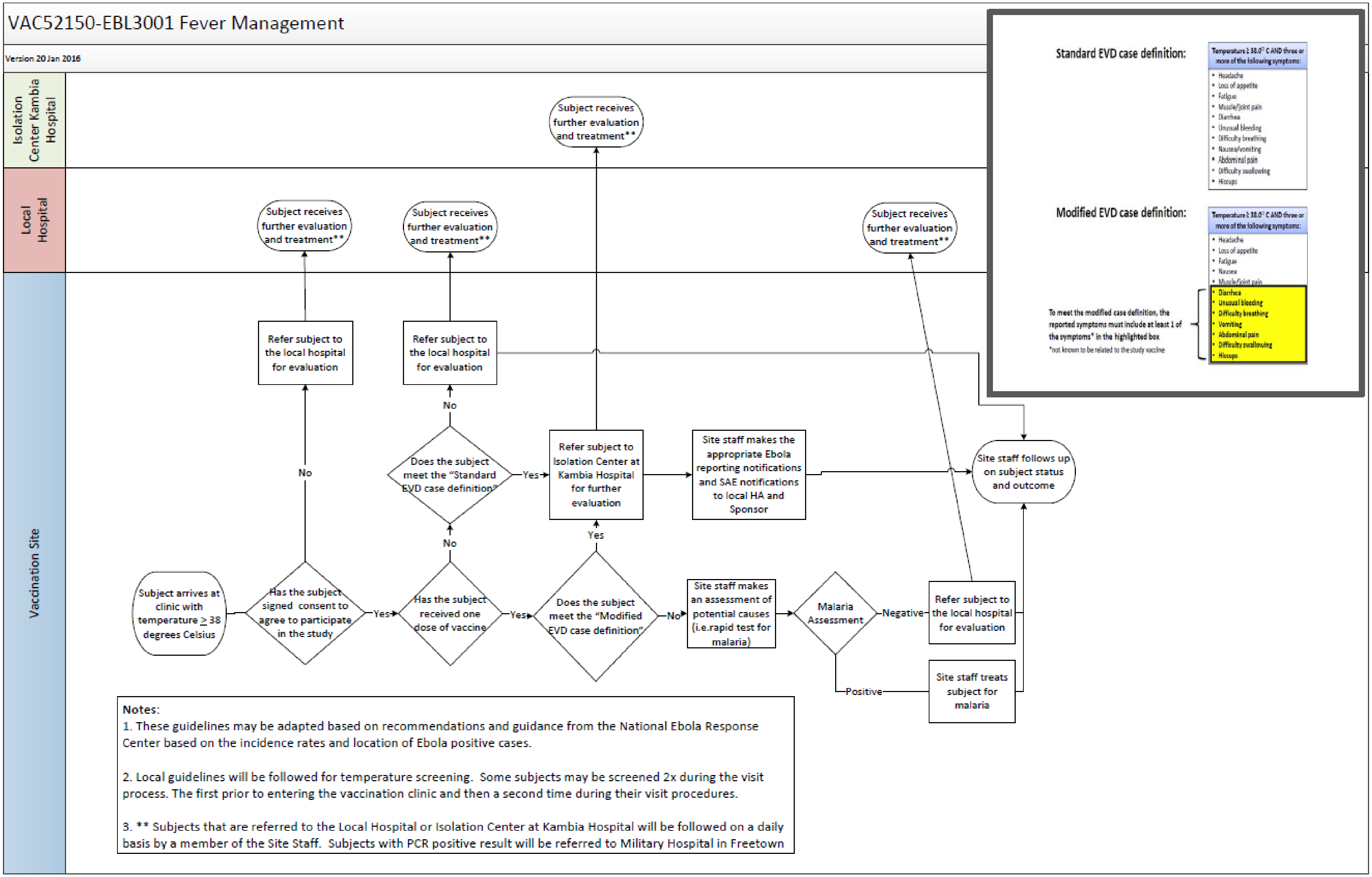

Attachment 4: Toxicity Tables for Use in Trials Enrolling Healthy Adults and Adolescents

The abbreviations used in the following tables are:

ALT: alanine aminotransferase; aPTT: activated partial thromboplastin time; AST: aspartate aminotransferase; AV block: atrioventricular block; bpm: beats per minute; CK: creatine kinase; FEV_1: forced expiratory volume in 1 second; g: gram; HI: high; HPF: high power field; INR: international normalized ratio; IV: intravenous; LO: low; mEq: milliequivalent; mm Hg: millimeter of mercury; N: not graded; PT: prothrombin time; PTT: partial thromboplastin time; QTc: QT-interval corrected for heart rate; QTcB: Bazett's corrected QT interval; QTcF: Fridericia's corrected QT interval; RBC: red blood cell; Rx: therapy; ULN: upper limit of normal

CLINICAL ADVERSE EVENTS

Grading scale used for clinical adverse events is adapted from the Division of Microbiology and Infectious Diseases (DMID) Toxicity Tables (2014). For adverse events not included in the tables below, refer to the severity criteria guidelines in Section 12.1.3.

Cardiovascular	Grade 1	Grade 2	Grade 3
Arrhythmia		Asymptomatic, transient signs, no Rx required	Recurrent/persistent; symptomatic Rx required
Hemorrhage, blood loss	Estimated blood loss ≤100 mL	Estimated blood loss >100 mL, no transfusion required	Transfusion required
QTcF (Fridericia's correction) [a] or QTcB (Bazett's correction)	Asymptomatic, QTc interval 450-479 ms, *OR* Increase in interval <30 ms above baseline	Asymptomatic, QTc interval 480-499 ms, *OR* Increase in interval 30-50 ms above baseline	Asymptomatic, QTc interval ≥500 ms, *OR* Increase in interval ≥60 ms above baseline
PR interval (prolonged)	PR interval 0.21-0.25 s	PR interval >0.25 s	Type II 2nd degree AV block *OR* Ventricular pause >3.0 s
**Respiratory**	**Grade 1**	**Grade 2**	**Grade 3**
Cough	Transient; no treatment	Persistent cough	Interferes with daily activities
Bronchospasm, acute	Transient; no treatment; FEV_1 71%-80% of peak flow	Requires treatment; normalizes with bronchodilator; FEV_1 60%-70% (of peak flow)	No normalization with bronchodilator; FEV_1 <60% of peak flow
Dyspnea	Does not interfere with usual and social activities	Interferes with usual and social activities, no treatment	Prevents daily and usual social activity or requires treatment

[a] Inclusion dependent upon protocol requirements.

Gastrointestinal	Grade 1	Grade 2	Grade 3
Nausea/vomiting	Minimal symptoms; caused minimal or no interference with work, school or self-care activities	Notable symptoms; required modification in activity or use of medications; did not result in loss of work or cancellation of social activities	Incapacitating symptoms; required bed rest and/or resulted in loss of work or cancellation of social activities
Diarrhea	2-3 loose or watery stools or <400 g/24 hours	4-5 loose or watery stools or 400-800 g/24 hours	6 or more loose or watery stools or >800 g/24 hours or requires IV hydration
**Reactogenicity**	**Grade 1**	**Grade 2**	**Grade 3**
Local reactions			
Pain/tenderness at injection site	Aware of symptoms but easily tolerated; does not interfere with activity; discomfort only to touch	Notable symptoms; required modification in activity or use of medications; discomfort with movement	Incapacitating symptoms; inability to do work or usual activities; significant discomfort at rest
Erythema/redness[a]	2.5-5 cm	5.1-10 cm	>10 cm
Induration/swelling[b]	2.5-5 cm and does not interfere with activity	5.1-10 cm or interferes with activity	>10 cm or prevents daily activity
Itching at the injection site	Minimal symptoms; caused minimal or no interference with work, school or self-care activities	Notable symptoms; required modification in activity or use of medications; did not result in loss of work or cancellation of social activities	Incapacitating symptoms; required bed rest and/or resulted in loss of work or cancellation of social activities
Systemic reactions			
Allergic reaction	Pruritus without rash	Localized urticaria	Generalized urticaria; angioedema or anaphylaxis
Headache	Minimal symptoms; caused minimal or no interference with work, school or self-care activities	Notable symptoms; required modification in activity or use of medications; did not result in loss of work or cancellation of social activities	Incapacitating symptoms; required bed rest and/or resulted in loss of work or cancellation of social activities
Fatigue/malaise	Minimal symptoms; caused minimal or no interference with work, school or self-care activities	Notable symptoms; required modification in activity or use of medications; did not result in loss of work or cancellation of social activities	Incapacitating symptoms; required bed rest and/or resulted in loss of work or cancellation of social activities
Myalgia	Minimal symptoms; caused minimal or no interference with work, school or self-care activities	Notable symptoms; required modification in activity or use of	Incapacitating symptoms; required bed rest and/or resulted in loss of work or cancellation of social activities

[a] In addition to grading the measured local reaction at the greatest single diameter, the measurement should be recorded as a continuous variable.
[b] Induration/swelling should be evaluated and graded using the functional scale by the investigator during diary review as well as the actual measurement documented by the subject in the diary.

		medications; did not result in loss of work or cancellation of social activities	
**Reactogenicity (continued)**	**Grade 1**	**Grade 2**	**Grade 3**
Arthralgia	Minimal symptoms; caused minimal or no interference with work, school or self-care activities	Notable symptoms; required modification in activity or use of medications; did not result in loss of work or cancellation of social activities	Incapacitating symptoms; required bed rest and/or resulted in loss of work or cancellation of social activities
Chills	Minimal symptoms; caused minimal or no interference with work, school or self-care activities	Notable symptoms; required modification in activity or use of medications; did not result in loss of work or cancellation of social activities	Incapacitating symptoms; required bed rest and/or resulted in loss of work or cancellation of social activities

LABORATORY TOXICITY GRADING

Grading scale used for laboratory assessments is based on 'FDA's Toxicity Grading Scale for Healthy Adult and Adolescent Volunteers Enrolled in Preventive Vaccine Clinical Trials', but grade 3 and 4 are pooled below, consistent with the 3 scale toxicity grading used throughout the protocol. If a laboratory value falls within the grading as specified below but also within the local laboratory normal limits, the value is considered as normal. For hemoglobin only the change from reference is used for the grading. The FDA table does not include toxicity grading for hematocrit, RBC counts or INR.

Blood, Serum, or Plasma Chemistries[a]	LO/HI/N [b]	Mild (Grade 1)	Moderate (Grade 2)	Severe (Grade 3)
Sodium (mEq/L or mmol/L)	LO	132-134	130-131	≤129
	HI	144-145	146-147	≥148
Potassium (mEq/L or mmol/L)	LO	3.5-3.6	3.3-3.4	≤3.2
	HI	5.1-5.2	5.3-5.4	≥5.5
Glucose (mg/dL)	LO	65-69	55-64	≤54
	HI [c]	100-110	111-125	>125
	HI [d]	110-125	126-200	>200
Blood urea nitrogen	HI	23-26 (mg/dL) or 8.3-9.4 (mmol/L)	27-31 (mg/dL) or 9.5- 11.2 (mmol/L)	>31 (mg/dL) or >11.2 (mmol/L)
Creatinine	N	1.5-1.7 (mg/dL) or 133-151 (µmol/L)	1.8-2.0 (mg/dL) or 159-177 (µmol/L)	>2.0 (mg/dL) or > 177 (µmol/L)
Calcium (mg/dL)	LO	8.0-8.4	7.5-7.9	<7.5
	HI	10.5-11.0	11.1-11.5	>11.5
Magnesium (mg/dL)	LO	1.3-1.5	1.1-1.2	<1.1
Phosphorus (mg/dL)	LO	2.3-2.5	2.0-2.2	<2.0
CK (mg/dL)	N	1.25-1.5 x ULN	1.6-3.0 x ULN	≥3.1 x ULN
Albumin (g/dL)	LO	2.8-3.1	2.5-2.7	<2.5
Total protein (g/dL)	LO	5.5-6.0	5.0-5.4	<5.0
Alkaline phosphatase (U/L)	N	1.1-2 x ULN	2.1-3 x ULN	>3 x ULN
AST (U/L)	HI	1.1-2.5 x ULN	2.6-5 x ULN	>5 x ULN
ALT (U/L)	HI	1.1-2.5 x ULN	2.6-5 x ULN	>5 x ULN
Bilirubin, serum total (mg/dL) – when accompanied by any increase in Liver Function Test		1.1–1.25 x ULN	1.26 –1.5 x ULN	>1.5 x ULN
Bilirubin, serum total (mg/dL) – when Liver Function Test is normal		1.1–1.5 x ULN	1.6–2.0 x ULN	>2.0 x ULN

a Depending upon the laboratory used, reference ranges, eligibility ranges and grading may be split out by sex and/or age.
b Low, High, Not Graded.
c Fasting.
d Non-fasting.

**Blood, Serum, or Plasma Chemistries**[a]	**LO/HI/N** [b]	**Mild (Grade 1)**	**Moderate (Grade 2)**	**Severe (Grade 3)**
Amylase (U/L)	N	1.1-1.5 x ULN	1.6-2.0 x ULN	>2.0 x ULN
Lipase (U/L)	N	1.1-1.5 x ULN	1.6-2.0 x ULN	>2.0 x ULN

**Hematology**	**LO/HI/N** [a]	**Mild (Grade 1)**	**Moderate (Grade 2)**	**Severe (Grade 3)**
Hemoglobin (women) change from baseline (g/dL)	LO	Any decrease-1.5	1.6-2.0	>2.0
Hemoglobin (men) change from baseline (g/dL)	LO	Any decrease-1.5	1.6-2.0	>2.0
White blood cell count (cell/mm^3)	HI	10,800-15,000	15,001-20,000	>20,000
	LO	2,500-3,500	1,500-2,499	<1,500
Lymphocytes (cell/mm^3)	LO	750-1,000	500-749	< 500
Neutrophils (cell/mm^3)	LO	1,500-2,000	1,000-1,499	< 1000
Eosinophils (cell/mm^3)	HI	650-1500	1501-5000	> 5000
Platelets (cell/mm^3)[b]	LO	90,000-99,999	80,000-89,999	<80,000
**Coagulation**				
PT (seconds)	HI	1.0-1.10 x ULN	1.11-1.20 x ULN	>1.20 x ULN
International Normalized Ratio (INR)[c]	HI	1.1-1.5 x ULN	1.6-2.0 x ULN	>2.0 x ULN
PTT or aPTT (seconds)	HI	1.0-1.2 x ULN	1.21-1.4 x ULN	>1.4 x ULN
Fibrinogen (mg/dL)	HI	400-500	501-600	>600
	LO	150-200	125-149	<125
**Urine**				
Protein (dipstick)	HI	Trace	1+	2+
Glucose (dipstick)	HI	Trace	1+	2+
Blood (microscopic) - red blood cells per high power field (RBC/HPF)	HI	1-10	11-50	>50 and/or gross blood

[a] Low, High, Not Graded.
[b] Platelet values are based on Dosoo et al. (2012) [3]
[c] For INR, the values in the table are based on the Division of AIDS Table for Grading the Severity of Adult and Pediatric Adverse Events, 2009.

VITAL SIGNS TOXICITY GRADING

Grading scale used for vital signs is according to DMID Toxicity Tables (2014)

Vital Signs	LO/HI/N [a]	Mild (Grade 1) [b]	Moderate (Grade 2)	Severe (Grade 3)
Fever (°C)[c]	HI	38.0-38.4	38.5-38.9	>38.9
Fever (°F)	HI	100.4-101.1	101.2-102.0	>102.0
Tachycardia	HI	101-115 bpm	116-130 bpm	>130 bpm or ventricular dysrhythmias
Bradycardia	LO	50-54 or 45-50 bpm if baseline <60 bpm	45-49 or 40-44 bpm if baseline <60 bpm	<45 or <40 bpm if baseline <60 bpm
Hypertension (systolic) - mm Hg[d]	HI	141-150	151-160	>160
Hypertension (diastolic) - mm Hg	HI	91-95	96-100	>100
Hypotension (systolic) - mm Hg	LO	85-89	80-84	<80
Tachypnea - breaths per minute	HI	23-25	26-30	>30

a Low, High, Not Graded.
b If initial bound of grade 1 has gap from reference range or eligibility range, calculations based on the New England Journal of Medicine (NEJM) reference ranges.
c Axillary temperature.
d Assuming subject is awake, resting, and supine; for adverse events, 3 measurements on the same arm with concordant results.

Attachment 5: Toxicity Tables for Use in Trials Enrolling Children Greater Than 3 Months of Age

The abbreviations used in the following tables are:

ALT: alanine aminotransferase; AST: aspartate aminotransferase; CNS: central nervous system; CK: creatine kinase; hpf: high power field; GGT: gamma glutamyltransferase; mEq: milliequivalent; PT: prothrombin time; PTT: partial prothrombin time; ULN: upper limit of normal

CLINICAL ADVERSE EVENTS

Grading scale used for clinical adverse events is adapted from the DMID Pediatric Toxicity Tables for Children Greater Than 3 Months of Age (2007). For adverse events not included in the tables below, refer to the severity criteria guidelines in Section 12.1.3.

**Gastrointestinal**	**Grade 1**	**Grade 2**	**Grade 3**
Nausea/vomiting	Minimal symptoms; caused minimal or no interference with work, school or self-care activities	Notable symptoms; required modification in activity or use of medications; did not result in loss of work or cancellation of social activities	Incapacitating symptoms; required bed rest and/or resulted in loss of work or cancellation of social activities
Diarrhea	Slight change in consistency and/or frequency of stools	Liquid stools	Liquid stools greater than 4x the amount or number normal for the child
Appetite	-	Decreased appetite	Appetite very decreased, no solid food taken
Abdominal Pain	Mild	Moderate; no treatment needed	Moderate; treatment needed
Constipation	Slight change in consistency/frequency of stool	Hard, dry stools with a change in frequency	Abdominal pain
**Reactogenicity**	**Grade 1**	**Grade 2**	**Grade 3**
Local reactions			
Pain/tenderness at injection site	Aware of symptoms but easily tolerated; does not interfere with activity; discomfort only to touch	Notable symptoms; required modification in activity or use of medications; discomfort with movement	Incapacitating symptoms; inability to do work or usual activities; significant discomfort at rest
Erythema/redness	< 10 mm	10-25 mm	26-50 mm
Induration/swelling	< 10 mm	10-25 mm	26-50 mm
Itching at the injection site	Infrequent, brief episode of scratching, easily distracted from scratching	Frequent, longer episodes of scratching, difficult to distract	Near constant scratching, or scratching during sleep; excoriation of skin
Edema	< 10 mm	10-25 mm	26-50 mm
Rash at the injection site	< 10 mm	10-25 mm	26-50 mm
Systemic reactions			
Allergic reaction	Pruritus without rash	Pruritic rash	Mild urticaria

**Reactogenicity (continued)**	**Grade 1**	**Grade 2**	**Grade 3**
Headache	Minimal symptoms; caused minimal or no interference with work, school or self-care activities	Notable symptoms; required modification in activity or use of medications; did not result in loss of work/school or cancellation of social activities	Incapacitating symptoms; required bed rest and/or resulted in loss of work/school or cancellation of social activities
Fatigue/malaise	Minimal symptoms; caused minimal or no interference with work, school or self-care activities	Notable symptoms; required modification in activity or use of medications; did not result in loss of work/school or cancellation of social activities	Incapacitating symptoms; required bed rest and/or resulted in loss of work/school or cancellation of social activities
Myalgia	Minimal symptoms; caused minimal or no interference with work, school or self-care activities	Notable symptoms; required modification in activity or use of medications; did not result in loss of work/school or cancellation of social activities	Incapacitating symptoms; required bed rest and/or resulted in loss of work/school or cancellation of social activities
Arthralgia	Minimal symptoms; caused minimal or no interference with work, school or self-care activities	Notable symptoms; required modification in activity or use of medications; did not result in loss of work/school or cancellation of social activities	Incapacitating symptoms; required bed rest and/or resulted in loss of work/school or cancellation of social activities
Chills	Minimal symptoms; caused minimal or no interference with work, school or self-care activities	Notable symptoms; required modification in activity or use of medications; did not result in loss of work/school or cancellation of social activities	Incapacitating symptoms; required bed rest and/or resulted in loss of work/school or cancellation of social activities
**Central Nervous System (CNS)**	**Grade 1**	**Grade 2**	**Grade 3**
Generalized CNS Symptoms			Dizziness
Level of activity		Slightly irritable OR slightly subdued	Very irritable OR lethargic
Visual		Blurriness, diplopia, or horizontal nystagmus of <1 hour duration, with spontaneous resolution	More than 1 episode of Grade 2 symptoms per week, or an episode of Grade 2 symptoms lasting more than 1 hour with spontaneous resolution by 4 hours, or vertical nystagmus
Myelopathy		None	None

**Peripheral Nervous System**	**Grade 1**	**Grade 2**	**Grade 3**
Neuropathy/Lower Motor Neuropathy		Mild transient paresthesia only	Persistent or progressive paresthias, burning sensation in feet, or mild dyesthesia; no weakness; mild to moderate deep tendon reflex changes; no sensory loss
Myopathy or Neuromuscular Junction Impairment	Normal or mild (<2 x ULN) CK elevation	Mild proximal weakness and/or atrophy not affecting gross motor function. Mild myalgias with/without mild CK elevation (<2 x ULN)	Proximal muscle weakness and/or atrophy affecting motor function with/without CK elevation; or severe myalgias with CK>2 x ULN
**Other**	**Grade 1**	**Grade 2**	**Grade 3**
Fever	38.0-38.4 °C or 100.4-101.1 °F	38.5-40 °C or 101.2-104.0 °F	Greater than 40 °C or 104.0 °F
Cutaneous	Localized rash	Diffuse maculopapular rash	Generalized urticaria
Stomatitis	Mild discomfort	Painful, difficulty swallowing, but able to eat and drink	Painful: unable to swallow solids
Clinical symptom not otherwise specified in this table	No therapy; monitor condition	May require minimal intervention and monitoring	Requires medical care and possible hospitalization
Laboratory values not otherwise specified in this table	Abnormal, but requiring no immediate intervention; monitor	Sufficiently abnormal to require evaluation as to causality and perhaps mild therapeutic intervention, but not of sufficient severity to warrant immediate changes in study vaccine	Sufficiently severe to require evaluation and treatment, including at least temporary suspension of study vaccine

LABORATORY TOXICITY GRADING

**Serum chemistry**	**Mild (Grade 1)**	**Moderate (Grade 2)**	**Severe (Grade 3)**
Bilirubin (when accompanied by any increase in other liver tests)	1.1-<1.25 x ULN	1.25-<1.5 x ULN	1.5-1.75 x ULN
Bilirubin (when other liver function tests are in normal range)	1.1-<1.5 x ULN	1.5-<2.0 x ULN	2.0-3.0 x ULN
AST	1.1-<2.0 x ULN	2.0-<3.0 x ULN	3.0-8.0 x ULN
ALT	1.1-<2.0 x ULN	2.0-<3.0 x ULN	3.0-8.0 x ULN
GGT	1.1-<2.0 x ULN	2.0-<3.0 x ULN	3.0-8.0 x ULN
Pancreatic amylase	1.1-1.4 x ULN	1.5-1.9 x ULN	2.0-3.0 x ULN
Uric acid	7.5-9.9 mg/dL	10-12.4 mg/dL	12.5-15.0 mg/dL
CK	See Neuromuscular Toxicity		
Creatinine 3 months – 2 years of age	0.6-0.8 x ULN	0.9-1.1 x ULN	1.2-1.5 x ULN
Creatinine 2 – 12 years of age	0.7-1.0 x ULN	1.1-1.6 x ULN	1.7-2.0 x ULN
Creatinine >12 years of age	1.0-1.7 x ULN	1.8-2.4 x ULN	2.5-3.5 x ULN
Hypernatremia	-	<145-149 mEq/L	150-155 mEq/L
Hyponatremia	-	130-135 mEq/L	129-124 mEq/L
Hyperkalemia	5.0-5.9 mEq/L	6.0-6.4 mEq/L	6.5-7.0 mEq/L
Hypokalemia	3.0-3.5 mEq/L	2.5-2.9 mEq/L	2.0-2.4 mEq/L
Hypercalcemia	10.5-11.2 mg/dL	11.3-11.9 mg/dL	12.0-12.9 mg/dL
Hypocalcemia	7.8-8.4 mg/dL	7.0-7.7 mg/dL	6.0-6.9 mg/dL
Hypomagnesemia	1.2-1.4 mEq/L	0.9-1.1 mEq/L	0.6-0.8 mEq/L
Hyperglycemia	116-159 mg/dL	160-249 mg/dL	250-400 mg/dL
Hypoglycemia	55-65 mg/dL	40-54 mg/dL	30-39 mg/dL
**Hematology**	**Mild (Grade 1)**	**Moderate (Grade 2)**	**Severe (Grade 3)**
Hemoglobin for children >3 months and <2 years of age	9.0-9.9 g/dL	7.0-8.9 g/dL	<7.0 g/dL
Hemoglobin for children >2 years of age	10.0-10.9 g/dL	7.0-9.9 g/dL	<7.0 g/dL
Absolute neutrophil count	750-1200/mm^3	400-749/mm^3	250-399/mm^3
Platelets	-	50,000-75,000/mm^3	25,000-49,999/mm^3
PT	1.1-1.2 x ULN	1.3-1.5 x ULN	1.6-3.0 x ULN
PTT	1.1-1.6 x ULN	1.7-2.3 x ULN	2.4-3.0 x ULN

Urinalysis	Mild (Grade 1)	Moderate (Grade 2)	Severe (Grade 3)
Proteinuria	1+ or < 150 mg/day	2+ or 150-499 mg/day	3+ or 500-1000 mg/day
Hematuria	Microscopic <25 cells/hpf	Microscopic >25 cells/hpf	-

INVESTIGATOR AGREEMENT

I have read this protocol and agree that it contains all necessary details for carrying out this study. I will conduct the study as outlined herein and will complete the study within the time designated.

I will provide copies of the protocol and all pertinent information to all individuals responsible to me who assist in the conduct of this study. I will discuss this material with them to ensure that they are fully informed regarding the study vaccine, the conduct of the study, and the obligations of confidentiality.

Coordinating Investigator (where required):

Name (typed or printed): ______________________

Institution and Address: ______________________

____

____

____

Signature: ______________________ Date: ______________________

(Day Month Year)

Principal (Site) Investigator:

Name (typed or printed): ______________________

Institution and Address: ______________________

____

____

____

Telephone Number: ______________________

Signature: ______________________ Date: ______________________

(Day Month Year)

Sponsor's Responsible Medical Officer:

Name (typed or printed): [redacted]

Institution: [redacted]

Signature: [redacted] Date: ______________________

(Day Month Year)

Note: If the address or telephone number of the investigator changes during the course of the study, written notification will be provided by the investigator to the sponsor, and a protocol amendment will not be required.

LAST PAGE
